# Structural basis for ion selectivity in potassium-selective channelrhodopsins

**DOI:** 10.1016/j.cell.2023.08.009

**Published:** 2023-08-30

**Authors:** Seiya Tajima, Yoon Seok Kim, Masahiro Fukuda, YoungJu Jo, Peter Y. Wang, Joseph M. Paggi, Masatoshi Inoue, Eamon F.X. Byrne, Koichiro E. Kishi, Seiwa Nakamura, Charu Ramakrishnan, Shunki Takaramoto, Takashi Nagata, Masae Konno, Masahiro Sugiura, Kota Katayama, Toshiki E. Matsui, Keitaro Yamashita, Suhyang Kim, Hisako Ikeda, Jaeah Kim, Hideki Kandori, Ron O. Dror, Keiichi Inoue, Karl Deisseroth, Hideaki E. Kato

**Affiliations:** 1 Komaba Institute for Science, The University of Tokyo, Meguro, Tokyo, Japan; 2 Department of Bioengineering, Stanford University, Stanford, CA, USA; 3 Department of Computer Science, Stanford University, Stanford, CA, USA; 4 CNC Program, Stanford University, Stanford, CA, USA; 5 The Institute for Solid State Physics, The University of Tokyo, Kashiwa, Japan; 6 PRESTO, Japan Science and Technology Agency, Kawaguchi, Saitama, Japan; 7 Department of Life Science and Applied Chemistry, Nagoya Institute of Technology, Showa-ku, Japan; 8 MRC Laboratory of Molecular Biology, Cambridge Biomedical Campus, Cambridge, UK; 9 OptoBioTechnology Research Center, Nagoya Institute of Technology, Showa-ku, Japan; 10 Institute for Computational and Mathematical Engineering, Stanford University, Stanford, CA, USA; 11 Howard Hughes Medical Institute, Stanford University, Stanford, CA, USA; 12 Department of Psychiatry and Behavioral Sciences, Stanford University, Stanford, CA, USA; 13 Department of Biological Sciences, Graduate School of Science, The University of Tokyo, Bunkyo, Tokyo, Japan; 14 FOREST, Japan Science and Technology Agency, Kawaguchi, Saitama, Japan

## Abstract

KCR channelrhodopsins (K^+^-selective light-gated ion channels) have received attention as potential inhibitory optogenetic tools but more broadly pose a fundamental mystery regarding how their K^+^ selectivity is achieved. Here, we present 2.5–2.7 Å cryo-electron microscopy structures of *Hc*KCR1 and *Hc*KCR2 and of a structure-guided mutant with enhanced K^+^ selectivity. Structural, electrophysiological, computational, spectroscopic, and biochemical analyses reveal a distinctive mechanism for K^+^ selectivity; rather than forming the symmetrical filter of canonical K^+^ channels achieving both selectivity and dehydration, instead, three extracellular-vestibule residues within each monomer form a flexible asymmetric selectivity gate, while a distinct dehydration pathway extends intracellularly. Structural comparisons reveal a retinal-binding pocket that induces retinal rotation (accounting for *Hc*KCR1/*Hc*KCR2 spectral differences), and design of corresponding KCR variants with increased K^+^ selectivity (KALI-1/KALI-2) provides key advantages for optogenetic inhibition *in vitro* and *in vivo*. Thus, discovery of a mechanism for ion-channel K^+^ selectivity also provides a framework for next-generation optogenetics.

## Graphical abstract

**Graphical abstract:**
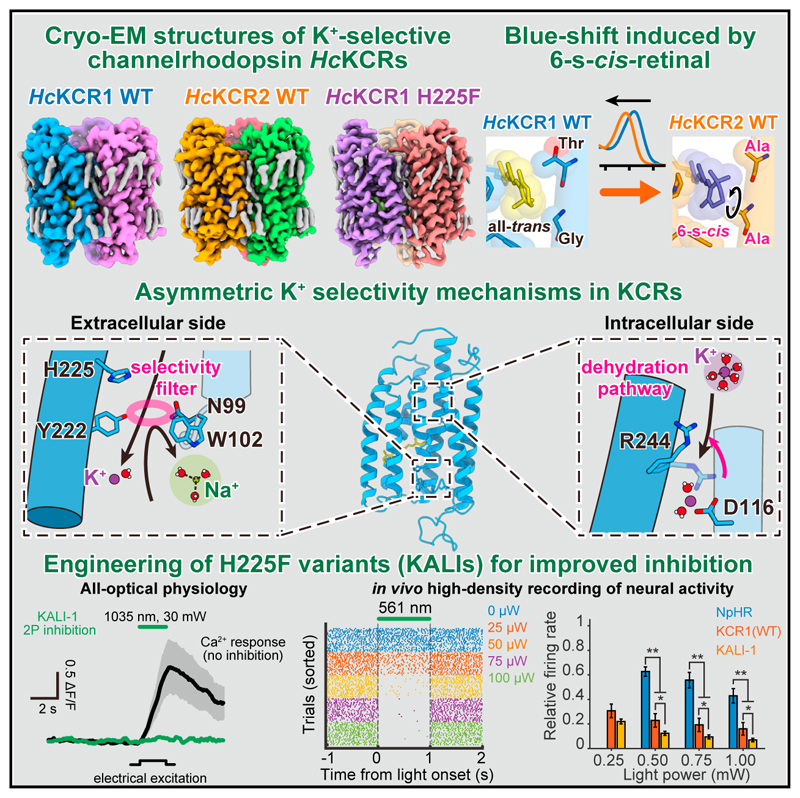

## Introduction

Motile organisms sense light, an important energy source and environmental signal, using rhodopsin family proteins. Rhodopsins are classified into microbial and animal types,^1^ both containing a seven-helix transmembrane (7TM) domain (opsin) covalently bound to a chromophore (retinal). Microbial rhodopsins undergo retinal isomerization upon light absorption (from *all-trans* to *13-cis*), leading to diverse ion-pump, ion-channel, sensor, and enzymatic functionalities.^2,3^ When combined with precise light and gene delivery, expression of these proteins (especially channel- and pump-type rhodopsins) enables control of membrane potential in specific cells within animals with high spatiotemporal resolution. This experimental approach (optogenetics) has been applied to studies of neural circuitry and organismal physiology and to treatment of human diseases.^4–7^

Cation-conducting channelrhodopsins (cation ChRs or CCRs, fluxing monovalent and divalent cations) from chlorophyte algae were first used in optogenetics^4^ to excite neurons with light.^5,8–12^ Following identification of channelrhodopsins (ChRs) in 2002,^13^ many variants with unique properties were engineered or discovered, expanding the optogenetic toolbox for excitation.^6^ Optogenetic inhibition was shown using Cl^−^ pumps and H^+^ pumps,^14,15^ with more potent inhibition later achieved using designed and natural anion-conducting channelrhodopsins (anion ChRs or ACRs),^16–18^ now used across many organisms.^19–23^ However, developmental or anatomical variations in [Cl^−^] gradients can lead to unintended neuronal excitation, complicating some ACR applications.^24,25^

Since physiological neuronal membrane deactivation by repolarization involves K^+^ efflux, creating K^+^-selective ChRs could be considered for neuronal silencing^25^; however, previously known K^+^ channels exhibit conserved organization with no similarity to ChRs (Figure S1). Canonical K^+^ channels, like KcsA (tetramer)^26^ or TRAAK (dimer),^27^ use a fourfold-symmetric selectivity filter with a highly conserved TVGYG or related motif^28^ (Figures S1B–S1D). Even the recently discovered H^+^/K^+^ channel TMEM175 maintains a fourfold-symmetric selectivity filter despite lacking the TVGYG motif^29–32^ (Figures S1B–S1D); in contrast, ChRs form oligomers (dimers or trimers), but the ionconducting pathway is within each monomer and highly asymmetric^2^ (Figures S1B–S1D, left).

Recently, the third family of channelrhodopsins, termed pump-like channelrhodopsins (PLCRs, or bacteriorhodopsin-like channelrhodopsins), was discovered in cryptophyte algae and marine metagenomic datasets; PLCRs show greater sequence similarity to pump-type (Cl^−^ and H^+^) rhodopsins than canonical ChRs^33–36^ but function instead as channels with distinctive properties. For example, the PLCR ChRmine exhibits extremely high photocurrents, high light sensitivity, and a red-shifted action spectrum.^34,37^ Moreover, some PLCRs, such as ChRmine and CCR4 from *Guillardia theta* (*Gt*CCR4), are selective for monovalentions.^38,39^

Two microbial rhodopsins were identified as light-gated K^+^- selective channels.^40^ These KCR rhodopsins from *Hyphochytrium catenoides* (*Hc*KCR1 and *Hc*KCR2), are homologous by sequence to previously discovered PLCRs^38,41^ but exhibit higher K^+^/Na^+^ permeability ratios (*P*_K_/*P*_Na_) of ~23 and ~17, respectively, surpassing other PLCRs (e.g., *P*_K_/*P*_Na_ of both *Gt*CCR4 and ChRmine = ~0.9),^39^ CCRs (e.g., *Chlamydomonas reinhardtii* ChR2 [*Cr*ChR2] *P*_K_/*P*_Na_ = ~0.5),^42^ and some canonical K^+^ channels (e.g., *P*_K_/*P*_Na_ of mouse SLO3 and viral Kcv exhibit *P*_K_/*P*_Na_ of 5–10 and ~9, respectively).^43,44^ KCRs thus show promise as inhibitory optogenetic tools.^40^ However, the mechanism of K^+^ selectivity in KCRs remains elusive, and insight would be enormously valuable, not only as a general paradigm for understanding how ion-channel proteins can achieve K^+^ selectivity but also for developing next-generation KCR-based optogenetic tools.

## Results

### Overall structural comparison between *Hc*KCR1, *Hc*KCR2, and ChRmine

We expressed *Hc*KCR1 and *Hc*KCR2 in Sf9 insect cells and reconstituted purified proteins into lipid nanodiscs (STAR Methods); cryo-EM structures in the dark state were resolved at 2.6 and 2.5 Å, respectively (Figures S2A–S2H; Table S1). The high-resolution density maps allowed accurate modeling of most residues of both *Hc*KCRs (residues 6–260 for *Hc*KCR1 and 2–260 for *Hc*KCR2), as well as water molecules, lipids, and all-*trans*-retinal with conformer also validated by high-performance liquid chromatography (HPLC) (Figures S2I–S2P, S3A, and S3B; Data S1). In *Hc*KCR2, the N-terminal P2 is surrounded by P95, F96, W100, and Y101 with no space for the first methionine (Figure S2N), consistent with its predicted post-translational cleavage.^45^

**Figure 1:**
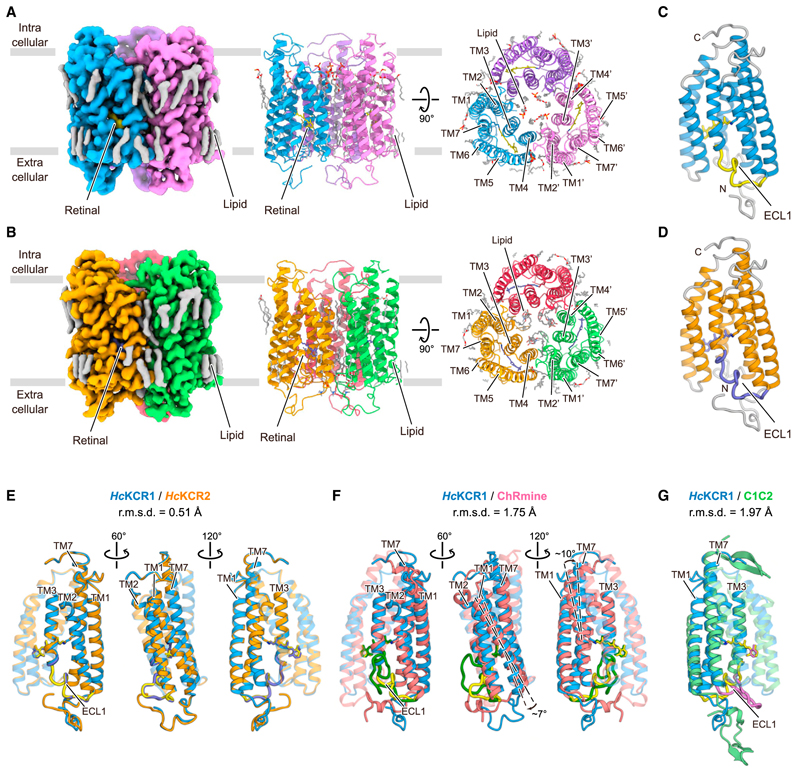
Cryo-EM structures of WT *Hc*KCR1 and *Hc*KCR2 (A) Cryo-EM density map (left) and ribbon representation of *Hc*KCR1 homotrimer viewed parallel to membrane (middle) and from intracellular side (right). (B) Cryo-EM density map (left) and ribbon representation of *Hc*KCR2 homotrimer viewed parallel to membrane (middle) and from intracellular side (right). (C and D) Monomeric structures of *Hc*KCR1 (C) and *Hc*KCR2 (D). (E–G) Structural comparisons of *Hc*KCR1 (blue) with *Hc*KCR2 (orange) (E), ChRmine (red) (F), and C1C2 (green) (G) from different angles.

Both *Hc*KCR1 and 2 form trimers, as in ChRmine.^36,38,46^ Trimerization occurs through direct lipid-mediated interactions among transmembrane helices (TMs) 1–2 and 4–5 of adjacent protomers, and the center of the interface is filled with lipid molecules (Figures 1A and 1B). The monomers exhibit an extracellular N-terminal region (residues 6–21 for *Hc*KCR1 and 2–21 for *Hc*KCR2), an intracellular C-terminal region (residues 255–260 for both), and 7 TM domains (within residues 22–254 for both), connected by three intracellular loops (ICL1–3) and three extra-cellular loops (ECL1–3) (Figures 1C and 1D). The overall structures of *Hc*KCR1 and 2 are almost identical with only minor differences in the N-terminal region, ICLs, and ECLs (Figure 1E).

*Hc*KCRs also superpose onto ChRmine, but with critical structural differences (Cα root mean square deviation [r.m.s.d.] is 1.75 Å) (Figure 1F). First, in ChRmine, both N- and C-terminal regions have short α-helices parallel to the membrane, absent in the *Hc*KCRs (Figure 1F). Second, ICLs and ECLs, except for ECL3, exhibit different conformations; the *Hc*KCR ECL1, a domain that distinguishes PLCRs from other ChR families, is ~6 residues shorter than in ChRmine and tightly packed to the helix bundle core (Figure 1F). Third, TM1 and the C-terminal half of TM7 are tilted about 7° and 10°, respectively, relative to the helical bundle. The C-terminal TM7 helix is also ~1.5 turns longer than that of ChRmine (Figure 1F), resembling canonical CCRs (Figure 1G). These structural differences alter the shape of the ion-conducting pathway within each monomer (discussed in “Ion-conducting pore and K^+^ selectivity” below).

### The Schiff base region

Microbial rhodopsins have an all-*trans*-retinal molecule covalently bound to a conserved TM7 lysine via a Schiff base. In the dark, the Schiff base is protonated and stabilized by nearby acidic residues.^47^ Initial reactions triggered by light absorption include retinal isomerization and subsequent proton transfer from the Schiff base to a nearby acidic residue or water molecule. The residues stabilizing the Schiff base proton and receiving the proton in the photo-intermediate state (M intermediate) are termed the Schiff base counterion(s) and the proton acceptor, respectively.^48^ The architecture of this region affects key properties of microbial rhodopsins^48^ and is thus a critical focus here.

**Figure 2:**
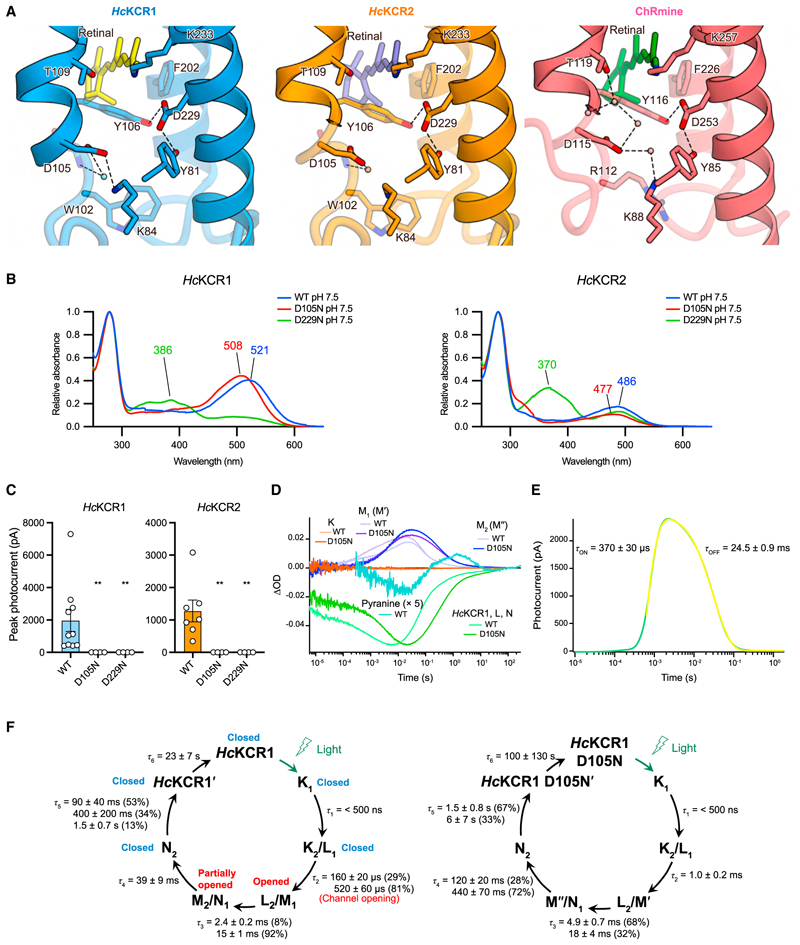
The Schiff base region (A) Schiff base regions of *Hc*KCR1 (left), *Hc*KCR2 (middle), and ChRmine (right). Water molecules are represented by spheres. Black dashes indicate H-bonds. (B) Absorption spectra of *Hc*KCR1 (left) and *Hc*KCR2 (right) at pH 7.5. (C) Photocurrentamplitudes of WT, D105N, and D229N of *Hc*KCR1 (left) and *Hc*KCR2 (right), respectively. Mean ± s.e.m. (n = 4–10; **p < 0.01; Kruskal-Wallistest with Dunnett’s test). (D) Time-series traces of absorption change for *Hc*KCR1 WT and D105N mutant. (E) Transient photocurrent changes of *Hc*KCR1 induced by pulsed laser, with raw trace (green) and fitting curve (yellow) indicated. (F) Photocycles of *Hc*KCR1 WT (left) and D105N (right) determined in (D).

**Figure 3:**
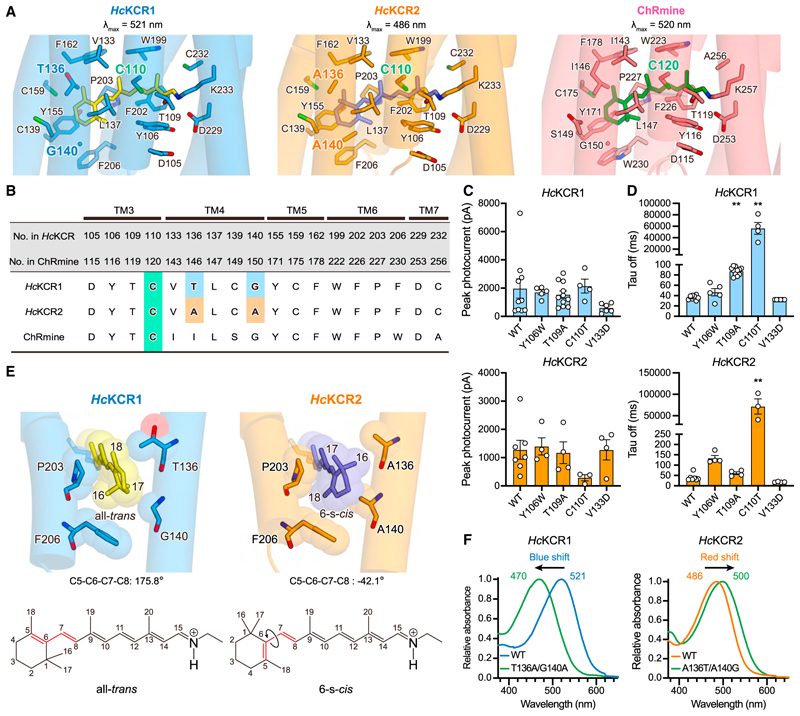
Retinal binding pocket (A) Retinal binding pockets of *Hc*KCR1 (left), *Hc*KCR2 (middle), and ChRmine (right) with pocket-forming residues shown in stick models. (B) Sequence alignment for residues in retinal binding pocket. (C) Peak photocurrent amplitudes ofWT and four mutants of *Hc*KCR1 (top) and *Hc*KCR2 (bottom), respectively. Mean ± s.e.m. (n = 3–11; Kruskal-Wallistestwith Dunnett’s test). (D) τ_off_ of WT and four mutants of *Hc*KCR1 (top) and *Hc*KCR2 (bottom), respectively. Mean ± s.e.m. (n = 3–11; Kruskal-Wallis test with Dunnett’s test; **p < 0.01). (E) β-ionone rings of *Hc*KCR1 and *Hc*KCR2 (top) and chemical structures of all-*trans* and 6-*s-cis*-retinal (bottom). Red lines represent C_5_–C_6_–C_7_–C_8_ bonds. (F) Absorption spectra of *Hc*KCR1 and 2 WT and their swapping mutants.

We previously revealed features in the Schiff base region of ChRmine distinct from those of other microbial rhodopsins^38^; TM3 is unwound in the middle of the membrane, and two aspartates (potential counterion and proton acceptor) appear on TM3/ ECL1 and TM7. One aspartate (D115) faces away from the Schiff base proton, and the other (D253) forms H-bonds with Y85/Y116 (Figure 2A, right). These features were shared in *Hc*KCRs, suggesting a conserved architecture among PLCRs (Figures 2A and S4A). However, we noted three key differences in *Hc*KCRs: (1) K84 points toward the extracellular side (Figures 2A and S2K), (2) no water molecules are observed between the Schiff base proton and the two aspartates (D105 and D229) (Figure 2A), and (3) the highly conserved arginine (Figure S1A) on ECL1 (R112 in ChRmine) is replaced by a tryptophan (W102 in *Hc*KCRs) (Figures 2A and S1A). These differences prompted further characterization of D105 and D229.

First, to assign protonation states to these two aspartates and identify the primary counterion, we measured absorption spectra of wild-type (WT), D105N, and D229N mutants of both *Hc*KCRs (Figures 2B, S3C, and S3D). λ_max_ values of WT, D105N, and D229N mutants of *Hc*KCR1 at neutral pH were 521, 508, and 386 nm, respectively, demonstrating that protonation of D105 causes only a small blue shift (~13 nm); protonation of D229 causes a larger blue shift (~135 nm), indicating concomitant deprotonation of the Schiff base nitrogen (Figure 2B, left). Similar trends were also observed in *Hc*KCR2 (Figure 2B, right). These findings initially suggested that both D105 and D229 are deprotonated in the dark state, with only D229 serving as the primary counterion to stabilize the positive charge of the Schiff base proton—contrasting with ChRmine, in which both corresponding aspartate residues (D115 and D253) are essential counterions of the Schiff base proton.^38^

However, our electrophysiology surprisingly revealed that channel function is completely abolished for both D229N and D105N mutants (Figures 2C and S5B). We conducted laser flash-photolysis and laser patch-clamp experiments (Figures 2D–2F, S3E, and S3F), revealing that *Hc*KCR1 has eight intermediates (K_1_, K_2_, L_1_, L_2_, M_1_, M_2_, N_1_, and N_2_) in its photocycle, with M_1_ and M_2_ representing the open state.^40^ Further investigation of the D105N photocycle showed that rise and decay of the M intermediate become slower in this mutant (Figures 2D and 2F), suggesting that D105 works as the proton acceptor. This interpretation is also supported by the pH-sensitive pyranine assay,^49^ showing that the Schiff base proton is released to bulk solvent later than the rise of the M_1_ intermediate (Figure 2D); this result indicates that the Schiff base proton is not directly released to water but is transferred to an acidic residue in the Schiff base region. Notably, the absorption spectra of M_1_ and M_2_ intermediates in the D105N mutant are different from those in WT (Figure S3F), suggesting that the structures of the D105N mutant at these intermediates, denoted as M’ and M” (Figure 2F), are also different from WT and compromise channel function. Overall, these results suggest that D105 does not work as the counterion in the dark state but rather as the proton acceptor in the M intermediate and that proton transfer to D105 would be an important step for correct channel gating.

### The retinal binding pocket

The residues surrounding the retinal chromophore are essential for key ChR properties, including kinetics and absorption spectrum.^50–52^ We next focused on this area and found that *Hc*KCRs and ChRmine share highly similar residues in the retinal binding pocket (Figure 3A); 12 of 18 residues are conserved among *Hc*KCRs and ChRmine, and only two residues are different between *Hc*KCR1 and 2 (Figure 3B). We introduced mutations to Y106 and T109 in *Hc*KCRs (Y116 and T119 in ChRmine) because a previous study showed that Y116W and T119A in ChRmine decelerated and accelerated off-kinetics, respectively.^46^ However, corresponding mutations in *Hc*KCRs showed distinct results; the Y106W mutation moderately decelerated off-kinetics only in *Hc*KCR2, and the T109A mutation did not accelerate but rather decelerated off-kinetics only in *Hc*KCR1 (Figures 3C, 3D, S5, S6A, and S6B). Y106 and T109 are part of both the retinal binding pocket and the Schiff base region (Figure 2A), suggesting that the differences in the Schiff base could account for the distinct mutational effects observed.

We next introduced mutations to C110 and V133 of the type we had previously shown to prolong off-kinetics in *Cr*ChR2,^50,53,54^ which gave rise to the step-function opsins (SFOs) that enable prolonged cell-type-specific control with brief light pulses.^4,5^ Although previous attempts to transfer this functionality to PLCRs were unsuccessful,^55^ we discovered that the outcome was strikingly successful for *Hc*KCR1 and 2 (Figures 3C and 3D). The C110T mutation increased the τ_off_ of *Hc*KCR1 and 2 by ~1500- and ~1800-fold, respectively (Figure 3D); notably, the *Hc*KCR1 C110T retained comparable photocurrent size (Figure 3C). This study thus provides the initial step-function opsin arising from any PLCR, with *Hc*KCR1 C110T showing potential as an optogenetic tool for long-timescale inhibition.

*Hc*KCR1 and 2 exhibit different spectral properties; λ_max_ of *Hc*KCR1 and 2 are 521 nm and 486 nm, respectively (Figure 2B). Despite similarity in retinal binding pockets, these differ at positions 136 and 140 near the β-ionone ring of the retinal (Figures 3A and 3B), offering an opportunity to investigate spectral mechanisms. While 6-*s*-*trans*-retinal is found in all reported structures of naturally occurring microbial rhodopsins (Figure S4B), during the structural refinement of *Hc*KCR2 with 6-*s*-*trans*-retinal (STAR Methods), we observed a steric clash between the C_17_ atom of retinal and the methyl group of A140, along with extra density next to the C_18_ atom (Figure S2P, top). Modeling 6-*s-cis*-retinal resolved this issue (Figure S2P, bottom), indicating that these two residues (A136/A140 in *Hc*KCR2) create a steric clash with the C_17_ atom and simultaneously make space to accommodate the C_16_ atom to induce the rotation of the β-ion-one ring (Figure 3E). The ~220° rotation of the ring shrinks the π-conjugated system of retinal and thereby induces a ~35 nm spectral shift (Figures 2B and 3E). This aligns with a previous study showing that a designed ChR with glycine and alanine at the same positions, C1C2GA, exhibits retinal rotated by ~210° and a spectrum blue-shifted by ~20 nm (Figure S4B).^56^ Swapping these two residues between *Hc*KCR1 and *Hc*KCR2 confirmed their influence on the orientation of the β-ionone ring and largely explained the spectral difference (Figure 3F). *Hc*KCR2 thus represents the initial naturally occurring microbial rhodopsin with experimentally demonstrated 6-*s*-*cis*-retinal configuration.

### Ion-conducting pore and K^+^ selectivity

The three major classes of ChRs (including the PLCRs),^21,38,57,58^ although all assembling as multimers, exhibit ion-conducting pores within each monomer formed by TM1, 2, 3, and 7. For example, the PLCR ChRmine forms a trimer,^38^ and although mutations in the center of this trimer were found to modulate ion selectivity, this central region was not predicted to form a conducting pore in Kishi et al. (2022)^38^; rather, each monomer forms its own transmembrane pore.^38^ In the dark state, the pore is divided into intracellular and extracellular vestibules (IVs and EVs) by intracellular, central, and extracellular constriction sites (ICSs, CCSs, and ECSs).^2^

**Figure 4:**
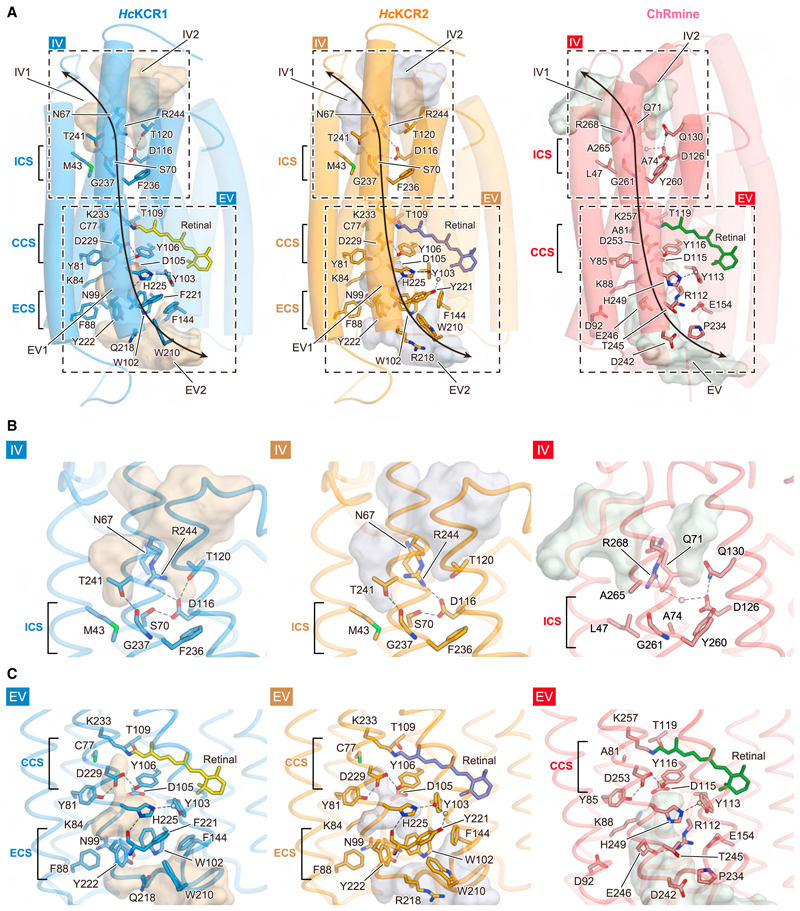
Ion-conducting cavities (A) Comparison of ion-conducting cavities among *Hc*KCR1 (left), *Hc*KCR2 (middle), and ChRmine (right). TMs 4–6 are displayed with higher transparency. Residues located along the cavities are shown in stick form. Black dashed rectangles indicate IV and EV regions highlighted in (B) and (C), respectively. Black arrows represent the putative ion-conducting pathway. (B and C) IV (B) and EV (C) of *Hc*KCR1 (left), *Hc*KCR2 (middle), and ChRmine (right). Cavities are calculated with HOLLOW, and black dashes indicate H-bonds. Locations of ICS, CCS, and ECS are indicated on the left in each panel.

*Hc*KCRs have primary sequences similar to archaeal pump-type rhodopsins (Figure S1A) but exhibit larger cavities due to structural differences among the pore-forming helices (Figures 4A and S4C). Due to TM3 unwinding, not only TM1, 2, 3, and 7 but also ECL1 shape the EV, as observed in ChRmine (Figures 1F and 4A).^38^ Three notable differences from ChRmine were observed. First, although *Hc*KCRs and ChRmine exhibit two IVs (IV1 and IV2) divided by a TM7 arginine (R244 in *Hc*KCRs and R268 in ChRmine) and both are occluded at the ICS, the CS interaction network is different. In ChRmine, R268 forms H-bonds with Q71, and D126 forms H-bonds with both Q130 and Y260 as well as a water-mediated H-bond with Q71. This H-bond network, together with L47, A74, and G261, defines the ICS (Figure 4B, right). In contrast, in *Hc*KCRs, R244 approaches TM3 due to the ~10° tilt of the cytoplasmic half of TM7 (Figure 1F) and forms a salt bridge with D116 (D126 in ChRmine). Moreover, A74, Q130, and Y260 in ChRmine are replaced by S70, T120, and F236, respectively, resulting in rearrangement of the H-bond network centered on D116 (Figure 4B, left/middle).

Second, the EV in *Hc*KCRs extends deeper into the core bundle, reaching the Schiff base (Figure 4C). While the Schiff base architecture is similar (Figure 2A), the small conformational difference of K84 enlarges the pore in *Hc*KCRs, and the EV extends close to the Schiff base lysine (K233), similar to *Gt*ACR1 (Figure S4C). Thus, not only the counterion complexes (D105, D229, Y81, and Y106) but also C77, T109, and K233 contribute to defining the CCS in *Hc*KCRs (Figure 4C, left/middle).

Finally, several hydrophilic residues that line the EV in ChRmine, including D92, R112, E154, T245, and E246, are replaced by aromatic residues (F88, W102, F144, F/Y221, and Y222), which make the surface more hydrophobic (Figure 4C, left/middle). Moreover, W102 and Y222 form a new constriction (ECS) with ECL1 positioned closer to the core of the helix bundle compared to that of ChRmine (Figure 1F) and allow N99 on ECL1 to H-bond with Y222, separating the EV into two cavities (Figure 4C, left/middle). The replacement of arginine with tryptophan (W102) in *Hc*KCRs also causes a rotameric change of histidine (H225 in *Hc*KCRs) and generates a new H-bond between H225 and F/Y221 (Figure 4C, left/middle). Overall, these aromatic residues create unique EVs with shape and properties quite distinct from those of other microbial rhodopsins (Figures S1A and S4C).

**Figure 5:**
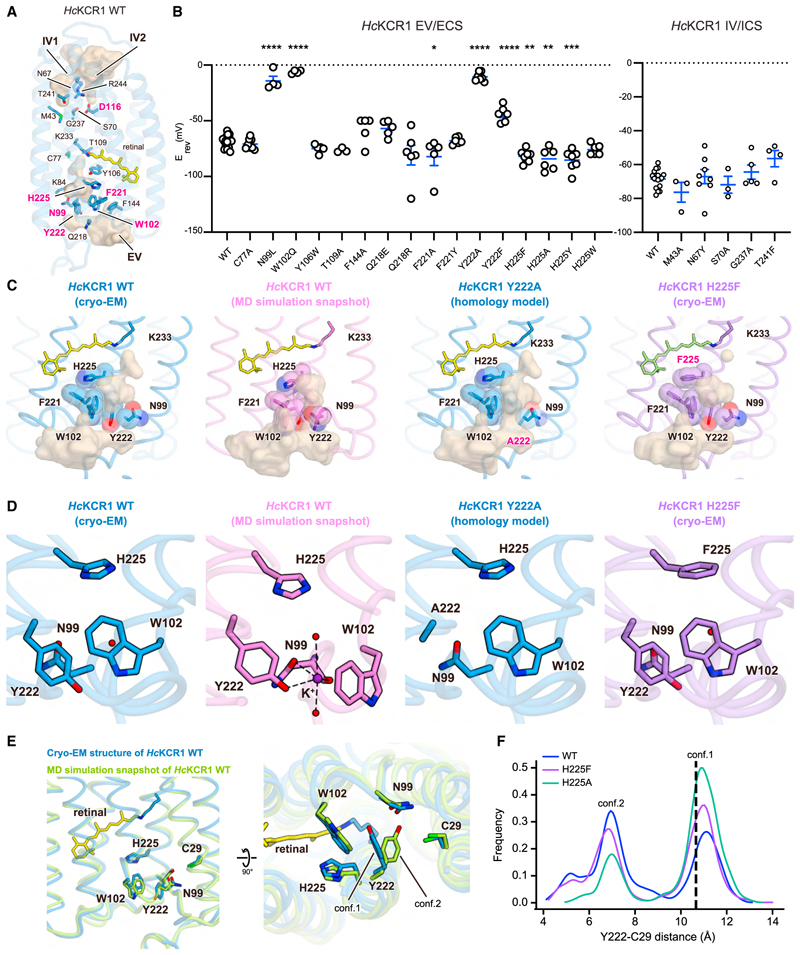
K^+^-selectivity filter (A) Residues along ion-conducting cavities in *Hc*KCR1 WT. E_rev_-affecting mutations are in magenta. (B) E_rev_ summary for mutations highlighted in (A). Mean ± s.e.m. (n = 3-17; one-wayANOVAwith Dunnett’stest; *p < 0.05, **p < 0.01, ***p < 0.001, ****p < 0.0001). (C and D) Selectivity filter region of *Hc*KCR1 WT (cryo-EM structure), K^+^-coordinated form of *Hc*KCR1 WT (MD snapshot), Y222A mutant (homology model), and H225F mutant (cryo-EM structure), viewed parallel to membrane (C) and magnified (D). Cavities calculated with HOLLOW. Black dashes indicate H-bonds; purple and red spheres indicate K^+^ and water, respectively. (E) Superimposed *Hc*KCR1 WT structure (blue) and MD snapshot (green) viewed parallel to membrane (left) and from extracellular side (right). Residues in the filter and retinal are shown as sticks. (F) Histograms of distances between Y222 and C29 during simulations.

To understand the mechanism of K^+^ selectivity, we introduced mutations to residues along the IVs and EVs of *Hc*KCR1 and measured reversal potentials (E_rev_) under physiological ion-gradient conditions (Figures 5A, 5B, S5, S6A, and S6B; STAR Methods). WT *Hc*KCR1 exhibited an E_rev_ of –68.4 ± 1.3 mV and a permeability ratio (*P*_K_/*P*_Na_; STAR Methods) of 25.7 ± 2.7, consistent with previous work^40^ indicating function as a K^+^-selective channel with minor Na^+^ conductance. Most mutations had negligible effects on selectivity, but mutations to N99, W102, D116, F221, Y222, and H225 altered E_rev_ (Figures 5A, 5B, S5B, S6A, and S6B; Data S1). Strikingly, mutants F221A, H225F, H225A, and H225Y showed higher K^+^-selectivity with hyperpolarized E_rev_ (–82.1 ± 8.1, –82.0 ± 1.9, –84.6 ± 4.0, and –85.4 ± 3.5 mV, respectively) (Figure 5B); for possible biological applications, these represent functionally relevant effect-sizes. In contrast, the N99L, W102Q, and Y222A mutants became virtually non-selective to Na^+^/K^+^ with depolarized Erev (–14.25 ± 4.1, –6.25 ± 1.5, and –10.6 ± 1.5 mV, respectively). These five residues are localized in the EV, suggesting that N99, W102, F221, Y222, and H225 might assemble and form a selectivity filter (Figure 5A).

In canonical K^+^ channels, the ion-selectivity filter is typically occupied by K^+^ ions. However, in our KCR structures, although the resolution is high and numerous water densities were observed, no densities were conclusively identified as K^+^ ions (further supported by Attenuated Total Reflection Fourier Transform InfraRed [ATR-FTIR] spectroscopy^59^ [Figures S3G and S3H], UV-Vis absorption spectra measurement [Figure S3I], Fluorescence-detection Size-Exclusion Chromatography-based ThermoStability assay [FSEC-TS]^60^ [Figure S3J], and MicroScale Thermophoresis [MST] experiments^61^ [Figure S3K]). These results collectively validated that K^+^ ions do not stably bind to KCRs in the dark state. We used molecular dynamics (MD) simulations and applied artificial force to pull a K^+^ ion through the extracellular part of the channel (STAR Methods). We found that the ion passes between N99, W102, and Y222, which we refer to as the selectivity triad, and then enters the extracellular vestibule with minimal protein distortion. As the K^+^ ion passes through the triad, it forms a π-cation interaction with W102 and polar interactions with the carbonyl group of N99 and the hydroxyl group of Y222 (Figures 5C and 5D); at this stage. the K^+^ ion is largely dehydrated with just two coordinated water molecules (Figure 5D). These findings are in agreement with our electrophysiology, demonstrating that N99L, W102Q, and Y222F reduce selectivity (Figure 5B). Previous studies have shown that interactions with the face of phenyl rings and carbonyl groups are more favorable for K^+^ than Na^+^ or Li^+^ in a size-dependent manner,^62–64^ providing a rationale for how the selectivity triad favors K^+^.

These findings, along with the homology model of the Y222A mutant, may offer a compelling explanation for differences in E_rev_ between Y222A and Y222F mutations (E_rev_ of Y222A and Y222F are –10.6 ± 1.5 mV and –44.2 ± 2.9 mV, respectively). The Y222A mutation expands the space defined by the selectivity triad, leading to reduction in the energy barrier to partially hydrated ion permeation (Figures 5C and 5D). Consequently, the triad loses ability to function as a K^+^-selective filter (Figure 5B); indeed, the photocurrent amplitude of the Y222A mutant is substantially higher than that of the WT (Figures S5B and S6A). The lower E_rev_ in Y222F compared to Y222A suggests that both the hydroxyl group of Y222 (Figure 5D) and the bulkiness of the side chains comprising the selectivity filter are important for high K^+^ permeability. This finding agrees with the previous results indicating that KCR selectivity is inversely proportional to the size of hydrated substrate cations.^40,65^

In contrast, the reason behind the significant enhancement of K^+^ selectivity we observed in H225 mutations remained unclear, as H225 is not directly involved in the selectivity triad. To address this, we determined the cryo-EM structure of the H225F mutant at 2.7 Å resolution (Figure S2; Table S1) and compared it to WT, initially observing no remarkable differences in the EV (Figures 5C and 5D). While H225 is not part of the selectivity triad, it interacts weakly with W102 and Y222 due to its proximity. Considering that mutations in H225 might affect stability of the selectivity filter, we conducted additional MD simulations for both WT and H225F. In WT *Hc*KCR1, we noticed that the selectivity triad stochastically deviated from the cryo-EM conformation, adopting a more open structure with Y222 rotating away from W102 (Figure 5E; Video S1). We refer to the Y222 conformation similar to that of the cryo-EM structure as conformation 1 (tight) and the Y222 conformation positioned farther from W102 as conformation 2 (loose) (Figures 5E and 5F). WT simulations suggested that when Y222 adopts the loose conformation, the triad architecture becomes significantly opened, transiently reducing channel selectivity (Figures 5B and 5E). Surprisingly, in simulations of the H225F mutant, Y222 occupies the tight conformation for a greater fraction than in WT simulations (Figure 5F; Video S2); simulations of the H225A mutant (also with greater K^+^ selectivity) using our homology model revealed the same trend (Figure 5F; Video S3). These results strongly suggest that the selectivity triad in *Hc*KCR1 has intrinsic flexibility and that mutation to H225 stabilizes the architecture favoring K^+^ selectivity. Intriguingly, the proportional occupancy time in conformation 1 increases in the order WT→ H225F → H225A (Figure 5F), while photocurrent amplitude decreases in the same order (Figures S5B and S6A). This observation is consistent with the idea that conformation 2 represents a state with a lowered energy barrier.

To investigate the impact of H225F on the selectivity triad, we conducted electrophysiology using intracellular K^+^ and extracellular cesium (Cs^+^) or lithium (Li^+^) (Figures S6C and S6D; STAR Methods). Cs^+^ is a weakly hydrated cation with a smaller hydration radius than Na^+^/K^+^, while Li^+^ has a larger hydration radius than both Na^+^ and K^+^.^40,65^ We observed no significant difference in K^+^vs. Cs^+^ permeability between WT and H225F (E_rev_ for WT = −28.7 ± 4.6 mV vs. E_rev_ for H225F = −30.2 ± 2.0 mV; p = 0.7815). However, a pronounced discrepancy was observed in K^+^ vs. Li^+^ permeability between WT and H225F (E_rev_ for WT = −102.8 ± 2.7 mV vs. E_rev_ for H225F = −119.2 ± 2.3 mV; p = 0.0035) (Figures S6C and S6D). These findings further support the idea that the selectivity conferred by the triad and its enhancement by H225F depend upon the hydration radius of cations entering from the extracellular side.

**Figure 6:**
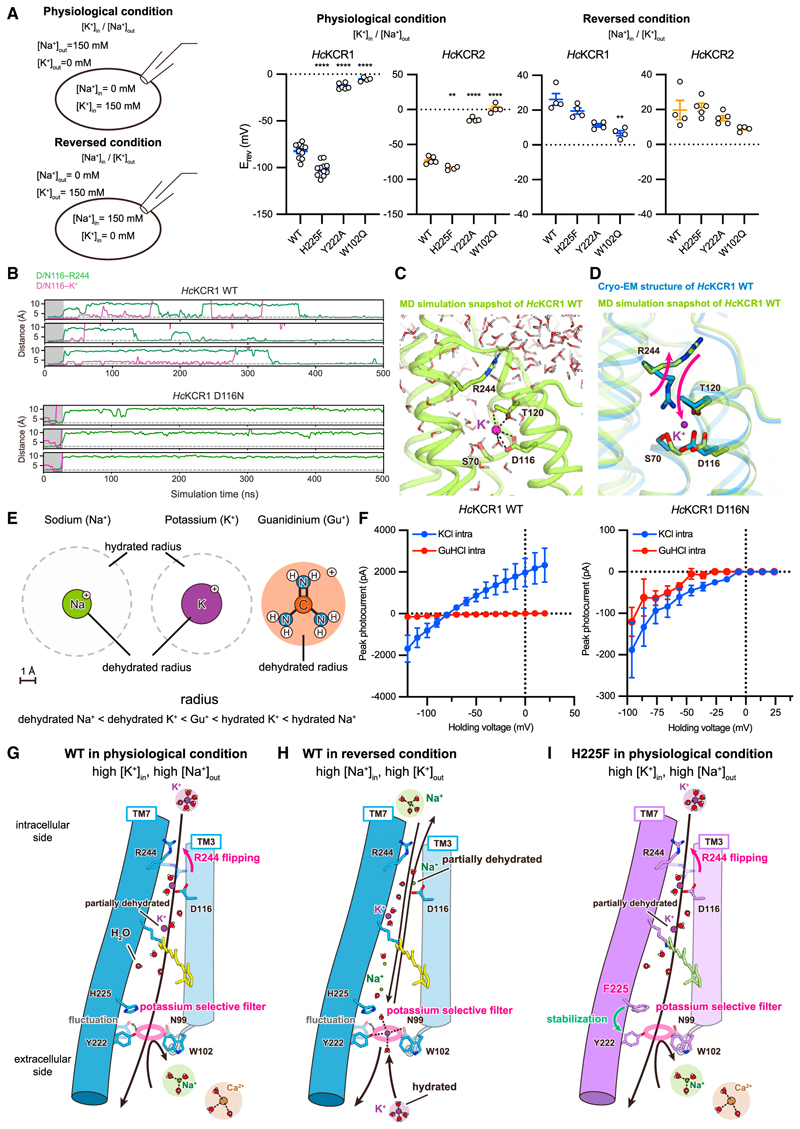
Computational and functional analyses of permeant ion dehydration (A) Schematics (left) and E_rev_ for WT and three mutantsof *Hc*KCR1 and *Hc*KCR2 under physiological (middle) or reversed (right) conditions. Mean ± s.e.m. (n = 4–21; one-way ANOVA with Dunnett’s test; **p < 0.01, ****p < 0.0001). (B) Representative traces from MD simulation of *Hc*KCR1 WT (top) and D116N (bottom). (C) MD snapshot showing transient binding of partially dehydrated K^+^. (D) Superimposed *Hc*KCR1 structure and MD snapshot. (E) Ionic and hydration radii of cations. (F) Current-voltage (*I-V*) relationships of *Hc*KCR1 WT (left) and D116N (right) with and without GuHCl. Mean ± s.e.m. (n = 3–8). (G–I) Ion conduction models of WT *Hc*KCR in physiological (G) and reversed conditions (H), and H225F in physiological conditions (I). TMs 1, 2, and 4–6 are removed forclarity. Black arrow and dashed lines indicate cation flow and H-bonds, respectively. Oxygen and hydrogen atoms ofwater molecules are shown as spheres colored in red and white, respectively. Magenta circles represent K^+^-selective filters.

We performed electrophysiology under two different bi-ionic conditions: one with high extracellular Na^+^ ([Na^+^]_out_, 150 mM NaCl with no K^+^) and high intracellular K^+^ ([K^+^]_in_, 150 mM KCl with no Na^+^) (physiological), and the other with inverted ion concentration gradients (reversed) (Figures 6A and S5A; STAR Methods). In canonical K^+^ channels, specific coordination among water, K^+^ ions, and the backbone carbonyls of the selectivity filter together confer K^+^ selectivity, which is usually maintained regardless of the ion gradient (Figure S6E, right). Similarly, the endolysosomal K^+^/H^+^ channel TMEM175, which employs a different fourfold-symmetric motif as the filter, also maintains selectivity independent of ion gradient.^66^

However, when we measured E_rev_ in *Hc*KCR1, we found that while maintaining robust photocurrents (Figures S6E and S6F), E_rev_ shifted positively (from E_rev_ of −82.2 ± 2.5 mV) but only to 26.2 ± 3.3 mV under the reversed condition, indicating a decrease in *P*K/*P*Na when the concentration gradient is inverted (Figures 6A, middle/right, and S6D, left). This pattern was also observed in *Hc*KCR2 (Figure 6A, middle/right). The hydration state of ions accessing the selectivity filter from intracellular vs. extracellular is likely different since the selectivity filter is on the extracellular side and exposed to the bulk solvent, and ions accessing the filter from the extracellular side are expected to be fully hydrated (Figure 5A). In contrast, the intracellular side of the ion-conducting pathway in KCRs is very narrow (Figure 5A); our MD simulations revealed that the middle of the TM helices would have to move at least 7 Å to allow passage of fully hydrated ions. Given the relatively small structural changes during the photocycle in other microbial rhodopsins,^67–70^ it is unlikely that ions pass through the intracellular pathway while maintaining full hydration, even when the channel is fully open. Indeed, low-temperature FTIR experiments of *Hc*KCR1 in the M intermediate (open state) revealed structural change not different from that of *Hs*BR, insufficient for conduction of fully hydrated ions (manuscript in preparation).^68,71^

In our MD simulations of *Hc*KCR1 in the presence of K^+^, we observed transient binding of K^+^ near the IV, defined by the constriction at D116 and T120 (Figures 6B–6D). These events were accompanied by partial dehydration of K^+^ (Figure 6C), and the salt bridge between R244 and D116 was lost, with the R224 side chain reoriented toward solvent and K^+^ replacing the guanidinium of R224, making simultaneous binding unfavorable (Figures 6B and 6D). Considering that D116 is located near the intracellular opening, the transient binding of K^+^ in our simulations may reveal partial dehydration of ions entering from the intracellular side. Computational and functional analyses of the D116N mutant were in good agreement with this hypothesis. MD simulation revealed that K^+^ does not bind to D116N and T120 in the D116N mutant, unlike in WT *Hc*KCR1 (Figure 6B). Electrophysiology showed that while currents were nearly abolished in D116N, a small inward current remained, revealing that D116 is not absolutely required for ion conduction (Figure S5B).

We further investigated the importance of dehydration by analyzing effects of guanidinium ions (Gu^+^) on *Hc*KCR1 activity (Figures 6E and 6F; STAR Methods). Gu^+^ has a radius larger than that of dehydrated K^+^ or Na^+^ but smaller than that of hydrated K^+^ or Na^+^ (Figure 6E); moreover, Gu^+^ is one of the most weakly hydrated cations.^72^ Addition of intracellular Gu^+^ completely blocked channel activity of WT *Hc*KCR1 (Figure 6F). The lack of outward Gu^+^ current in itself indicated that either Gu^+^ acts as a pore blocker by interacting with a specific binding site in the pore or that Gu^+^ is too large for *Hc*KCR1 to transport (in which case even larger cations—such as fully hydrated K^+^ or Na^+^—would also be too large, suggesting that partial dehydration may be important for ion transport). We next found that Gu^+^ indeed blocked both inward and outward ion conductance, i.e., the transport of Na^+^ and K^+^ (Figure 6F, left). Considering the structural similarity between Gu^+^ and the side chain of the arginine, it is possible that D116 serves as a Gu^+^ binding site as R244 interacts with D116 and that Gu^+^ binding to this site (D116) prevents ion flux. This idea was supported by further electrophysiology of the D116N mutant showing that Gu^+^ does not significantly inhibit the inward current remaining in the mutant (Figure 6F, right), presumably no longer able to bind to D116 just as with the R244 guanidinium moiety.

### Proposed mechanism for the high K^+^ permeability ratios of KCRs

Regarding the mechanism for high K^+^ permeability ratios of KCRs, we note that under physiological conditions, the concentrations of Na^+^, Ca^2+^, and Mg^2+^ are higher on the extracellular side, while the concentration of K^+^ is higher on the intracellular side. When the KCR is opened by light, fully hydrated Na^+^ approaching from the extracellular side would encounter a large energy barrier at the selectivity filter, mainly formed by the triad N99, W102, and Y222. A K^+^ ion at the triad can form a π-cation interaction with the indole ring of W102 (Figure 5D), which is more favorable^62–64^ for K^+^ than Na^+^, thus providing a K^+^-specific energy barrier reduction. It is likely that the interaction energy between hydrated Na^+^ and the triad is insufficient to compensate for the energy barrier (including the dehydration penalty) and that the Na^+^ ions that might enter from the extracellular side would be outcompeted by (1) K^+^ ions from the intracellular side and, if present, (2) hydrated K^+^ ions from the extracellular side. Hydrated Ca^2+^ and Mg^2+^, with even larger sizes, energy barriers, and dehydration energies, would be blocked accordingly (Figure 6G). Of note, monovalent cations from the intracellular side may experience gradual dehydration (possibly initiated by D116 interaction), reducing the number of waters as the selectivity triad is reached. Regardless of ion flux direction, interaction between partially dehydrated K^+^ and the triad is more effective at reducing the energy barrier, accounting for selective K^+^ permeation (Figure 6G).

When the gradient is inverted, Na^+^ from the intracellular side becomes the dominant ion undergoing partial dehydration before reaching the filter. Thus, the free energy provided by the gradient can compensate for sub-optimal Na^+^-triad interactions (Figure 6H). Notably, the selectivity triad architecture is dynamic (Video S1). When Y222 moves away from W102, the structure deforms and the energy barrier for ions is reduced, resulting in a transient decrease in selectivity. The mutations in residues surrounding the filter (e.g., F221 or H225) can enhance K^+^selectivity by stabilizing the filter region (Figure 6I).

In conclusion, KCRs adopt a distinctive mechanism to specifically favor K^+^flux, operating under physiological ion balance conditions. This mechanism relies on three key factors, including (1) the extracellular selectivity filter, (2) the intracellular dehydration system, and (3) the high extracellular Na^+^ and intracellular K^+^ present under physiological conditions. The importance of multiple factors is supported by electrophysiology with filter mutants (Figure 6A, middle/right); even under bi-ionic (Na^+^ and K^+^) conditions with inverted gradients, WT KCRs maintain weak K^+^ selectivity (E_rev_ for *Hc*KCR1 = 26.2 ± 3.3 mV; *Hc*KCR2 =19.8 ± 5.6 mV). However, disruptions in the selectivity triad lower the energy barrier for ion passage, causing KCRs to function more as non-selective cation channels under reversed-ion conditions (E_rev_ for *Hc*KCR1 Y222A = 11.3 ± 1.0 mV, W102Q = 6.7 ± 1.5 mV; *Hc*KCR2 Y222A =15 ± 1.4 mV, W102Q = 9.0 ± 0.7mV). These findings suggest that both the concentration gradient and selectivity filter are critical for proper functioning of KCRs as K^+^ channels.

### *In vitro* and *in vivo* applications of the KCR variants with enhanced K^+^ selectivity

**Figure 7:**
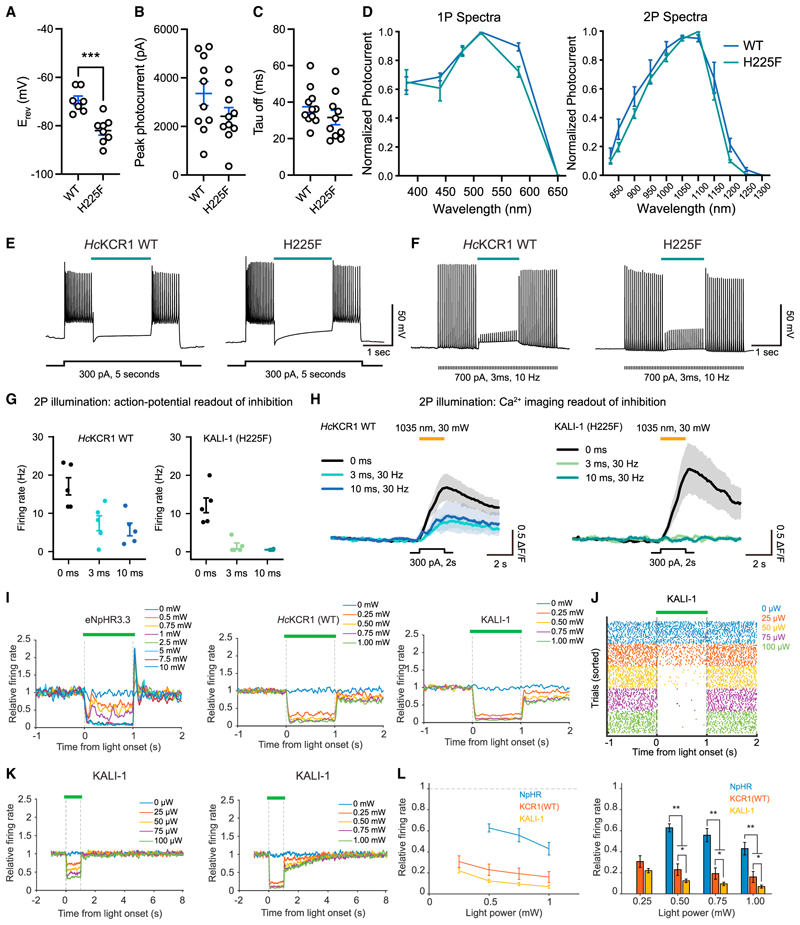
*In vitro* and *in vivo* applications of KALI-1 (A–C) Summary of reversal potential (A), peak photocurrent (B), and deactivation kinetics (C) (mean ± s.e.m.; n = 7–8; two-tailed t test; ***p < 0.001). (D) Summary of action spectra for WT and H225F variant under 1P (left) and 2P (right) illumination (mean ± s.e.m.; n = 5–6). (E and F) Optogenetic inhibition in brain slices stimulated by tonic (E) or phasic (F) currents. (G) Summary plots of optogenetic inhibition for different 2P durations in cultured neurons (mean ± s.e.m.; n = 5). (H) Representative Ca^2+^ transients during optogenetic inhibition with different 2P illumination durations in neurons (mean ± s.e.m.; n = 5). (I) Neuropixels in mouse retrosplenial cortex (RSP). Data represent n = single units: 19 for eNpHR3.3 (left), 78 for WT (middle), and 241 for KALI-1 (right). (J) Spike rasters for exemplar single units inhibited by KALI-1 in moderate/low power. (K) Relative firing rate ofthe inhibited neuronal population in KALI-1 mice in low (left) and high (right) power. Datafrom 102 unitsfor moderate/low powerand 139 for high power. (L) Summary of eNpHR3.3,WT, and KALI-1 data in (I) represented as both line (left) and bargraphs(right). Mean ± s.e.m. acrosssingle units(bootstrap); two-tailed ttest; *p < 0.05, **p < 0.01.

KCRs hold potential as potent inhibitory optogenetic tools for neuroscience. We therefore aimed to design improved inhibitory optogenetic tools for *in vitro* and *in vivo* applications, leveraging our structural understanding of enhanced K^+^ selectivity. The H225F mutant exhibited hyperpolarized E_rev_ compared to WT (Figure 7A; -69.6 ± 1.9 mV vs. -82.0 ± 1.9 mV; p = 0.0004) with only slightly decreased photocurrents (Figure 7B) and comparable kinetics (Figure 7C) in cultured neurons. Intriguingly, the action spectrum of the H225F demonstrated reduced blue- and red-shoulders under both one-photon (1P) and two-photon (2P) illumination (Figure 7D). In slice physiology experiments, we observed efficient inhibition of spiking (Figures 7E and 7F; STAR Methods), consistent with our data from HEK cells (Figure 5B) and cultured neurons (Figures 7A–7D). Because of these potent loss-of-function properties, we name the H225F mutants of *Hc*KCR1 and 2 as KALI-1 and KALI-2 (K^+^-selectivity Augmented Light-Gated Ion Channels).

We initially tested KALI properties for inhibitory optogenetics in all-optical physiology. We began evaluation with 1P or 2P illumination in neurons expressing KALI-1 or KALI-2. The blue-shifted KALI-2 performed exceptionally in 1P all-optical experiments with red Ca^2+^ indicators XCaMP-R and sRGECO (Figures S7A–S7G), and, unlike eNpHR3.3, showed no rebound activity upon light termination (Figures S7E and S7F). Moreover, KALI-1 displayed low optical crosstalk (compared with WT) in 2P experiments, highlighting utility with green indicators like GCaMP6m (Figures S7H–S7M), and improved 2P inhibition of evoked Ca^2+^ transients across various stimulation pulse widths (Figures 7G and 7H).

We also tested KALIs with electrophysiological recording *in vivo* using Neuropixels probes.^73^ Both WT *Hc*KCR1 and KALI-1 showed robust expression in mouse retrosplenial cortex (Figure S7N). Consistent with our 1P all-optical experiments (Figures S7A–S7G), KALI-1 inhibited spontaneous spiking more efficiently than WT *Hc*KCR1 or the Cl^−^ pump eNpHR3.3 (Figures 7I–7L), and unlike eNpHR3.3, KALI-1 showed no rebound activity after light cessation (Figure 7I). Although exceptionally potent inhibition could sometimes outlast the light intervention by a few seconds, recovery was completed within 3 s after light-off, which is suitable for fast optogenetic behavioral design and interpretation. In cases where even a few seconds of persistent circuit response might be undesirable, reducing light power allowed for instantaneous recovery after light-off (Figures 7K and 7L). In summary, KALIs provide substantial advantages for inhibitory optogenetics in diverse experimental setups, including two of the most powerful modern approaches: all-optical physiology of intact neural circuitry and high-density recording of neural activity *in vivo*.

## Discussion

KCRs have attracted attention for K^+^ selectivity and spectral properties, which in combination with the strong photocurrents and high light sensitivity of the broader PLCR class^34,40^ together suggest exceptional utility for optogenetic silencing. However, even at a basic level, these properties of KCRs had not been understood—including how K^+^ selectivity can be achieved in an asymmetric channel pore—with significance for both fundamental science and biological applications. Here, we have revealed the initial structural and mechanistic underpinnings of these special properties, obtained insights into the emergence of K^+^ selectivity in microbial opsins, and provided structure-guided modifications offering opportunities for neuroscience.

### Structural principles underlying large spectral shifts

The *Hc*KCR1 and *Hc*KCR2 findings revealed here represent the second and third PLCR family members with high-resolution structures, enabling structural comparison among PLCRs. Many unusual features observed in the structure of ChRmine, the initial structure from the PLCR family,^38,46^ are conserved in *Hc*KCR1 and *Hc*KCR2, such as trimeric assembly, short TM3, deformed Schiff base, and large cavities within the monomer (Figures 1, 2, 3, and 4). However, major differences were also noted, including variations in primary counterion and water distribution in the Schiff base region, potentially related to different retinal isomer composition after light absorption. In ChRmine, as with other microbial rhodopsins, all-*trans*-retinal is isomerized chiefly to 13-*cis* upon light illumination,^38^ while in *Hc*KCR1, the percentages of increased 9-, 11-, and 13-*cis* are similar (Figures S3A and S3B). The 13-*cis* retinal-bound *Hc*KCR1 likely corresponds to the open state; further studies will be needed to identify function of the 9- and 11-*cis* photoproducts.

Retinal isomer composition is furthermore different between the *Hc*KCRs in the dark. Unlike most ChRs, *Hc*KCR2 exhibits a 6-*s*-*cis*-retinal (Figure 3E). Two residues (T136/G140 in *Hc*KCR1 and A136/A140 in *Hc*KCR2) are involved in the conversion, and considering a previous study with the C1C2 chimera,^56^ G-to-A-replacement at position 140 could be sufficient for the conversion, while position 136 may fine-tune the twist. Interestingly, while G140 of *Hc*KCR1 is conserved (Figure S1A), blue-shifted ChRs, including *Ps*ChR, *Ts*ChR, *KnChR*, WiChR, and *B1*ChR2, have alanine at this position.^74,75–77^ This “non-G rule” could prove useful in searching for blue-shifted rhodopsins.

### Structural foundations of diversity in selectivity and kinetics

The ECS interaction network is the main difference between *Hc*KCRs and ChRmine. Due to displacements of ECL1 and TM7 (Figure 1F), three residues (N99, W102, Y222) create the selectivity filter, while F/Y221 and H225 stabilize the tight conformation of this architecture in *Hc*KCRs (Figure 4C). However, even between *Hc*KCR1 and *Hc*KCR2, the interaction network differs (Figures 4C and S2O). Mutations at positions D/N18, L/Q211, Q/R218, and F/Y221 alter the ECS (Figure S1A), possibly contributing to the different P_K_/P_Na_ of *Hc*KCR1 (25.7 ± 2.7) and *Hc*KCR2 (17.0 ± 1.5).

A recently discovered KCR, WiChR, exhibits a higher K^+^ permeability ratio and different amino acids at positions 18, 210, 211, 218, 221, and 222 (*Hc*KCR1 numbering), suggesting that the ECS interaction network is also different in WiChR and that structural information of WiChR would be valuable in understanding the differences in K^+^ selectivity among KCRs. Further detailed comparisons and insights into filter mechanisms could lead to the development of novel KCRs with improved K^+^ selectivity, given that we have already successfully created *Hc*KCR mutants with *P*_K_/*P*_Na_ values > 50 (e.g., H225F) guided by information from our *Hc*KCR structures (Figure 5B).

In addition to H225F, we discovered two additional mutations that usefully modulate the properties of *Hc*KCRs: C110T for long-timescale and light-sensitive effects and Y222A for excitatory effects (Figures 3C, 3D, 5B, S5B, and S6A). The C110T mutation remarkably increased the τ_off_ of *Hc*KCRs by over 1500-fold, a surprising result as previous attempts with other PLCRs had failed. The C110T *Hc*KCR variants provide improved capability for prolonged control over neuronal activity at even lower light levels^4,5^; indeed, the transferability of this mutation^55^ may enable more long-τ_off_ mutants based on PLCRs. The Y222A mutation, in contrast, does not significantly affect τ_off_ but depolarizes E_rev_ by ~70 mV, thereby transforming function from inhibitory to excitatory under physiological conditions. The photocurrent amplitude of this mutant is comparable to or higher than that of WT *Hc*KCR (Figures S5B and S6A), giving rise to a particularly potent excitatory optogenetic tool.

### Perspectives on evolution and structure-guided design of K^+^ selectivity

Until the present study, all structurally resolved K^+^ channels showed a common tetramer-type assembly for the K^+^ selectivity filter^78^ (Figures S1B–S1D). Even the lysosomal ion channel TMEM175, initially reported to be K^+^-selective^29,66,79^ but shown to be more H^+^-selective under physiological conditions,^80^ lacks the canonical K^+^-selectivity motif but shares the tetrameric assembly pattern. Interestingly, while microbial rhodopsins are known to form diverse oligomeric assemblies, not a single rhodopsin forming a tetramer has yet been reported.^3^ This observation suggests that tetrameric assembly may be unstable for microbial rhodopsins, which achieve pore selectivity within each monomer. The orientation of the conserved arginine residue on TM3/ECL1 (R82 in *Hs*BR and R112 in ChRmine) may be important to define channel- vs. pump-type properties, and indeed, replacement of this arginine (R→Q) significantly affected function and ion selectivity of the Na^+^ pump KR2 (Figure S4C).^38,81^ In KCRs, this arginine is replaced by tryptophan, and this replacement may be an important step in evolving high K^+^ permeability ratios.

To fully understand the KCR photocycle, further studies including structural determination of intermediate states in *Hc*KCRs will be necessary. In addition, while KCRs serve as potent inhibitory tools, there is room for improvement. For example, the unitary conductance of *Hc*KCR1 is ~0.7 pS,^40^ still considerably lower than for canonical K^+^ channels (e.g., the unitary conductance of Kv1.1 and Kv1.2 are 8.7–20 and 14–18 pS, respectively^82^). However, the insights presented here already reveal key mechanisms underlying high K^+^ permeability ratios in KCRs and have enabled molecular engineering of diverse KCR variants with improved functionality, including C110T, Y222A, and H225F.

These findings provide insight into the evolution of solutions to the ancient problem of K^+^-selectivity and help advance the discovery and application of opsins with distinct ion selectivity. These natural microbial rhodopsins, together with the variants developed in this study, will further diversify and improve optogenetic technologies, creating avenues for basic life science and biomedical research.

### Limitations of the study

The structures obtained here represent the dark, non-conducting state of KCRs, making it challenging to determine the exact ion-conducting pathway in the open state. Currently, no open-state structures for any channelrhodopsin have been reported, posing difficulties in constructing accurate homology models. Furthermore, due to the relatively slow channel opening in KCRs (as indicated in Figure 2F), employing MD simulations to calculate the open state is not feasible. Slowly inactivating mutants, such as the C110T variant we described here and in the initial KCR structure discovery report (Tajima et al., 2022),^83^ might be beneficial for determining the open-state structure, but even with these variants, it will be essential to achieve maximal open-state occupancy with light irradiation. By utilizing such mutants under suitable conditions or by employing techniques like time-resolved serial femtosecond crystallography (TR-SFX) or time-resolved cryo-EM, we might gain insights into the open-state structure of KCRs.^36^

Compared to WT KCRs, KALIs exhibit higher K^+^ selectivity (Figures 5B and 7A) and reduced optical crosstalk when combined with green Ca^2+^ indicators (Figures 7G and 7H). But while our MD simulations provided insight into the structural basis for enhanced K^+^ selectivity (Figures 5C–5F), the finding of reduced optical crosstalk was largely serendipitous and remains unexplored mechanistically; the difference in action spectra between WT and KALI may contribute (Figure 7D) but seems unlikely to serve as a complete explanation. Further studies, including detailed spectroscopic analyses of intermediates in the photocycle, will elucidate the precise reasons for this improvement.

## Star⋆Methods

### Key Resources Table

**Table:** 

REAGENT or RESOURCE	SOURCE	IDENTIFIER
Bacterial and virus strains		
One Shot Stbl3 Chemically Competent *E. coli*	Thermo Fisher Scientific	Cat# C737303
*E. coli* BL21 (DE3)	Thermo Fisher Scientific	Cat# EC0114
*E. coli* C41 (DE3)	Sigma-Aldrich	Cat# CMC0017
*E. coli* Mach1	Thermo Fisher Scientific	Cat# C862003
*E. coli* DH10Bac	Thermo Fisher Scientific	Cat# 10361012
Adeno-Associated Virus Coat Protein 2/8	Constructed in the Deisseroth lab; packaged at the Stanford Gene Vector and Virus Core (GVVC)	AAV8
AAV8-CaMKIIa-*Hc*KCR1 (WT)-EYFP	Constructed in the Deisseroth lab; packaged at the GVVC	N/A
AAV8-CaMKIIa-*Hc*KCR1 (H225F)-EYFP	Constructed in the Deisseroth lab; packaged at the GVVC	N/A
AAV8-CaMKIIa-*Hc*KCR2 (WT)-EYFP	Constructed in the Deisseroth lab; packaged at the GVVC	N/A
AAV8-CaMKIIa-*Hc*KCR2 (H225F)-EYFP	Constructed in the Deisseroth lab; packaged at the GVVC	N/A
AAV8-CaMKIIα-XCaMP-R	Constructed in the Deisseroth lab; packaged at the GVVC	N/A
AAV8-CaMKIIα-sRGECO	Constructed in the Deisseroth lab; packaged at the GVVC	N/A
AAV8-CaMKIIα-NpHR3.3-mCherry	Constructed in the Deisseroth lab; packaged at the GVVC	N/A
AAV8-CaMKIIα-GCaMP6m	Constructed in the Deisseroth lab; packaged at the GVVC	N/A
Chemicals, peptides, and recombinant proteins		
APV	Tocris	Cat# 0106
CNQX	Tocris	Cat# 0190
Tetrodotoxin	Tocris	Cat# 1078
Fluorodeoxyuridine	Sigma	Cat# F0503
Lipofectamine 2000	Thermo Fisher Scientific	Cat# 11668027
FuGENE	Promega	Cat# E2311
Benzamidine	Nacalai tesque	Cat# M9G4533
Leupeptin	Peptide Institute	Cat# 4041
*n*-Dodecyl-β-D-maltoside (DDM)	EMD Millipore	Cat# 324355
*n*-Dodecyl-β-D-maltoside (DDM)	EMD Millipore	Cat# D97002-C
Ni-NTA Superflow	QIAGEN	Cat# 30430
Superdex 200 Increase 10/300 GL	Cytiva	Cat# 28990944
TSKgel3000SWXL column	TOSOH bioscience	Cat# 0008541
All-*trans*-retinal (ATR)	Sigma-Aldrich	Cat# R2500
StockOptions pH buffer kit	Hampton Research	Cat# HR2-241
ESF 921 Insect Cell Culture Medium, Protein Free	Expression systems	Cat# 96-001-01
Monolith His-Tag Labeling Kit RED-tris-NTA 2^ND^ Generation	NanoTemper Technologies	Cat# MO-L018
Deposited data		
*Hc*KCR1 – atomic model	From this paper	PDB: 8H86
*Hc*KCR2 – atomic model	From this paper	PDB: 8H87
*Hc*KCR1 H225F mutant– atomic model	From this paper	PDB: 8IU0
*Hc*KCR1 – Cryo-EM map	From this paper	EMDB-34530
*Hc*KCR2 – Cryo-EM map	From this paper	EMDB-34531
*Hc*KCR1 H225F mutant – Cryo-EM map	From this paper	EMDB-35713
*Hc*KCR1 – Cryo-EM movie frames	From this paper	EMPIAR-11558
*Hc*KCR2 – Cryo-EM movie frames	From this paper	EMPIAR-11558
*Hc*KCR1 H225F mutant – Cryo-EM movie frames	From this paper	EMPIAR-11558
Experimental models: Cell lines		
*Spodoptera frugiperda* Sf9 cells	Expression systems	Cat# 94-001F
HEK293T cells	ATCC	Cat# CRL-3216
Primary rat hippocampal cultured neuron	This paper	N/A
Experimental models: Organisms/strains		
C57BL/6J	Jackson	000664
Sprague-Dawley rat pups	Charles River	N/A
Recombinant DNA		
pFastBac-*Hc*KCR1 (WT)-EGFP-His10	This paper	N/A
pFastBac-*Hc*KCR1 (WT) -His10	This paper	N/A
pFastBac-*Hc*KCR1 (D105N)-EGFP-His10	This paper	N/A
pFastBac-*Hc*KCR1 (D229N)-EGFP-His10	This paper	N/A
pFastBac-*Hc*KCR1 (H225F)-EGFP-His10	This paper	N/A
pFastBac-*Hc*KCR1 (T136A/G140A)-EGFP-His10	This paper	N/A
pFastBac-*Hc*KCR2 (WT)-EGFP-His8	This paper	N/A
pFastBac-*Hc*KCR2 (D105N)-EGFP-His8	This paper	N/A
pFastBac-*Hc*KCR2 (D229N)-EGFP-His8	This paper	N/A
pFastBac-*Hc*KCR2 (A136T/A140G)-EGFP-His8	This paper	N/A
pET-43a(+)-MSP1E3D1	This paper	N/A
pcDNA 3.1-*Hc*KCR1 (WT)-eYFP	This paper	N/A
pcDNA 3.1-*Hc*KCR2 (WT)-eYFP	This paper	N/A
pET-43a(+)-KR2	This paper	N/A
pAAV-*Hc*KCR1 (WT)-EYFP	This paper	N/A
pAAV-*Hc*KCR1 (H225F)-EYFP	This paper	N/A
Software and algorithms		
Serial EM software	Mastronarde^84^	https://bio3d.colorado.edu/SerialEM/
MotionCor2	Zheng et al.^85^	https://emcore.ucsf.edu/ucsf-software
RELION 4.0	Zivanov et al.^86^	https://github.com/3dem/relion
UCSF Chimera	Pettersen et al.^87^	https://www.cgl.ucsf.edu/chimera/
Chimera X	Goddard et al.^88^	https://www.rbvi.ucsf.edu/chimerax/
Coot	Emsley and Cowtan^89^	https://www2.mrc-lmb.cam.ac.uk/personal/pemsley/coot/
Cuemol2	N/A	http://www.cuemol.org/ja/index.php?cuemol2
Servalcat	Yamashita et al.^90^	https://github.com/keitaroyam/servalcat
GraphPad Prism	GraphPad	Graphpad.com
MATLAB	Mathworks	Mathworks.com
pClamp 10.6	Molecular Devices	https://www.moleculardevices.com
Other		
Optic fiber implants	Doric lenses	Ordering Code: MFC_400/430-0.66_2.5mm_MF2.5_FLT
MultiClamp700B Amplifier	Molecular Devices	https://www.moleculardevices.com
DigiData 1440A	Molecular Devices	https://www.moleculardevices.com
DM-LFSA	Leica	N/A
Digital Optical Power and Energy Meter	Thorlabs	PM100D
SPECTRA-X Light Engine	Lumencor	https://lumencor.com
R 1.2/1.3 grid: Au 300 mesh	QUANTIFOIL	Cat# N1-C14nAu30-01
AMICON MWCO 10,000	Merck/Millipore	Cat# UFC801024
AMICON MWCO 50,000	Merck/Millipore	Cat# UFC805024
AKTA pure 25 L1	Cytiva	Cat# 29018225
Fluorescence detection HPLC system	Shimadzu	Model Prominence
V-750 UV-Visible Spectrophotometer	JASCO	https://jascoinc.com/products/spectroscopy/
Vitrobot Mark IV	FEI/Thermo Fisher Scientific	https://www.thermofisher.com/jp/ja/home/electron-microscopy/products/sample-preparation-equipment-em/vitrobot-system.html
300 kV Titan Krios microscope	FEI/Thermo Fisher Scientific	Out of production; https://www.thermofisher.com/jp/ja/home/electron-microscopy/products/transmission-electron-microscopes.html
K3 Summit camera with post-column energy filter	Gatan/Quantum	https://www.gatan.com/K3
Innova S44i R	Eppendorf	Cat# S44I010226
NanoTemper Monolith	NanoTemper Technologies	https://nanotempertech.com/monolith/

### Resource Availability

#### Lead contact

Further information and requests for resources and reagents should be directed to and will be fulfilled by the Lead Contact, Karl Deisseroth (deissero@stanford.edu).

#### Materials availability

Plasmids and viruses detailed in this manuscript are freely available for academic use upon request to the corresponding authors.

### Experimental Model and Study Participant Details

#### Insect cell culture

*Spodoptera frugiperda* (Sf9) cells (Expression systems, authenticated by the vendor) were cultured in ESF921 medium (Expression systems) at 27.5°C with 130 rpm in an Innova S44i R shaking incubator (Eppendorf).

#### Primary neuron and HEK293 cell culture

Primary cultured hippocampal neurons were obtained from P0 Sprague-Dawley rat pups (Charles River) and isolated from CA1 and CA3 regions. The tissues were digested with 0.4 mg/mL papain (Worthington) and seeded onto glass coverslips coated with 1:30 Matrigel (Becton Dickinson Labware). The cultures were maintained in a 5% CO2 humid incubator using Neurobasal-A media (Thermo Fisher) supplemented with 1.25% FBS (HyClone), 4% B-27 supplement (Gibco), 2mM Glutamax (Gibco), and 2 mg/mL fluorodeoxyuridine (FUDR, Sigma). Neurons were grown on coverslips in a 24-well plate at a density of 65,000 cells per well. HEK293FT cells (Thermo Fisher, authenticated by the vendor) were maintained in a 5% CO_2_ humid incubator with DMEM media (GIBCO) supplemented with 10% FBS (Invitrogen), and 1% Penicillin-Streptomycin (Invitrogen), and were enzymatically passaged at 90% confluence by trypsinization.

#### Animals used for surgeries

All mouse experiments conformed to guidelines established by the National Institutes of Health and were conducted under protocols approved by the Stanford Administrative Panel on Laboratory Animal Care. All mice were group-housed in a light-regulated colony room (lights on at 07:00, off at 19:00), with food and water available *ad libitum*. Both male and female mice 4–20 weeks of age were used for all studies.

### Method Details

#### Cloning, protein expression, and purification of *Hc*KCR1 WT, *Hc*KCR2 WT, and *Hc*KCR1 H225F mutant

Wild-type *Hc*KCR1 (M1-S265) was modified to include an N-terminal influenza hemagglutinin (HA) signal sequence and FLAG tag epitope, and C-terminal enhanced green fluorescent protein (eGFP), followed by 10 × histidine (His) and Rho1D4 epitope tags; the N-terminal and C-terminal tags are removable by human rhinovirus 3C (HRV3C) protease cleavage. Wild-type *Hc*KCR2 (M1-D265) was modified to include C-terminal Kir2.1 membrane targeting sequence, HRV3C protease cleavage sequence, eGFP, and 8 × His tag. *Hc*KCR1 H225F mutant was designed by QuikChange site-directed mutagenesis method based on the *Hc*KCR1 WT construct.

The constructs were expressed in *Spodoptera frugiperda* (Sf9) insect cells using the pFastBac baculovirus system. Sf9 insect cells were grown in suspension to a density of 3.0 × 10^6^ cells mL^−1^, infected with baculovirus and shaken at 27.5°C for 24 h. All-*trans* retinal (ATR) (Sigma-Aldrich) is supplemented to a final concentration of 10 μM in the culture medium 24 h after the infection. The cell pellets were lysed with a hypotonic lysis buffer (20 mMHEPES-NaOH pH 7.5, 20 mM NaCl, 10 mM MgCl_2_,1 mM benzamidine, 1 μg mL^−1^ leupeptin, 10 μM ATR), and cell pellets were collected by centrifugation at 10,000 ×g for 30 min. The above process was repeated twice; then, cell pellets were disrupted by homogenizing with a glass Dounce homogenizer in a hypertonic lysis buffer (20 mMHEPES-NaOH pH 7.5, 1 M NaCl, 10 mM MgCl_2_, 1 mM benzamidine, 1 μg mL^−1^ leupeptin, 10 μM ATR), and crude membrane fraction was collected by ultracentrifugation (45Ti rotor, 125,000 ×g for 1 h). The above process was repeated twice; then, the membrane fraction was homogenized with a glass douncer in a membrane storage buffer (20 mMHEPES-NaOH pH 7.5, 500 mM NaCl, 10 mM imidazole, 20% glycerol, 1 mM benzamidine, 1 μg mL^−1^ leupeptin), flash frozen in liquid nitrogen, and stored at −80°C until use.

The membrane fraction was solubilized in a solubilization buffer (1% *n*-dodecyl-β-D-maltoside (DDM) (EMD Millipore), 20 mMHEPES-NaOH pH 7.5, 500 mM NaCl, 20% glycerol, 5 mM imidazole, 1 mM benzamidine, 1 μg mL^−1^ leupeptin) and solubilized by stirring at 4°C for 2 h. The insoluble cell debris was removed by ultracentrifugation (45Ti rotor, 125,000 ×g, 1 h), and the supernatant was mixed with the Ni-NTA superflow resin (QIAGEN) at 4°C for 2 h. The Ni-NTA resin was loaded onto an open chromatography column, washed with 2.5 column volumes of wash buffer (0.05% DDM, 20 mM HEPES-NaOH pH7.5,100 mM NaCl, and 25 mM imidazole) three times, and eluted by elution buffer (0.05% DDM, 20 mMHEPES-NaOH pH7.5, 100 mM NaCl, and 300 mM imidazole). After tag cleavage by His-tagged 3C protease, the sample was reapplied onto the Ni-NTA open column to trap the cleaved eGFP-His-tag and His-tagged 3C protease. The flow-through fraction was collected and concentrated to approximately 2 mg mL^−1^ using an Amicon ultra 50 kDa molecular weight cutoff centrifugal filter unit (Merck Millipore). The concentrated samples were ultracentrifuged (TLA 55 rotor, 71,680 ×g for 30 min) before size-exclusion chromatography on a Superdex 200 Increase 10/300 GL column (Cytiva), equilibrated in DDM SEC buffer (0.03% DDM, 20 mMHEPES-NaOH pH 7.5, 100 mM NaCl). The peak fractions of the protein were collected and concentrated to approximately 10 mg mL^−1^. For *Hc*KCR1 H225F mutant, sodium was replaced with potassium for all steps of the purification.

#### Preparation of membrane scaffold protein

Membrane scaffold protein (MSP1E3D1) is expressed and purified as described earlier^91^ with the following modifications. Briefly, MSP1E3D1 gene in pET-43a(+) was transformed in *Escherichia coli (E. coli)* BL21 (DE3) cells. Cells were grown at 37°C with shaking to an OD_600_ of 0.5-1.0, and then expression of MSP1E3D1 was induced by addition of 1 mM IPTG. Cells were further grown for at 37°C for 4 h, and cells were harvested by centrifugation. Cell pellets were resuspended in PBS (−) buffer supplemented with 1% Triton X-100 and protease inhibitors and were lysed by sonication. The lysate was centrifuged at 30,000×*g* for 30 min, and the supernatant was loaded onto a Ni-NTA column equilibrated with lysis buffer, followed by washing with 4 column volumes of wash buffer-1 (40 mMHEPES-NaOH pH 7.5, 300 mM NaCl, 1% Triton X-100), 4 column volumes of wash buffer-2 (40 mMHEPES-NaOH pH 7.5, 300 mM NaCl, 50 mM sodium cholate), 4 column volumes of wash buffer-3 (40 mMHEPES-NaOH pH 7.5, 300 mM NaCl), 4 column volumes of wash buffer-4 (40 mMHEPES-NaOH pH 7.5, 300 mM NaCl, 20 mMimidazole), and eluted with wash buffer-4 containing 300 mM imidazole. The eluted MSP1E3D1 was dialyzed in buffer containing 10 mMHEPES-NaOH pH 7.5,100 mM NaCl, and concentrated to approximately 10 mg mL^−1^ using an Amicon ultra 10 kDa molecular weight cutoff centrifugal filter unit (Merck Millipore). The concentrated samples were ultracentrifuged (TLA 55 rotor, 71,680 ×g for 30 min), and stored at −80°C after flash freezing in liquid nitrogen. The concentration was determined by absorbance at 280 nm (extinction coefficient = 29,910 M^−1^ cm^−1^) measured by NanoDrop 2000c spectrophotometer (Thermo scientific).

#### Nanodisc reconstitution of *Hc*KCR1 WT, *Hc*KCR2 WT, and *Hc*KCR1 H225F mutant

Prior to nanodisc reconstitution, 30 mg SoyPC (Sigma P3644-25G) was dissolved in 500 μL chloroform and dried using a nitrogen stream to form a lipid film. The residual chloroform was further removed by overnight vacuum desiccation. Lipid films were rehydrated in buffer containing 1% DDM, 20 mMHEPES-NaOH pH 7.5, 100mM NaCl, resulting in a clear 10 mM lipid stock solution.

*Hc*KCR1 WT was reconstituted into nanodiscs formed by the scaffold protein MSP1E3D1 and SoyPC at a molar ratio of 1:4:400 (monomer ratio: *Hc*KCR WT, MSP1E3D1, SoyPC). First, freshly purified *Hc*KCR1 WT in DDM SEC buffer (0.03% DDM, 20 mMHEPES-NaOH pH 7.5,100 mM NaCl) was mixed with SoyPC and incubated on ice for 20 min. Purified MSP1E3D1 was then added to mess up to total solution volume of 750 μL, and gently mixed on rotator at 4°C for 10 min. Final concentrations were 14.5 μM *Hc*KCR1 WT, 58.2 μM MSP1E3D1, and 5.8 mM SoyPC, respectively. Detergents were removed by stepwise addition of Bio-Beads SM2 (Bio-Rad). Prior to use, Bio-Beads were washed by sonication in methanol, water, and buffer containing 20 mMHEPES-NaOH pH7.5,100 mM NaCl with an ultrasonic bath sonicator and weighed after liquid was removed by a P1000 tip. As the first batch, 100 mg Bio-Beads (final concentration of 133 mg mL^−1^) was added, and the mixture was gently rotated at 4°C for 12 h. The second batch of Bio-Beads (equal amount) was added and further rotated at 4°C for 2.5 h. The Bio-Beads were removed by passage through a PolyPrep column (Bio-Spin column, Bio-Rad), and the lysate was ultracentrifuged (TLA 55 rotor, 71,680 ×gfor 30 min) before size-exclusion chromatography on a Superdex 200 Increase 10/300 GL column (Cytiva), equilibrated in buffer containing 20 mMHEPES-NaOH pH7.5, 100 mM NaCl. The peak fractions were collected and concentrated to approximately 6 mg mL^−1^ estimated based on the absorbance (A 280) value of 16, using an Amicon ultra 50 kDa molecular weight cutoff centrifugal filter unit (Merck Millipore).

*Hc*KCR2 WT was reconstituted into nanodiscs basically in the same manner as *Hc*KCR1 WT. In brief, *Hc*KCR2 WT, MSP1E3D1 and SoyPC were mixed at a molar ratio of 1:4:100, with the final concentration of 41 μM, 164 μM, and 4.1 mM, respectively. The total solution volume was 750 μL. Detergents were removed by stepwise addition of Bio-Beads SM2 (Bio-Rad). The first Bio-Beads batch amount was 25 mg. After rotation at 4°C for 12 h, 40 mg of fresh Bio-Beads were added every 12 h, twice in total. *Hc*KCR2 WT in a nanodisc was purified through size-exclusion chromatography and concentrated to approximately 12 mg mL^−1^ estimated based on the absorbance (A 280) value of 30, using an Amicon ultra 50 kDa molecular weight cutoff centrifugal filter unit (Merck Millipore).

For the *Hc*KCR1 H225F mutant, sodium was replaced with potassium for all steps of the purification. *Hc*KCR1 H225F, MSP1E3D1 and SoyPC were mixed at a molar ratio of 1:4:50, with the final concentration of 29 μM, 117 μM, and 1.5 mM, respectively. The total solution volume was 750 μL. Detergents were removed by stepwise addition of Bio-Beads SM2 (Bio-Rad). The first Bio-Beads batch amount was 25 mg. After rotation at 4°C for 12 h, 40 mg of fresh Bio-Beads were added every 12 h, twice in total. *Hc*KCR1 H225F mutant in a nanodisc was purified through size-exclusion chromatography and concentrated to approximately 10 mg mL^−1^ estimated based on the absorbance (A 280) value of 24, using an Amicon ultra 50 kDa molecular weight cutoff centrifugal filter unit (Merck Millipore).

#### Cryo-EM grid preparation of nanodisc-reconstituted *Hc*KCR1 WT, *Hc*KCR2 WT, and *Hc*KCR1 H225F mutant

Prior to grid preparation, the sample was centrifuged at 20,380 ×g for 30 min at 4°C. The grids were glow-discharged with a PIB-10 plasma ion bombarder (Vacuum Device) at approximately 10 mA current with the dial setting of 2 min for both sides. 3 μL of protein solution was applied to freshly glow-discharged Quantifoil R1.2/1.3 Au 300 mesh holey carbon grid in dark room with dim red light. Samples were vitrified by plunging into liquid ethane cooled by liquid nitrogen with a FEI Vitrobot Mark IV (Thermo Fisher Scientific) at 4°C with 100% humidity. The blotting force was set as 10. The waiting and blotting time were 10 sand 4 s, respectively. All grid preparations were performed under dim red-light conditions.

#### Cryo-EM data acquisition and image processing of *Hc*KCR1 WT

Cryo-EM images were acquired under operation at an accelerating voltage of 300 kV on a Krios G3i microscope (Thermo Fisher Scientific) equipped with a Gatan BioQuantum energy filter and a K3 direct detection camera in the electron counting mode. The movie dataset was collected in standard mode, using a nine-hole image shift strategy in the SerialEM software,^84^ with a nominal defocus range of −0.8 to −1.6 μm. The 5,445 movies were acquired at a dose rate of 14.313 e^−^/pixel/s, at a pixel size of 0.83 Å and a total dose of 48 e^−^/Å^2^.

The data processing was performed using the cryoSPARC v3.2.0 software platform.^92^ The collected 5,445 movies were subjected to Patch motion correction and Patch CTF estimation in cryoSPARC. Initial particles were picked from all micrographs using Blob picker and were extracted using a box size of 280 pixels. 407,781 particles were selected after 2D classification from 2,439,182 particles. The following *Ab-initio* reconstruction, Heterogeneous refinement, and Non-uniform refinement^93^ enabled us to reconstruct the 2.92 Å map (C1 symmetry) with 130,130 particles. Further particles were picked by Template picker and Topaz picker^94^ and subjected to 2D classification followed by Heterogeneous refinement. Non-uniform refinement after removing of the duplicated particles enable us to obtain 2.60 Å map (C3 symmetry) with 917,464 particles. The subsequent 2D classification, global CTF refinement,^95^ and non-uniform refinement yielded the final map at a global resolution of 2.58 Å. Note that following our initial report,^83^ the second and third *Hc*KCR1 structures were determined in detergent (3.17−3.35 Å)^96^ and peptidiscs (2.88 Å),^97^ respectively, suggesting that *Hc*KCR1 can tolerate a wide range of membrane/lipid environments for structural studies.

#### Cryo-EM data acquisition and image processing of *Hc*KCR2 WT

Cryo-EM images were acquired under operation at an accelerating voltage of 300 kV on a Krios G4 microscope (Thermo Fisher Scientific) equipped with a Gatan BioQuantum energy filter and a K3 direct detection camera in the electron counting mode. The movie dataset was collected in standard mode, using the fringe-free imaging (FFI) and aberration-free image shift (AFIS) strategy in the EPU software (Thermo Fisher Scientific), with a nominal defocus range of −0.6 to −1.6 μm. The 7,718 movies were acquired at a dose rate of 17.5 e^−^/pixel/s, at a pixel size of 0.83 A and a total dose of 51 e^−^/Å^2^.

The data processing was performed using the cryoSPARC v3.3.2 software platform. The collected 7,718 movies were subjected to Patch motion correction and Patch CTF estimation in cryoSPARC. Particles were picked from all micrographs by Blob picker, Template picker, and Topaz picker, resulted in 3,382,955 particles, 5,852,598 particles, and 2,844,575 particles, respectively. These particle subsets were subjected to 2D classification and subsequent Heterogeneous refinement. The particles in the best classes were 508,364 particles for the Blob picker, 777,572 particles for the Template picker, and 519,445 particles for the Topaz picker, respectively. After removal of duplicates, 1,243,623 particles were selected and subjected to Non-uniform refinement, resulting in a 2.66 Å map (C3 symmetry). The additional Heterogeneous refinement, Non-uniform refinement, Local motion correction,^98^ and another Non-uniform refinement along with defocus refinement and global CTF refinement yielded the final map at a global resolution of 2.53 Å.

#### Cryo-EM data acquisition and image processing of *Hc*KCR1 H225F mutant

Cryo-EM images were acquired at 300 kV on a Krios G3i microscope (Thermo Fisher Scientific) equipped with a Gatan BioQuantum energy filter and a K3 direct detection camera in the electron counting mode. The movie dataset was collected in standard mode, using the fringe-free imaging (FFI) and aberration-free image shift (AFIS) strategy in the EPU software (Thermo Fisher Scientific), with a nominal defocus range of −0.8 to −1.6 μm. The 6,654 movies were acquired at a dose rate of 14.2 e^−^/pixel/s, at a pixel size of 0.83 Å and a total dose of 48 e^−^/Å^2^.

The data processing was performed using the cryoSPARC v4.0.0 software platform. The collected 6,654 movies were subjected to Patch motion correction and Patch CTF estimation in cryoSPARC. Particles were picked from all micrographs by Template picker, and Topaz picker, resulting in 4,599,946 particles, and 762,851 particles, respectively. These particle subsets were subjected to 2D classification and subsequent Heterogeneous refinement. The best classes contained 745,810 particles for the Template picker, and 236,191 for the Topaz picker, respectively. After removal of duplicates, 896,040 particles were selected and subjected to Non-uniform refinement with iterative CTF refinement, resulting in a 2.83 Å map (C3 symmetry).

The particles were transferred to RELION 4.0 and were subjected to masked 3D classification without alignment. Selected 180,294 particles were transferred back to cryoSPARC and the additional Non-uniform refinement yielded the final map at a global resolution of 2.66 Å.

#### Model building and refinement

Initial models of *Hc*KCR1 WT and *Hc*KCR2 WT were formed by rigid body fitting of the predicted models of *Hc*KCR1 WT and *Hc*KCR2 WT, respectively, generated using locally installed AlphaFold2.^99^ This starting model was then subjected to iterative rounds of manual and automated refinement in Coot^89^ and Refmac5^100^ in Servalcat pipeline,^90^ respectively. The Refmac5 refinement was performed with the constraint of C3 symmetry. The initial model for *Hc*KCR1 H225F mutant was the refined model of *Hc*KCR1 WT.

The final model was visually inspected for general fit to the map, and geometry was further evaluated using Molprobity.^101^ The final refinement statistics are summarized in Table S1. All molecular graphics figures were prepared with UCSF Chimera,^87^ UCSF Chi-meraX,^88^ CueMol2 (http://www.cuemol.org), and PyMOL.^102^

#### Pore analysis

The ion-conducting pore pathways were calculated by the software HOLLOW 1.3 with a grid-spacing of 1.0 Å^103^

#### Measurement of UV absorption spectra and pH titration

To investigate the pH dependence of the absorption spectra of *Hc*KCR1 and *Hc*KCR2, 10 mg mL^−1^ purified protein solution was 100-fold diluted in buffer containing 0.05% DDM, 100 mM NaCl, and 100 mM of either citric acid pH 2.2, citric acid pH 3.0, sodium acetate pH 4.0, sodium citrate pH 5.0, sodium cacodylate pH 6.0, HEPES-NaOH pH 7.0, Tris-HCl pH 8.0, *N*-cyclohexyl-2-aminoe-thanesulfonicacid (CHES) pH 9.0, 3-(cyclohexylamino)-1-propanesulfonic acid (CAPS) pH 10.0, or CAPS pH 11.0. The StockOptions pH Buffer Kit (Hampton research) was used for buffer preparation except for CHES pH 9.0 (Nacalai). The absorption spectra were measured with a V-750 UV-visible spectrometer (JASCO) at room temperature.

#### Laser flash photolysis

For the laser flash photolysis spectroscopy, *Hc*KCR1 WT and D105N were reconstituted in azolectin (11145, Sigma-Aldrich, Merck, Germany) with a protein-to-lipid molar ratio of 1:50 in 100 mM KCl, 20 mMHEPES-KOH pH 7.5. OD of the proteo-liposome suspensions was adjusted to ~0.8 (protein concentration ~0.2-0.3 mg/mL) at the absorption maximum wavelengths. The laser flash photolysis measurement was conducted as previously described.^59^ Nano-second pulses from an optical parametric oscillator (5.7 mJ/ pulse cm^2^, basiScan, Spectra-Physics, CA) pumped by the third harmonics of Nd–YAG laser (λ = 355 nm, INDI40, Spectra-Physics, CA) were used for the excitation of *Hc*KCR1 WT and D105N at λ_exc_ = 510 and 500 nm, respectively. The transient absorption spectra were obtained by monitoring the intensity change of white-light from a Xe-arc lamp (L9289-01, Hamamatsu Photonics, Japan) passed through the sample with an ICCD linear array detector (C8808-01, Hamamatsu, Japan). To increase the signal-to-noise (S/N) ratio, 45–60 spectra were averaged, and the singular-value-decomposition (SVD) analysis was applied. To measure the time-evolution of transient absorption change at specific wavelengths, the output of a Xe-arc lamp (L9289-01, Hamamatsu Photonics, Japan) was monochromated by monochromators (S-10, SOMA OPTICS, Japan) and the change in the intensity after the photo-excitation was monitored with a photomultiplier tube (R10699, Hamamatsu Photonics, Japan). To increase the S/N ratio, 100–200 signals were averaged. Time-series traces of absorption change for *Hc*KCR1 WT and D105N mutant at 617 nm (light red, K), 480 nm (light green, L/N), 384 nm (light purple, M_1_), and 404 nm (light blue, M_2_) are shown in Figure 2D. The corresponding wavelengths for *Hc*KCR1 D105N are 609 nm (red, K), 515 nm (green, L/N), 378 nm (purple, M′), and 394 nm (blue, M”).

To measure the transient absorption change of pyranine due to proton release and uptake by *Hc*KCR1 WT, the protein was solubilized in 100 mM KCl, 0.05% DDM, and pH was adjusted to 7.2 close to the pKa of pyranine by adding NaOH, and then 40 μM pyranine (L11252, Wako, Japan) was added. The formation and disappearance of the protonated form of pyranine were monitored at 454 nm by subtracting the transient absorption change obtained without pyranine from that obtained with pyranine as previously reported.^104^

#### High-performance liquid chromatography (HPLC) analysis of retinal isomers in *Hc*KCR1 WT

The HPLC analysis of retinal isomers was conducted as described elsewhere^38^ with a slight modification. The purified sample was incubated at 4°C overnight in the dark prior to the HPLC analysis. A 30 μL sample and 120 μL of 90% (v/v) methanol aqueous solution and 10 μL of 2 M hydroxylamine (NH_2_OH) were added to the sample. Then, retinal oxime hydrolyzed from the retinal chromophore in *Hc*KCR1 WT was extracted with 500 μL of *n*-hexane. A 200 μL of the extract was injected into an HPLC system equipped with a silica column (particle size 3 μm, 150 × 6.0 mm; Pack SIL, YMC, Japan), a pump (PU-4580, JASCO, Japan), and a UV–visible detector (UV-4570, JASCO, Japan). As the mobile-phase solvent, *n*-hexane containing 15% ethyl acetate and 0.15% ethanol was used at a flow rate of 1.0 mL min^−1^. Illumination was performed with green light (510 ± 5 nm) for 60 s. The molar composition of the retinal isomers the sample was calculated with the molar extinction coefficient at 360 nm for each isomer (all-*trans*-15-*syn*: 54,900 M^−1^ cm^−1^ ; all- *trans*-15-*anti*: 51,600 M^−1^ cm^−1^; 13-*cis*-15-*syn*, 49,000 M^−1^ cm^−1^; 13-*cis*-15-*anti*: 52,100 M^−1^ cm^−1^; 11-*cis*-15-*syn*: 35,000 M^−1^ cm^−1^; 11-*cis*-15-*anti*: 29,600 M^−1^ cm^−1^; 9-*cis*-15-*syn*: 39,300 M^−1^ cm^−1^; 9-*cis*-15-*anti*: 30,600 M^−1^ cm^−1^).^105,106^

#### Laser patch clamp

The electrophysiological assays of *Hc*KCR1 were carried out using ND7/23 cells, as described previously^107^ with a slight modification. Briefly, ND7/23 cells were grown in Dulbecco’s modified Eagle’s medium (D-MEM, FUJIFILM Wako Pure Chemical Co., Osaka, Japan) supplemented with 5% fetal bovine serum (FBS) under a 5% CO_2_ atmosphere at 37°C. Eight hours after the transfection, the medium was replaced by D-MEM containing 5% FBS, 50 ng/mL nerve growth factor-7S (Sigma-Aldrich, St. Louis, MO), 1 mM N6,2′- *O*-dibutyryladenosine-3′,5′-cyclic monophosphate sodium salt (Nacalai tesque, Kyoto, Japan), and 1 μM Cytosine-1-β-D(+)-arabi-nofuranoside (FUJIFILM Wako Pure Chemical Co., Osaka, Japan). The coding sequence of *Hc*KCR1 was fused to a Kir2.1 membrane trafficking signal, eYFP, and an ER-export signal.^108^ The gene was cloned into a vector behind a CMV-promotor and the expression plasmids were transiently transfected in ND7/23 cells using LipofectamineTM 3000 transfection reagent (Thermo Fisher Scientific Inc., Waltham, MA) and electrophysiological recordings were conducted at 2–3 days after the transfection. The transfected cells were identified by the presence of eYFP fluorescence under an up-right microscope (BX50WI, Olympus, Tokyo, Japan).

All experiments were carried out at room temperature (20°C–22°C). Currents were recorded using an EPC-8 amplifier (HEKA Electronic, Lambrecht, Germany) under a whole-cell patch clamp configuration. The internal pipette solution contained 121.2 mM KOH, 90.9 mM glutamate, 5 mM Na_2_EGTA, 49.2 mMHEPES, 2.53 mM MgCl_2_, 2.5 mM MgATP, 0.0025 mM ATR (pH 7.4 adjusted with HCl). Extracellular solution contained 138 mM NaCl, 3 mM KCl, 2.5 mM CaCl_2_, 1 mM MgCl_2_, 4 mM NaOH, and 10 mMHEPES at pH 7.4 (with 11 mM glucose added up to 310 mOsm). The pipette resistance was adjusted to 3–6 MΩ (3.7 ± 0.4, n = 7) with a series resistance of 6–11 MΩ (8.3 ± 0.8) and a cell capacitance of 32–216 pF (83 ± 21) with the extracellular/intracellular solutions. In every experiment, the series resistance was compensated.

While voltage-clamping at a holding potential, a laser flash (3–5 ns) at 532 nm (Nd:YAG laser, Minilite II, Continuum, San Jose, CA) was illuminated through an objective lens (LUMPlan FL 40×, NA 0.80W, Olympus, Japan). The timing of laser flash was set to be time 0 according to the photodiode response under the sample. The measurements were conducted with a holding potential of 0 mV at every 15 s. The data were filtered at 1 kHz, sampled at 250 kHz (Digidata1440 A/D, Molecular Devices Co., Sunnyvale, CA), collected using pClamp10.3 software (Molecular Devices Co., Sunnyvale, CA), and stored in a computer. Five current responses were averaged and served for the following analyses. Using the simplex method of nonlinear least-squares (IgorPro 9, WaveMetrics, Portland, OR), the kinetics of photo-current were fitted by a triple-exponential function.

#### ATR-FTIR spectroscopy

Ion binding to *Hc*KCR1 WT was monitored by ATR-FTIR spectroscopy as described previously,^59,109–112^ except for some minor modifications for reconstitution into the membrane. In ATR-FTIR spectroscopy, rhodopsins are normally reconstituted into lipids for forming a film on the ATR-prism. Thus, sample was reconstituted with a protein-to-lipid (asolectin; Sigma-Aldrich) molar ratio of 1:20, by removing the *n*-dodecyl-β-D-maltoside (DDM) with Bio-Beads SM-2 (Bio-Rad) at 4°C in dark condition. The *Hc*KCR1 WT sample in asolectin liposomes was washed repeatedly with a buffer containing 2 mM K_2_HPO_4_/KH_2_PO_4_ (pH 7.5) and collected by ultracentrifuging for 20 min at 222,000 x g at 4°C in dark condition. The lipid-reconstituted *Hc*KCR1 WT was placed on the surface of a silicon ATR crystal (Smiths, three internal total reflections) and naturally dried. The sample was then rehydrated with the buffer at a flow rate of 0.6 mL min^−1^, and temperature was maintained at 20°C by circulating water. The perfusion buffer is composed of 200 mM NaCl, 200 mM Tris-HCl, pH 7.5 (buffer A) and 200 mM KCl, 200 mM Tris-HCl, pH 7.5 (buffer B). In the case of anion binding experiments, the perfusion buffer was replaced with 200 mM NaCl, 20 mMHEPES-NaOH, pH 7.5 (buffer A) and 200 mM NaBr, 20 mM HEPES-NaOH, pH 7.5 (buffer B), respectively.

ATR-FTIR spectra were recorded in kinetics mode at 2 cm^−1^ resolution, renge of 4000-700 cm^−1^ using an FTIR spectrometer (Agilent) equipped with a liquid nitrogen-cooled mercury-cadmium-telluride (MCT) detector (an average of 1710 interferograms per 15 min). Ion binding-induced difference spectra were measured by exchanging the buffer A and buffer B. The cycling procedure is shown in Figure S3G, and the difference spectra were calculated as the averaged spectra in buffer B minus buffer A. The absorption of unbound salt and the water vapor absorption were subtracted from the raw spectra for spectral correction. The drift of baseline caused by protein swelling/shrinkage was also subtracted in order to obtain the final spectra.^110^

Light-induced structural changes of *Hc*KCR1 WT were also measured by ATR-FTIR as shown in Figure S3G. Since ATR-FTIR experimental setup has been optimized for ion perfusion-induced difference spectroscopy using a solution exchange system, we have modified experimental setup that enables light irradiation experiment. A light source was installed above the ATR prism. In addition, an optical filter and a condenser lens were placed directly under the light source. To obtain the ion binding-induced difference spectra under the light illumination condition, light minus dark difference spectra under perfusing the different solution between buffer A and buffer B was subtracted each other. The absorption of unbound salt and the water vapor absorption were subtracted from the raw spectra for spectral correction. The drift of baseline caused by protein swelling/shrinkage was also subtracted in order to obtain the final spectra.^110^

#### Protein expression, and purification of KR2

KR2 is expressed and purified as described earlier^113^ with the following modifications. Briefly, KR2 gene in pET-43a(+) was transformed in *E. coli* C41 (DE3) cells. Cells were grown at 37°C with shaking to an OD_600_ of 0.5–1.0, and then expression of KR2 was induced by addition of 0.5 mM IPTG and supplemented with 10 μM ATR (Sigma). Cells were further grown for at 37°C for 2.5 h, and cells were harvested by centrifugation. Cell pellets were lysed by sonication with a hypotonic lysis buffer (20 mM HEPES-NaOH pH 7.5, 20 mM NaCl, 10 mM MgCl_2_, 0.1 mM PMSF), and membrane fraction was collected by ultracentrifugation (45Ti rotor, 125,000 ×g for 1 h). The membrane fraction was homogenized with a glass douncer in a membrane storage buffer (20 mM HEPES-NaOH pH 7.5, 500 mM NaCl, 10 mM imidazole, 20% glycerol, 0.1 mM PMSF), flash frozen in liquid nitrogen, and stored at −80°C until use.

The membrane fraction was solubilized in a solubilization buffer (1.5% *n*-dodecyl-β-D-maltoside (DDM) (Glycon), 20 mM HEPES-NaOH pH 7.5, 500 mM NaCl, 20% glycerol, 5 mM imidazole, 0.1 mM PMSF) and solubilized at 4°C for 2 h. The insoluble cell debris was removed by ultracentrifugation (45Ti rotor, 125,000 ×g, 45 min), and the supernatant was mixed with the Ni-NTA superflow resin (QIAGEN) at 4°C for 1 h. The Ni-NTA resin was loaded onto an open chromatography column, washed with 2.5 column volumes of wash buffer (0.05% DDM, 20 mM HEPES-NaOH pH 7.5, 300 mM NaCl, and 30 m Mimidazole) three times, and eluted by elution buffer (0.05% DDM, 20 mM HEPES-NaOH pH 7.5, 300 mM NaCl, and 300 mM imidazole).

The eluted KR2 was dialyzed in buffer containing 20 mM HEPES-NaOH pH 7.5, 300 mM NaCl, and concentrated to approximately 5 mg mL^−1^ using an Amicon ultra 100 kDa molecular weight cutoff centrifugal filter unit (Merck Millipore). The concentrated samples were ultracentrifuged (TLA 55 rotor, 71,680 ×g for 30 min) before size-exclusion chromatography on a Superdex 200 Increase 10/300 GL column (Cytiva), equilibrated in DDM SEC buffer (0.03% DDM, 20 mM HEPES-NaOH pH 7.5, 300 mM NaCl). The peak fractions of the protein were collected and concentrated to approximately 10 mg mL^−1^ and stored at −80°C after flash freezing in liquid nitrogen. The concentration was determined by absorbance at 280 nm measured by NanoDrop 2000c spectrophotometer (Thermo-scientific).

#### Analysis of protein thermal stability using FSEC-TS

The fluorescence-detection size-exclusion-chromatography-based thermostability (FSEC-TS) assay^60^ was performed to evaluate ion binding to proteins. In this assay, the types of ions in the sample solution were varied and heated to various temperatures, and the thermal stability was evaluated by the decay in peak intensity of the SEC profile.

Purified samples of *Hc*KCR1 WT and KR2 were treated with ions by dialysis (buffer: 0.03% DDM, 20 mM Tris-HCl (pH 8.0), and either 100 mMNaCl, KCl, or CsCl, respectively). Purified *Hc*KCR1 WT and KR2 protein at 0.1 mg mL^−1^ were separated into 60 μL aliquots in PCR tubes, heated at a range of temperatures from 20°C to 95°C for 15 min, and centrifuged at 20,380 g for 15 min. Then, 20 μL of the supernatant was loaded onto a TSKgel 3000 SW_XL_ column (TOSOH bioscience) equipped with Prominence (Shimadzu), pre-equilibrated with DDM SEC buffer (0.03% DDM, 100 mM NaCl, 20 mM HEPES pH 7.5), and run at a flow rate of 0.6 mL min^−1^. The experiment was performed in triplicate. The peak heights of 280 nm absorbance were normalized to that from samples incubated at 4°C, and the data were fit to a 4-parameter logistic curve using the GraphPad Prism software.

#### Microscale thermophoresis (MST)

Microscale thermophoresis experiments were performed on a NanoTemper Monolith (NanoTemper Technologies). C-terminal His_6_-tagged KR2 was purified through size exclusion chromatography in a final buffer containing 0.03% DDM, 100 mM NaCl, and 20 mM HEPES-NaOH pH 7.5. The final buffer of *Hc*KCR1 WT with C-terminal His_10_ tag contained 0.03% DDM, 100 mM KCl, and 20 mM HEPES-KOH pH 7.5. The sample was labeled according to manufacturer instructions with RED-tris-NTA second-generation dye (NanoTemper Technologies). The concentration of protein was constant at 200 nM; For KR2 and *Hc*KCR1 WT, either NaCl or KCl was added, respectively, in a 2-fold serial dilution at a final concentration ranging from 7.81 mM to 1 M, with 20 mM HEPES (pH 7.5) and 0.03% DDM. After 1 h incubation at room temperature, the samples were centrifuged (20,380 × g, 10 min) to remove the denatured proteins and incubated for 30 min at room temperature. Thermophoresis was measured at 25°C with an excitation power of 20% and an MST power of 40%. Measurement protocol times were as follows: fluorescence before 3 s, 20 s MST, and 1 s after MST. The thermophoresis measurements were performed three times. Reported *K_D_* values were calculated with GraphPad Prism software.

#### *In vitro* electrophysiology in HEK293 cells

Cells and devices for the measurement were prepared as described previously.^38^ Briefly, HEK293 cells (Thermo Fisher Scientific) expressing opsins were placed in an extracellular Tyrode medium (150 mM NaCl, 4 mM KCl, 2 mM CaCl_2_, 2 mM MgCl_2_, 10 mM HEPES pH 7.4, and 10 mM glucose). Borosilicate pipettes (Harvard Apparatus, with resistance of 4–6 mOhm) were filled with intra-cellular medium (140 mM potassium-gluconate, 10 mM EGTA, 2 mM MgCl_2_ and 10 mM HEPES pH 7.2). After break-in, cells were held for at least 5 min before recording to ensure cell health and stability of the recording. Light was delivered with the Lumencor Spectra X Light engine with 470 nm and 560 nm filters for blue and orange light delivery, respectively. Light stimulation was for 1 s, with 1.0 mW/mm^2^ light power density, and all recordings were performed in triplicate to ensure stable and reproducible data. Recordings were randomized in order across conditions to counterbalance for unknown variables.

To estimate reversal potentials (E_rev_), cells were voltage clamped at −86 mV, and stepped to test voltages from −96 mV to +4 mV with 10 mV spacing (after liquid junction potential correction). Light stimuli were introduced after 1 s of holding voltage change, followed by a 1-s dark period while maintaining the stepped voltage value. Between each sweep, a 30-s dark period was imposed to prevent opsin desensitization. E_rev_ for each cell was extrapolated from an I-V curve generated from each recording. For channel kinetics and photocurrent amplitude estimation, to generate large driving forces and thus currents suitable for biophysical quantification, cells were stepped to potentials 70 mV more depolarized than E_rev_, except for variants with E_rev_ close to 0 mV (the ’excitatory’ variants Y222A and W102Q) which were stepped 70 mV more hyperpolarized than E_rev_. Inhibitory variants were held at 0 mV (except Y222F at +20 mV and excitatory variants Y222A and W102Q at −70 mV to optimize current property detection and analysis).

Data were analyzed in Clampfit software (Axon Instruments) after smoothening using a lowpass Gaussian filter with a −3 dB cutoff for signal attenuation and noise reduction at 1,000 Hz. Liquid junction potentials were corrected using the Clampex built-in liquid junction potential calculator as previously described.^38^ The relative ion permeability of sodium and potassium (*P*_K_/*P*_Na_) was calculated using the Goldman-Hodgkin-Katz equation, with T = 298K, *P_Cl_* = 0, F = 96485 C/mol and R = 8.314 J/K·mol.

$$V_{m}=\frac{RT}{F}\ln(\frac{p_{K}{\left[K^{+}\right]}_{o}+p_{Na}{\left[{\text{Na}}^{+}\right]}_{o}+p_{Cl}{\left[{\text{Cl}}^{-}\right]}_{0}}{p_{K}{\left[K^{+}\right]}_{i}+p_{\text{Na}}{\left[{\text{Na}}^{+}\right]}_{i}+p_{\text{Cl}}{\left[{\text{Cl}}^{-}\right]}_{i}})$$

Statistical analysis was performed with one-way ANOVA and the Kruskal–Wallis test for non-parametric data, using Prism 7 (GraphPad) software. Data collection across opsins was randomized and distributed to minimize across-group differences in expression time, room temperature, and related experimental factors.

#### Ion selectivity testing in HEK293 cells

HEK293 cells and devices for the measurement were prepared as described in the previous section. For the high sodium extracel-lular/high potassium intracellular condition, we used sodium bath solution containing 150 mM NaCl, 2 mM CaCl_2_, 2 mM MgCl_2_, 10 mMHEPES pH 7.3 with 10 mM glucose, along with potassium pipette solution containing 150 mM KCl, 2 mM CaCl_2_, 2 mM MgCl_2_, 10mMHEPES pH 7.3, and 10 mM glucose. For the high potassium extracellular/high sodium intracellular condition, NaCl and KCl concentrations were reversed, and all other ionic concentrations were kept constant. Reversal potentials were then recorded using with the same experimental procedure of *in vitro* HEK293 patching using standard extracellular and intracellular solutions; holding voltage started from −120 mV for Na^+^ extra/K^+^ intra experiments and −30 mV for K^+^ extra/Na^+^ intra experiments, to span relevant voltage ranges given the ion balance present. Liquid junction potentials were corrected using the Clampex built-in liquid junction potential calculator, which was calculated as 4.2 mV in this experiment.

For sodium extracellular/potassium intracellular condition, equilibrium potentials were measured by holding membrane potentials from −120 mV to +20 mV in steps of 10 mV (before LJP correction). for potassium extracellular/sodium intracellular, equilibrium potentials were measured by holding from −30 mV to 100 mV (before LJP correction) in 10 mV steps. The relative ion permeability of sodium and potassium (*P*_K_/*P*_Na_) was calculated using the Goldman-Hodgkin-Katz equation as stated above.

To test intracellular guanidinium blockage, an intracellular buffer containing 150 mM GuHCl, 2 mM CaCl_2_, 2 mM MgCl_2_, 10 mMHEPES pH 7.3, and 10 mM glucose was used with a typical high sodium extracellular buffer. To test extracellular Cs^+^ or Li^+^, extracellular buffers with 150 mM CsCl or LiCl with 10 mMHEPES pH 7.3,2 mM CaCl_2_,2 mM MgCl_2_ with 10 mM glucose were used with a regular high K^+^ intracellular buffer. Experiments were conducted using the same procedure as Na^+^ extra/K^+^ intra experiments.

Statistical analysis was performed with one-way ANOVA and the Kruskal–Wallis test for non-parametric data, using Prism 7 (GraphPad) software. Data collection across opsins was randomized and distributed to minimize across-group differences in expression time, room temperature, and related experimental factors.

#### System setup for molecular dynamics simulations

We performed two sets of MD simulations: (1) to study the selectivity filter and (2) to study the interaction of potassium ions with D116. To study the selectivity filter, we simulated the *Hc*KCR1 WT as well as the H225F, and H225A mutants. The WT and H225F simulations were initiated using the structures reported in this manuscript and the H225A structure was modeled by removing the appropriate atoms from the WT structure. To study the interaction of potassium ions with D116, we simulated *Hc*KCR1 WT and the D116N mutant. The WT simulations were initiated from an earlier refinement of the structure reported here which has no substantial differences in the intracellular side of the protein and the D116N mutant was modeled such that the positions of all the common atoms were retained. In this set of simulations, to study the exit pathway of ions from the channel, we placed a K^+^ in both the intracellular and extracellular vestibules near D116 and D205, respectively. For the first set of simulations, we performed six independent replicates for each simulation condition, and for the second set of simulations, we performed three independent replicates for each simulation condition, each 500 ns in length. For each simulation, initial atom velocities were assigned randomly and independently.

The structures were aligned to the Orientations of Proteins in Membranes^114^ entry for PDB accession code: 1M0L^115^ (bacteriorhodopsin). Prime (Schrödinger)^116^ was used to model missing side chains, and to add capping groups to protein chain termini. The Crosslink Proteins tool (Schrödinger) was used to model unresolved portions of ECL2, ICL3, and ECL3. Parameters for the ligands were generated using the Paramchem webserver.^117,118^ Only in the second set of simulations, Dowser software was used to add waters to cavities within the protein structure.^119^ Six POPC lipids were modeled in the center of the trimer; three in the extracellular leaflet and three in the intracellular leaflet. Protonation states of all titratable residues were assigned at pH 7. Histidine residues were modeled as neutral, with a hydrogen atom bound to either the delta or epsilon nitrogen depending on which tautomeric state optimized the local hydrogen-bonding network. Using Dabble,^120^ the prepared protein structures were inserted into a pre-equilibrated palmitoyl-oleoyl-phosphatidylcholine (POPC) bilayer, the system was solvated, and sodium, potassium, and chloride ions were added to neutralize the system. In the first set of simulations, sodium, potassium, and chloride were added to obtain final concentrations of 75 mM, 75 mM, and 150 mM, respectively and, in the second set of simulations, potassium and chloride were added to obtain a final concentration of 150 mM each. The final systems comprised approximately 101,000 atoms, and system dimensions were approximately 105 × 105 × 95 Å.

#### Molecular dynamics simulation and analysis protocols

We used the CHARMM36m force field for proteins, the CHARMM36 force field for lipids and ions, and the TIP3P model for waters.^121–123^ Retinal parameters were obtained through personal communication with Scott Feller.^124^ The first set of simulations were performed using the Compute Unified Device Architecture (CUDA) version of particle-mesh Ewald molecular dynamics (PMEMD) in AMBER20^125^ on graphics processing units (GPUs) and the second set of simulations were performed using AMBER18.

Systems were first minimized using three rounds of minimization, each consisting of 500 cycles of steepest descent followed by 500 cycles of conjugate gradient optimization. 10.0 and 5.0 kcal mol^−1^ Å^−2^ harmonic restraints were applied to protein, lipids, and ligand for the first and second rounds of minimization, respectively. 1 kcal mol^−1^ Å^−2^ harmonic restraints were applied to protein and ligand for the third round of minimization. Systems were then heated from 0 K to 100 K in the NVT ensemble over 12.5 ps and then from 100 K to 298 K in the NPT ensemble over 125 ps, using 10.0 kcal mol^−1^ Å^−2^ harmonic restraints applied to protein and ligand heavy atoms. Subsequently, systems were equilibrated at 298 K and 1 bar in the NPT ensemble, with harmonic restraints on the protein and ligand non-hydrogen atoms tapered off by 1.0 kcal mol^−1^ ⋅Å^−2^ starting at 5.0 kcal mol^−1^ ⋅Å^−2^ in a stepwise fashion every 2 ns for 10 ns, and then by 0.1 kcal mol^−1^ ⋅Å^−2^ every 2 ns for 20 ns. Production simulations were performed without restraints at 310 K and 1 bar in the NPT ensemble using the Langevin thermostat and the Monte Carlo barostat, and using a timestep of 4.0 fs with hydrogen mass repartitioning.^126^ Bond lengths were constrained using the SHAKE algorithm.^127^ Non-bonded interactions were cut off at 9.0 Å, and long-range electrostatic interactions were calculated using the particle-mesh Ewald (PME) method with an Ewald coefficient of approximately 0.31 Å, and 4th order B-splines. The PME grid size was chosen such that the width of a grid cell was approximately 1 Å. Trajectory frames were saved every 200 ps during the production simulations.

To obtain a putative ion conduction pathway, we performed simulations with an artificial force applied to potassium ions pulling them from the solvent into the extracellular vestibule. In these simulations, the same equilibration protocol was performed, followed by 8 ns of unbiased simulation. A potassium ion was then pulled in line with the protein by applying a potential in the plane of the membrane with a flat bottom to 2.5 Å from the centroid of the protein, a harmonic potential to 4.5 Å, and thereafter a linear potential and a harmonic potential perpendicular to the membrane centered at 24 Å (roughly 6 Å from the selectivity filter) becoming linear with more than 2 Å of deviation with a force constant of 5 kcal/mol/Å^2^. The potassium ion was then pulled into the EV by applying a time dependent harmonic potential perpendicular to the plane of the membrane with a force constant of 100 kcal/mol/Å^2^ and a velocity of 5 Å/ns. All artificial forces were applied between the potassium ion and the centroid of the alpha-carbons of protein residues in transmembrane helices of one monomer of *Hc*KCR1. Throughout this simulation procedure, 0.1 kcal mol^−1^ ⋅Å^−2^ positional restraints were imposed on protein alpha-carbons to preclude deformation of the protein.

#### One-photon neuronal electrophysiology

Primary rat hippocampal cultured neurons were transfected with pAAV KCR-bearing plasmids and subsequently assessed in the same experimental setup as described in the HEK293 electrophysiology section. Voltage-clamp recordings were conducted in the presence of bath-applied tetrodotoxin (TTX, 1 mM, Tocris). To screen for action spectra, the cells were maintained at a resting potential of −20 mV and exposed to light at wavelengths (in nm) of 390, 438, 485, 513, 585, and 650, with 1.0 mW/mm^2^ light intensity for 1 s. Filters with corresponding peak wavelengths and 15−30 nm bandwidth were employed to generate the wavelengths. Channel kinetics and photocurrent amplitudes were evaluated at a holding membrane potential of −20 mV. To determine the reversal potential, the holding potential was increased from −106 to 4 mV in increments of 10 mV. As previously reported, the Clampex built-in liquid junction potential calculator was used to correct liquid junction potentials.

In order to conduct current clamp measurements for both *in vitro* and slice physiology, glutamatergic synaptic blockers CNQX and AP-V were used. Stereotactic surgeries were performed as previously described.^38^ For the slice physiology experiments, acute coronal slices with a thickness of 300 μm were prepared after intracardial perfusion with ice-cold N-methyl-D-glucamine (NMDG) containing cutting solution, which included 93 mM NMDG, 2.5 mM KCl, 25 mM glucose, 1.2 mM NaH_2_PO_4_, 10 mM MgSO_4_, 0.5 mM CaCl_2_, 30 mM NaHCO_3_, 5 mM Na ascorbate, 3 mM Na pyruvate, 2 mM thiourea, and 20 mMHEPES at a pH range of 7.3–7.4. Following this, the slices were incubated for 12 min at 34°C and then transferred to a room temperature oxygenated artificial cerebrospinal fluid (ACSF) solution containing 124 mM NaCl, 2.5 mM KCl, 24 mM NaHCO_3_,2 mM CaCl_2_, 2 mM MgSO_4_,1.2 mM NaH_2_PO_4_, 12.5 mM glucose, and 5 mMHEPES at a pH range of 7.3−7.4.

For photoinduced spike suppression, neurons were stimulated by injection of either 300 pA for 5 s (tonic, Figure 7E) or 3 ms of 700 pA current at 10 Hz (phasic, Figure 7F), and 560 nm light at 1.0 mW mm^−2^ was applied during the recording for 2 s during both tonic and phasic conditions. Data collection across opsins was randomized and distributed to minimize across-group differences in expression time, room temperature, and related experimental factors.

#### *In vitro* characterization preparatory to all-optical setup

Dissociated hippocampal neurons were cultured and transduced with both KCR2 (H225F) and XCaMP-R as previously described.^34,38^ One microliter viral suspension of KCR2 (H225F) (AAV8-CaMKIIα-KCR2(H225F)-EYFP, 3.0e12 vg/mL) mixed with 1 μL XCaMP-R^128^ (AAV8-CaMKIIα-XCaMP-R, 5.6e12 vg/mL) was added after 5 DIV. Cultured neurons were used between 12 and 14 DIV for experiments. Coverslips of cultured neurons were transferred from the culture medium to a recording bath filled with Tyrode solution containing (129 mM NaCl, 2 mM KCl, 30 mM glucose, 25 mM HEPES-NaOH pH 7.4, 1 mM MgCl_2_ and 3 mM CaCl_2_) supplemented with 10 μM CNQX and 25 μM APV to prevent contamination from spontaneous and recurrent synaptic activity. Optical stimulation and imaging were performed using a 40×/0.6-NA objective (Leica), sCMOS camera (Hamamatsu, ORCA-Flash4.0) and LED light source (Spectra X Light engine, Lumencor), all coupled to a Leica DMI 6000 B microscope. XCaMP-R were excited by 555 nm (Chroma, ET555/20×) with the Spectra X Light engine. XCaMP-R emission was reflected off a dual wavelength dichroic mirror (Semrock, FF573-Di01) for cyan light stimulation and passed through a single-band emission filter (Semrock, FF02-617/73-25). Opsins were activated with a Spectra X Light engine filtered either with 475 nm cyan light (Semrock, FF02-475/20–25,2.0 mW/mm^2^). Fluorescence of XCaMP-R was imaged using 555 nm (0.2 mW/mm^2^) laser light, respectively, without substantially activating opsin. Images were acquired at 10 Hz using MicroManager (http://micro-manager.org). Neurons were stimulated using an electrical field stimulation (50 mA, 1-ms current pulses applied across the coverslip), which was sufficient to reliably induce somatic spikes under our recording condition, with the use of a LabVIEW (National Instruments) and Master-8 pulse stimulator (A.M.P.I.). Electrical field stimulation was applied every 10 s. Light delivery was controlled by LabVIEW (National Instruments) and Master-8 pulse stimulator (A.M.P.I.) and applied 50 ms after the second and fourth electrical field stimulation. Imaging data were analyzed in MATLAB (MathWorks). Circular regions of interest (ROIs) were drawn manually based on the averaged image. We performed background subtraction before calculating Ca^2+^ signals. ΔF/F responses were calculated to normalize the signal in each ROI, by dividing by its mean fluorescence intensity of 3 s before the light delivery and subtracting 1.

#### Stereotactic surgeries

Wild-type male and female C57BL/67 mice were obtained from Jackson Laboratory. All stereotactic surgeries were performed with mice under isoflurane anesthesia (4% initially, maintained at 2–3%) with regular monitoring for stable respiratory rate and absent tail pinch response. The scalp was shaved, and mice were placed in the stereotactic apparatus and a heating pad was used to prevent hypothermia. A midline incision was made to expose the skull and small craniotomies were made above the injection sites using a Meisinger Carbide Burr size 1/4.

All virus dilutions were performed in ice-cold PBS and all viruses were produced at the Stanford Gene and Viral Vector Core. Virus injections were delivered with a 10 μL syringe (World Precision Instruments) and 33-gauge bevelled needle (World Precision Instruments), injected at 100 nL min^−1^ using an injection pump (World Precision Instruments). For fiber photometry experiments, mice were injected with either a mixture of AAV8-CaMKIIα-KCR2 (H225F)-EYFP (3.0e12 vg/ml) and AAV8-CaMKIIα-sRGECO^129^ (6.8e12 vg/ml), AAV8-CaMKIIα-sRGECO (3.4e12 vg/ml) alone or a mixture of AAV8-CaMKIIα-NpHR3.3-mCherry (7.6e12 vg/ml) and AAV8- CaMKIIα-GCaMP6m (8.8e12 vg/ml). 0.7 μL of virus was stereotactically injected unilaterally into the mPFC of 8–12 week old mice at 1.8 mm AP, 0.35 mm ML, and 2.4 mm DV from the bregma. Following injection, the injection needle was held at the injection site for 10 min then slowly withdrawn. Mice were administered 0.5–1.0 mg kg^−1^ subcutaneous buprenorphine-SR (ZooPharma) approximately 30 min before the end of the surgery for post-operative pain management.

#### FIP setup and analysis

We collected bulk fluorescence from targeted brain regions using a single optical fiber while delivering excitation light for fiber photometry as described previously.^38,128,130^ The illumination protocol repeats a sequence of three-frame sampling periods - one movement correction at 380 nm, one signal at 560 nm for sRGECO or 470 nm for GCaMP6m and one optogenetic modulation at 470 nm for KCR2 (H225F) or 594 nm for eNpHR3.3^129^ (Figure S7D). A low fluorescence 400-mm-diameter 0.66-NA mono fiberoptic cannula (Doric Lenses) was implanted above mPFC for fiber photometry. Cannulas were secured to the skull using a base layer of adhesive dental cement (C&B-Metabond, Parkell), followed by a second layer of cranioplastic cement (Ortho-Jet, Lang). Experiments were conducted 4–6 weeks later for FIP recordings to allow for sufficient viral expression and postsurgery recovery. One end of the patchcord terminated in an SMA connector (Thorlabs, SM1SMA) mounted at the working distance of the objective, and the other end terminated in 2.5-mm-diameter stainless steel ferrules. These ferrules were coupled via bronze sleeves (Doric, SLEEVE_BR_2.5) to ferrules implanted into a mouse. Fiber faces were imaged through a 20×/0.75-NA objective (Nikon, CFI Plan Apo Lambda 20×) through a series of reconfigurable dichroic mirrors.

In the standard KCR2 (H225F) and sRGECO pair configuration, three LEDs (Thorlabs, M385F1, M470F3, and M565F1) were used as excitation sources and each was paired with a bandpass filter (Semrock, FF01-380/14–25, LL01-475/28-25, and FF01- 560/14–25). In order to form a colinear excitation path using these three constitutive light sources, a sequence of dichroic filters is necessary for beam combination. First, the excitation path combines the optogenetic and signal light sources (M470F3) using a 525 nm long-pass dichroic mirror (Chroma, T525lpxr). This path is then combined with the control light source (M385F1) by use of a 425 nm long-pass dichroic (Chroma, T425LPXR). This triple excitation beam then reflects off a multiband dichroic mirror (Semrock, Di01-R405/488/561/635) toward the microscope objective, where it is coupled into the optical fiber patch cord and illuminates the sample. Fluorescence emission from the sample, after propagation from the fiber patch cord, will pass through this multi-bandpass dichroic and then through a pair of fluorescence emission filters (Semrock, FF01-446/523/600/677-25) before being collected by the tube lens (Thorlabs, AC254-035-A-ML) and imaged onto the sCMOS camera (Orca Flash v.4.0, Hamamatsu). Sample illumination powers of 2.5 μW and 10 μW (380 nm and 560 nm, respectively) were used at the far end of the patch cord. For NpHR3.3 and GCaMP6m pair configuration, the 565 nm LED was replaced with 594 nm laser (Opto Engine LLC, 50 mW). The 594 nm laser was filtered with a 590-10 nm bandpass filter (Thorlabs, FB590-10). Fluorescence emission from the sample will pass through triple multiband dichroic (Chroma, 69013bs) and then through a pair of fluorescence emission filters (Semrock, FF01-425/527/685-25) before being collected by the tube lens. Sample illumination powers of 2.5 μW and 10 μW (380 nm and 470 nm, respectively) were used at the far end of the patch cord.

A custom MATLAB (Mathworks, Natick, MA) GUI was written to control the sample illumination protocol as well as provide power modulation pulses to the LEDs (National Instruments, NI PCIe-6343-X) which temporally align the respective LED illumination with camera frame acquisition (HCImage, Hamamatsu). Maintaining a dedicated frame for optogenetic excitation faithfully removes any potential cross-excitation artifact from the isosbestic and signal sampling windows. The fluorescence signal was calculated with custom written MATLAB scripts. We fit a double exponential to a thresholded version of the fluorescence time series and subtracted the best fit from the unthresholded signal to account for slow bleaching artifacts. Fluorescence signal was smoothed with a 0.5 Hz low-pass filter and normalized within each mouse by calculating the ΔF/F as (F - baseline (F))/baseline (F), where the baseline was taken from the average during 5 s before optogenetic stimulation. Peak inhibited ΔF/F amplitude was calculated by subtracting the pre-stimulus baseline from the minimum value during stimulation period. Rebound ΔF/F amplitude was calculated from the maximum value during 10 s after the stimulus cessation.

#### Two-photon illumination-driven neuronal electrophysiology and Ca^2+^ imaging

Two-photon electrophysiology experiments were carried out using cultured hippocampal neurons with the same intracellular and extracellular solutions as those used for the one-photon electrophysiology characterization. The experiments were conducted using a Bruker Ultima microscope equipped with PrairieView v5.4 software and a Nikon 163/0.8 NA (CFI75) long-working distance objective for light delivery.

To perform the two-photon stimulation, we utilized spiral scanning through a defined spiral region of interest (ROI) with a diameter of 15 μm, with 10 rotations per spiral, and 1.4 ms total exposure duration with an 80 MHz laser repetition rate (Coherent Discovery). The axial point-spread-function FWHM of the two-photon stimulation beam was measured to be 6.9 ± 0.2 mm at 920 nm using 1 mm diameter beads (Invitrogen Focal Check Slide #1, F36909).

For two-photon action spectra characterization, recordings were conducted in voltage clamp mode at holding voltage of −35 mV. Action spectra were measured in randomized trial order at wavelengths (in nm) of 825, 850, 900, 950, 1000, 1050, 1100, 1150, 1200, 1250, and 1300 at a laser power of 20 mW. All measurements were normalized by the maximum value of the single recording session and then averaged across cells. We measured the focal shift as we systematically varied wavelengths from 825 to 1300 nm and found that there was a 25 μm difference in focus between 825 and 1300 nm. Therefore, the z-focus was adjusted to compensate forempirically measured focal shifts during randomized wavelength delivery.

In the two-photon electrophysiology experiments (Figure 7G), dissociated hippocampal neurons were cultured and infected with inhibitory opsins according to the protocol described in Marshel et al. (2019).^34^ The cells were subjected to whole-cell patch clamp in current clamp mode, with a 2-s current injection of 300 pA. Simultaneously, two-photon spiral stimulation at 1035 nm with 30 Hz frequency of 30 Hz at 20 mW was applied, while varying the pulse width (0 ms, 3 ms, and 10 ms). The firing rates of action potentials were recorded during the 2-s current injection.

In the two-photon Ca^2+^ imaging experiments (Figure 7H), dissociated hippocampal neurons were cultured and infected with inhibitory opsins and GCaMP6m Cells were then patched in whole-cell mode. Ca^2+^ imaging was performed using at 920 nm at 20 mW at a scan rate of 30 Hz. Spiral scanning was performed using a wavelength of 1035 nm at 20 mW using the same parameters as stated above, except exposure durations were varied between 3 and 10 ms. Spiral stimulation was delivered at 30 Hz concurrently with a step current injection of 300 pA for 2 s during whole-cell patch to assess degree of inhibition.

For the cross-stimulation experiments, photocurrents were monitored in voltage clamp mode while holding at −20 mV during imaging light illumination, at a frame rate of 30 Hz using 920 nm laser at power levels ranging from 0 to 40 mW.

#### Neuropixels recording

For *in vivo* expression and electrophysiological characterization of KCRs, AAV8-CaMKIIa-KCR1(WT)-eYFP, AAV8-CaMKIIa- KCR1(H225F)-eYFP, or AAV8-CaMKIIa-eNpHR3.0-eYFP was bilaterally injected (1 μL per site at 1×1012 vg/mL) to the retrosplenial cortex [AP −2.0 mm, ML + −0.5 mm, DV -0.5 mm] of adult male C57BL/6 mice, followed by custom headplate and reference electrode implantation, under isoflurane anesthesia. Prior to recordings at 3–6 weeks post-injection, mice were mildly water-restricted and habituated to head fixation. At least several hours before a recording session, ~1 mm diameter unilateral craniotomy was made above the injection site and covered with the Kwik-Cast silicone elastomer. In a recording session, the mouse was head-fixed and the craniotomy was exposed and kept moist with saline. A four-shank Neuropixels 2.0 probe mounted on a multi-probe manipulator system (New Scale Technologies) was normally inserted to the exposed retrosplenial cortex to a depth of ~1,000 μm from the brain surface. While waiting for at least 15 min for stabilization, an optical fiber tip mounted on a separate manipulator arm was aligned to the insertion site so that a fiber-coupled 561 nm diode laser (Coherent OBIS FP 1254568) could illuminate ~1 mm diameter circular spot at the brain surface through saline. During recording, 1 s continuous illumination of varying power was presented with 7–9 s inter-trial intervals. Light-induced artifacts in raw traces were computationally removed by subtracting condition-averaged traces around the light onsets and offsets. Spike sorting was performed by the Kilosort 2.5 package^131^ incorporated in a custom Pythonbased pipeline for common average referencing, spike sorting, duplicate spike removal, waveform calculation, and single-unit quality metric calculation.^132^ Putative excitatory neurons (with spike width >0.45 ms) that were inhibited were pooled to plot results and calculate statistics. For each condition 1000 bootstrap datasets were generated for s.e.m. and p value calculations.^133^

### Quantification and Statistical Analysis

Statistical analysis was performed with one-way ANOVA and the Kruskal–Wallis test for non-parametric data, using Prism 7 (GraphPad) software. Data collection across opsins was randomized and distributed to minimize across-group differences in expression time, room temperature, and related experimental factors.

## Supplementary Material

Data S1

Document S1 Table S1

Fig S1

Fig S2

Fig S3

Fig S4

Fig S5

Fig S6

Fig S7

Vid S1

Vid S2

Vid S3

Supplementary Material

## Data Availability

The raw images of *Hc*KCR1 WT, *Hc*KCR2 WT, and *Hc*KCR1 H225F mutant after motion correction has been deposited in the Electron Microscopy Public Image Archive under accession EMPIAR-11558. The cryo-EM density map and atomic coordinates for *Hc*KCR1 WT, *Hc*KCR2 WT, and *Hc*KCR1 H225F mutant have been deposited in the Electron Microscopy DataBank and PDB, under accessions EMD-34530, 34531, and 35713, and 8H86, 8H87, and 8IU0, respectively. All other data are available upon request to the corresponding authors.

## In brief

K^+^-selective channelrhodopsins (*Hc*KCR1/2) are discovered to work via an asymmetric selectivity gate and a distinct dehydration pathway within the channel; based on the structures obtained here, KCR variants with increased K^+^ selectivity are generated.

## Highlights

- Resolved cryo-EM structures of three K^+^-selective channelrhodopsins in nanodiscs
- Conditions under which natural microbial rhodopsins exhibit a 6-*s-cis* retinal
- A unique K^+^ selectivity mechanism of KCRs
- Design of improved KCR variants including KALI for *in vitro* and *in vivo* applications
