# Supplementary material for: Structural basis for ion selectivity in potassium-selective channelrhodopsins
Source: Cell. Author manuscript; Available in PMC 2023 Oct 12. (PMC7615185; doi:10.1016/j.cell.2023.08.009)
Supplement: Document S1 Table S1 [file EMS189037-supplement-Document_S1_Table_S1.pdf]

Cell, Volume *186*

Supplemental information

Structural basis for ion selectivity

in potassium-selective channelrhodopsins

Seiya Tajima, Yoon Seok Kim, Masahiro Fukuda, YoungJu Jo, Peter Y. Wang, Joseph M. Paggi, Masatoshi Inoue, Eamon F.X. Byrne, Koichiro E. Kishi, Seiwa Nakamura, Charu Ramakrishnan, Shunki Takaramoto, Takashi Nagata, Masae Konno, Masahiro Sugiura, Kota Katayama, Toshiki E. Matsui, Keitaro Yamashita, Suhyang Kim, Hisako Ikeda, Jaeah Kim, Hideki Kandori, Ron O. Dror, Keiichi Inoue, Karl Deisseroth, and Hideaki E. Kato

Table S1. Cryo-EM data collection, refinement and validation statistics, related to STAR Methods

	*Hc*KCR1 WT (EMD-34530) (PDB 8H86)	*Hc*KCR2 WT (EMD-34531) (PDB 8H87)	*Hc*KCR1 H225F (EMD-35713) (PDB 8IU0)
**Data collection and processing**			
Magnification	105,000	105,000	105,000
Voltage (kV)	300	300	300
Electron exposure (e–/Å^2)	48	51	48
Defocus range (μm)	-0.8 ~ -1.6	-0.6 ~ -1.6	-0.8 ~ -1.6
Pixel size (Å)	0.83	0.83	0.83
Symmetry imposed	C3	C3	C3
Final particle images (no.)	801,114	682,797	180,294
Map resolution (Å)	2.56	2.53	2.66
FSC threshold	0.143	0.143	0.143
**Refinement**			
Initial model used (PDB code)	AF2 model	AF2 model	8H86
Map sharpening *B* factor (Å^2)	-125.8	-90.5	-101.3
Model composition (Number of atoms per monomer)			
Non-hydrogen atoms	2360	2361	2354
Protein residues	2090	2097	2102
Retinal	20	20	20
Lipids	229	195	206
Water	21	49	26
B factors (Å^2)			
Protein	100.9	48.0	70.5
Retinal	89.1	53.9	69.7
Lipids	138.7	85.6	109.4
Water	92.2	53.9	66.9
R.m.s. deviations			
Bond lengths (Å)	0.009	0.009	0.009
Bond angles (°)	1.371	1.421	1.415
Validation			
MolProbity score	1.65	1.30	1.60
Clashscore	6.92	4.49	6.70
Poor rotamers (%)	0.45	0.91	0.90
Ramachandran plot			
Favored (%)	96.05	97.67	96.44
Allowed (%)	3.95	2.33	3.56
Disallowed (%)	0	0	0
