# Supplementary figures and images for: Structural basis for ion selectivity in potassium-selective channelrhodopsins
Source: Cell. Author manuscript; Available in PMC 2023 Oct 12. (PMC7615185; doi:10.1016/j.cell.2023.08.009)

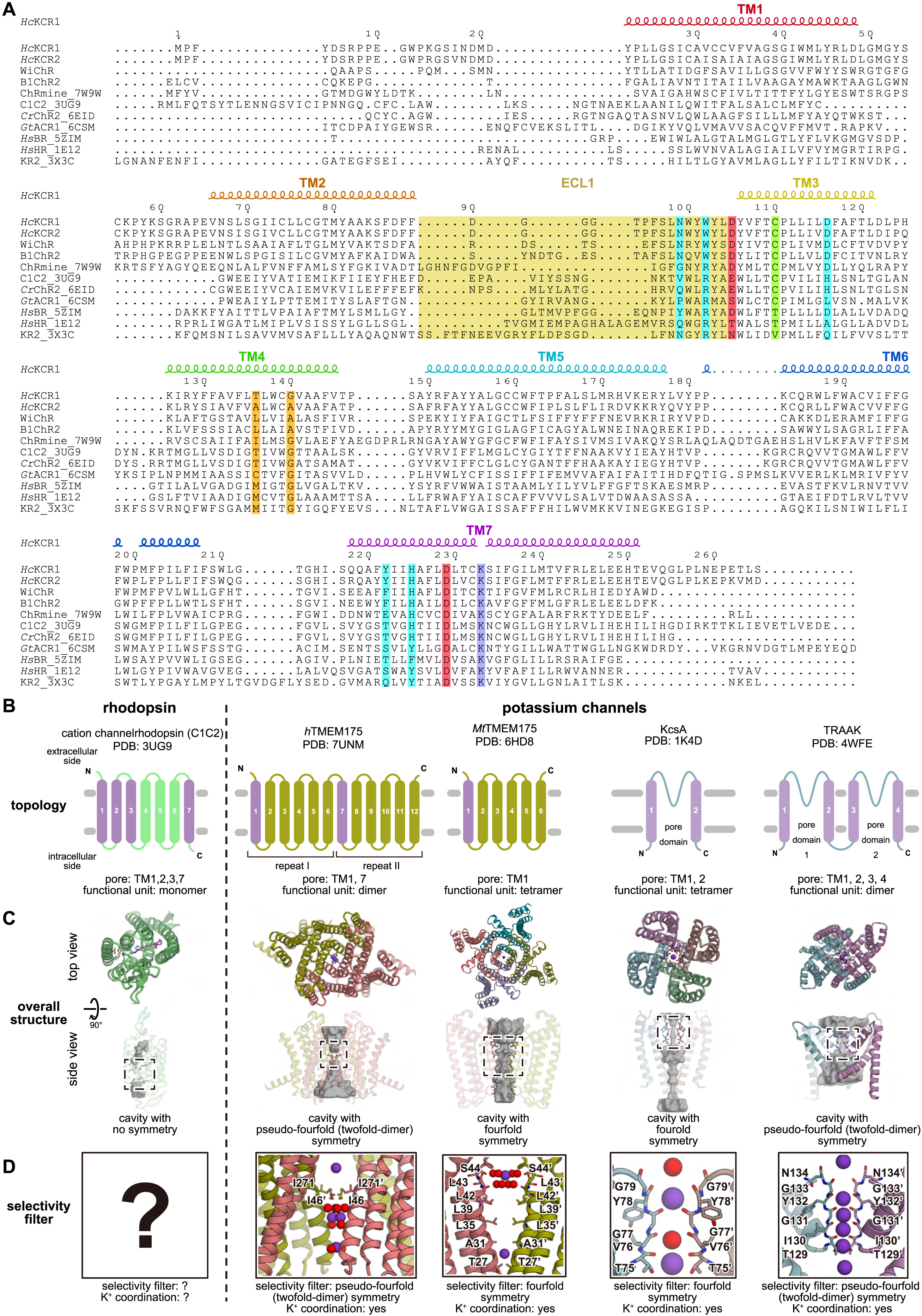

Supplement: Fig S1 [file EMS189037-supplement-Fig_S1.jpg]

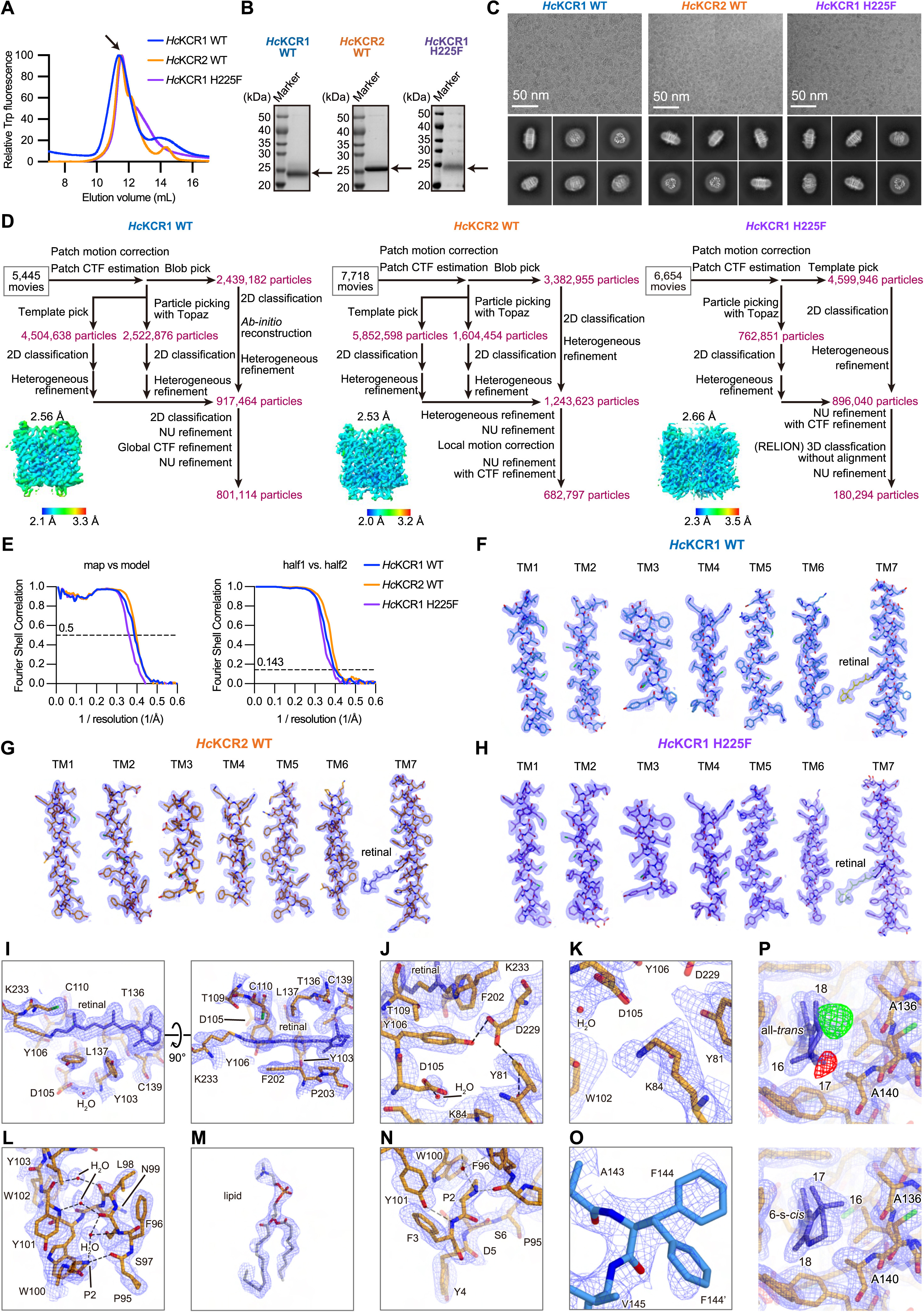

Supplement: Fig S2 [file EMS189037-supplement-Fig_S2.jpg]

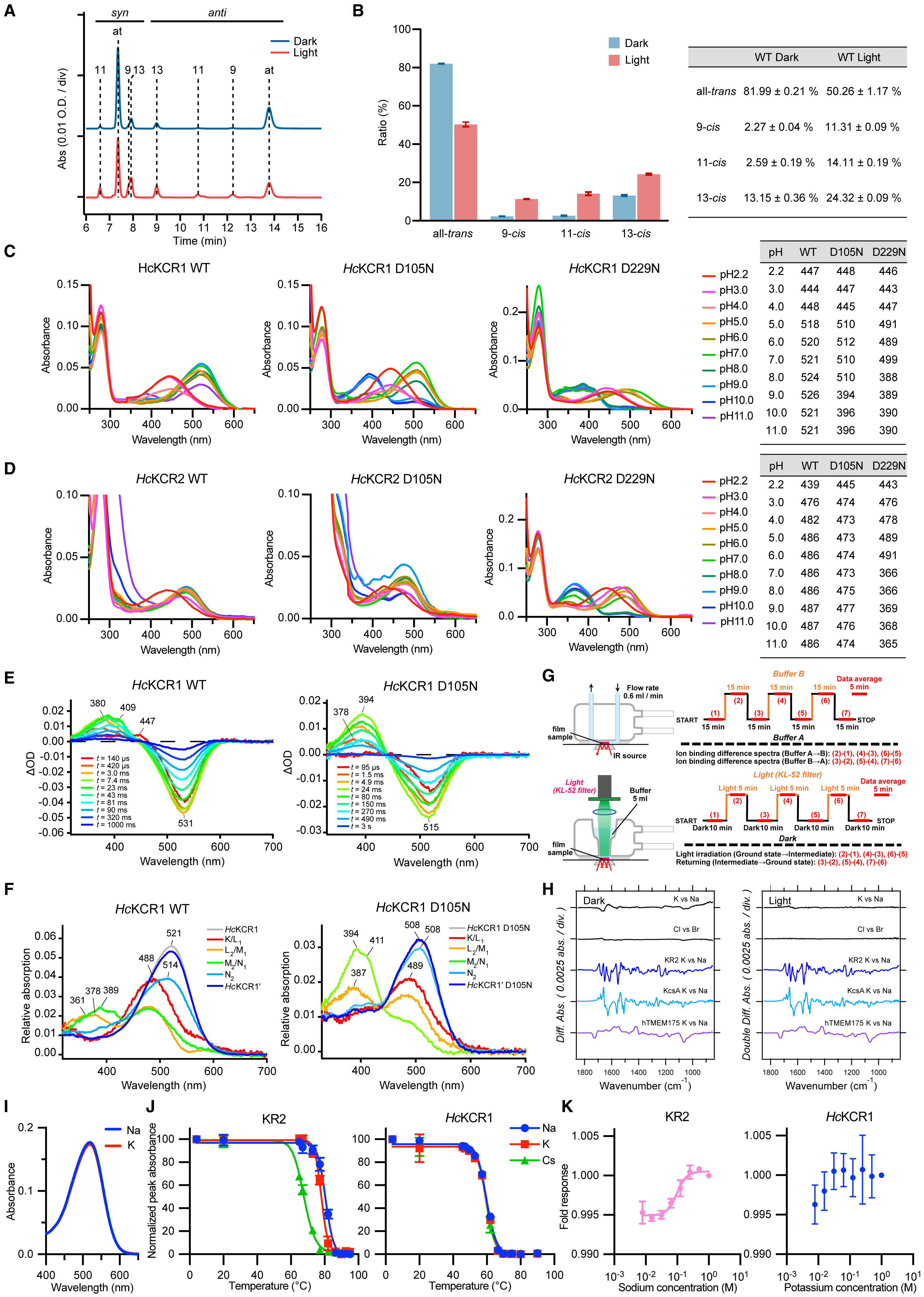

Supplement: Fig S3 [file EMS189037-supplement-Fig_S3.jpg]

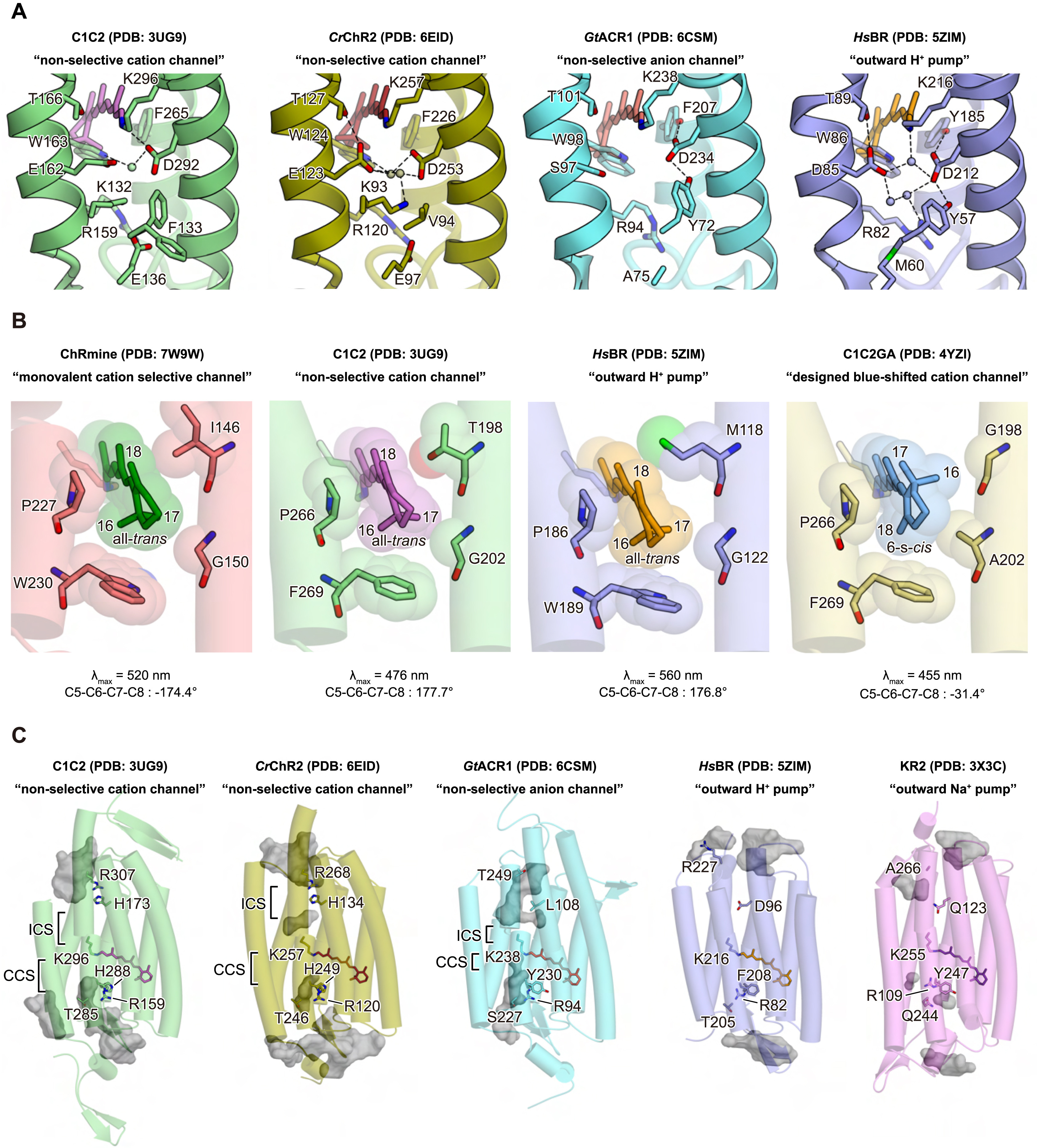

Supplement: Fig S4 [file EMS189037-supplement-Fig_S4.jpg]

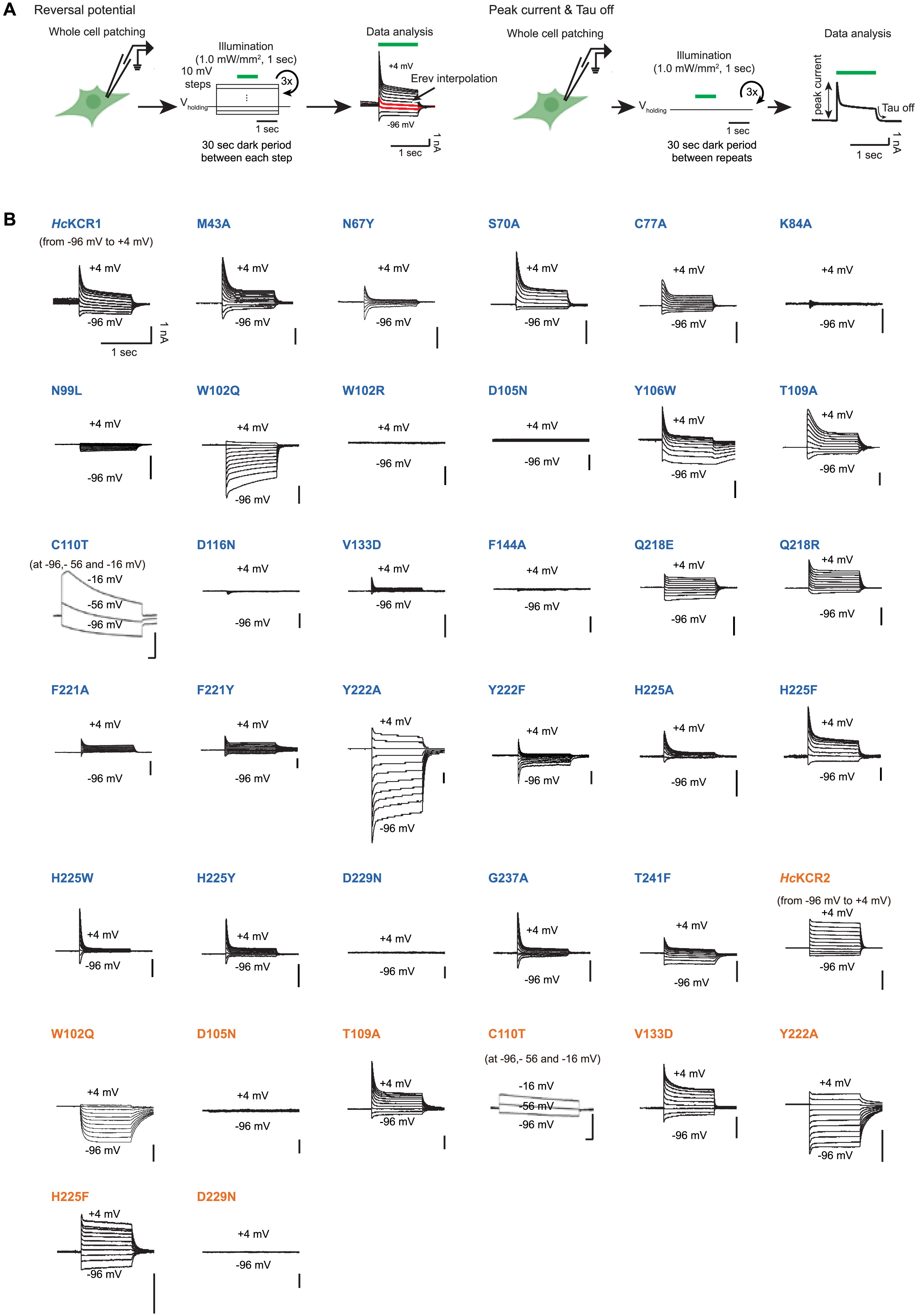

Supplement: Fig S5 [file EMS189037-supplement-Fig_S5.jpg]

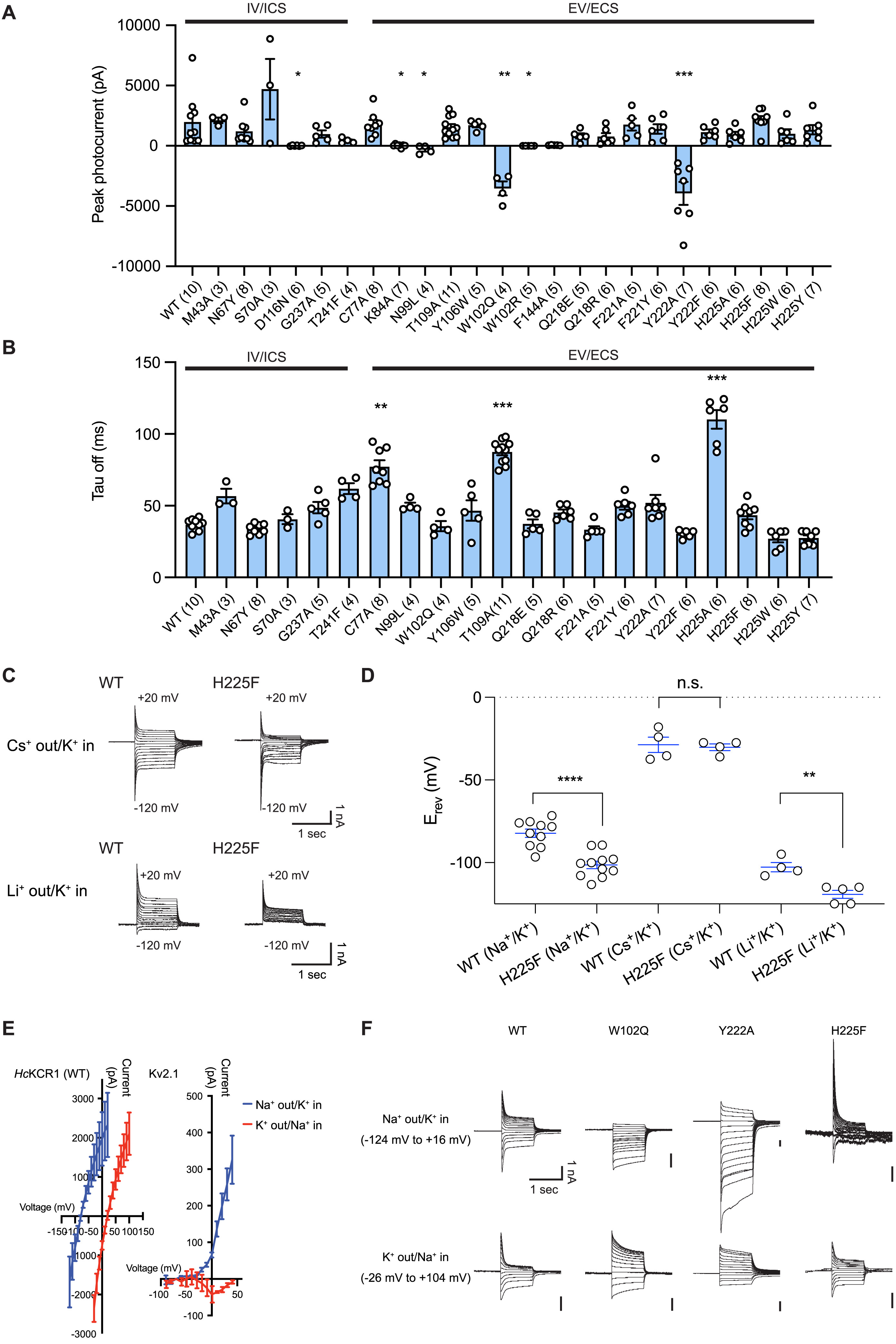

Supplement: Fig S6 [file EMS189037-supplement-Fig_S6.jpg]

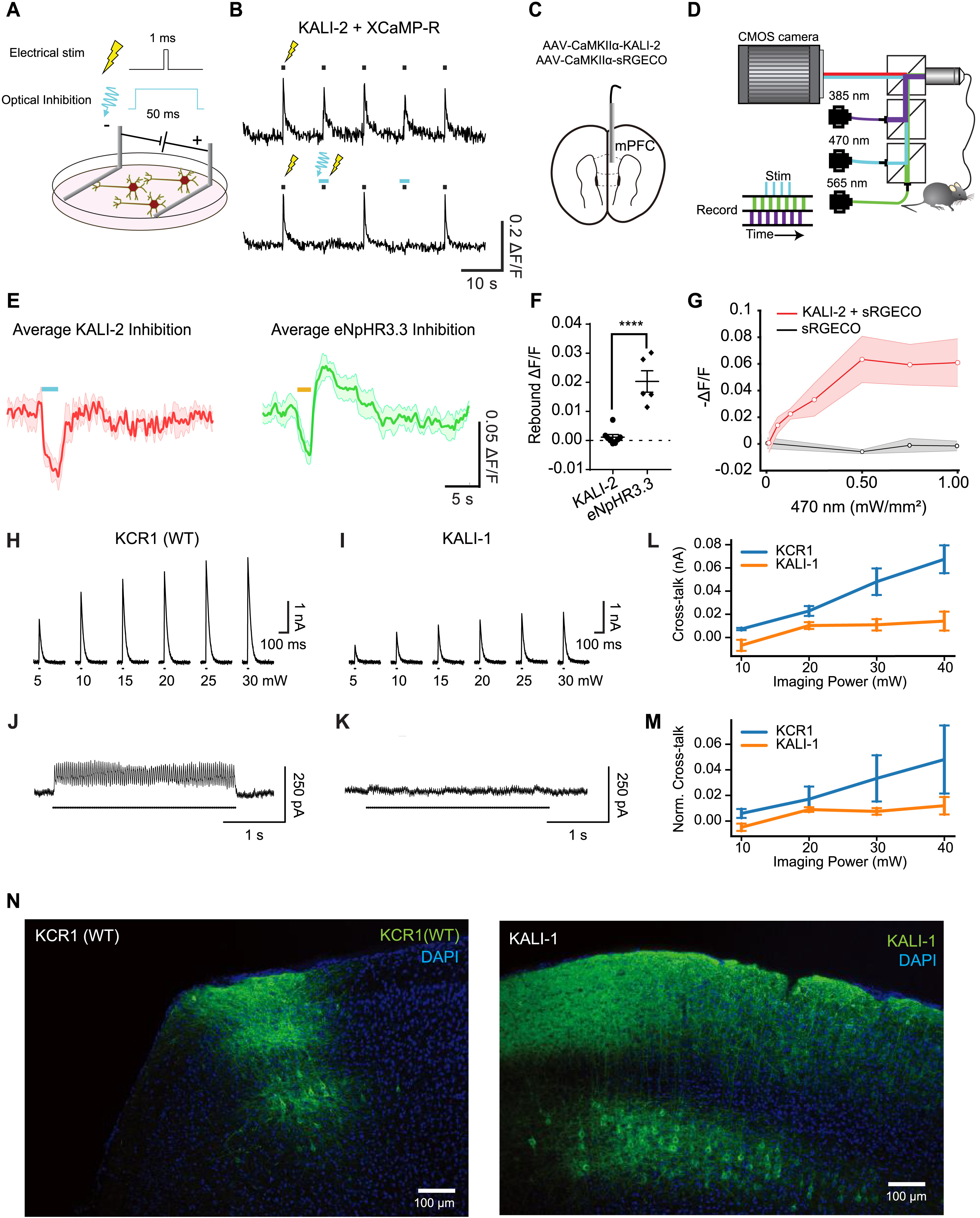

Supplement: Fig S7 [file EMS189037-supplement-Fig_S7.jpg]
