# Supplementary material for: Structural basis for ion selectivity in potassium-selective channelrhodopsins
Source: Cell. Author manuscript; Available in PMC 2023 Oct 12. (PMC7615185; doi:10.1016/j.cell.2023.08.009)
Supplement: Data S1 [file EMS189037-supplement-Data_S1.pdf]

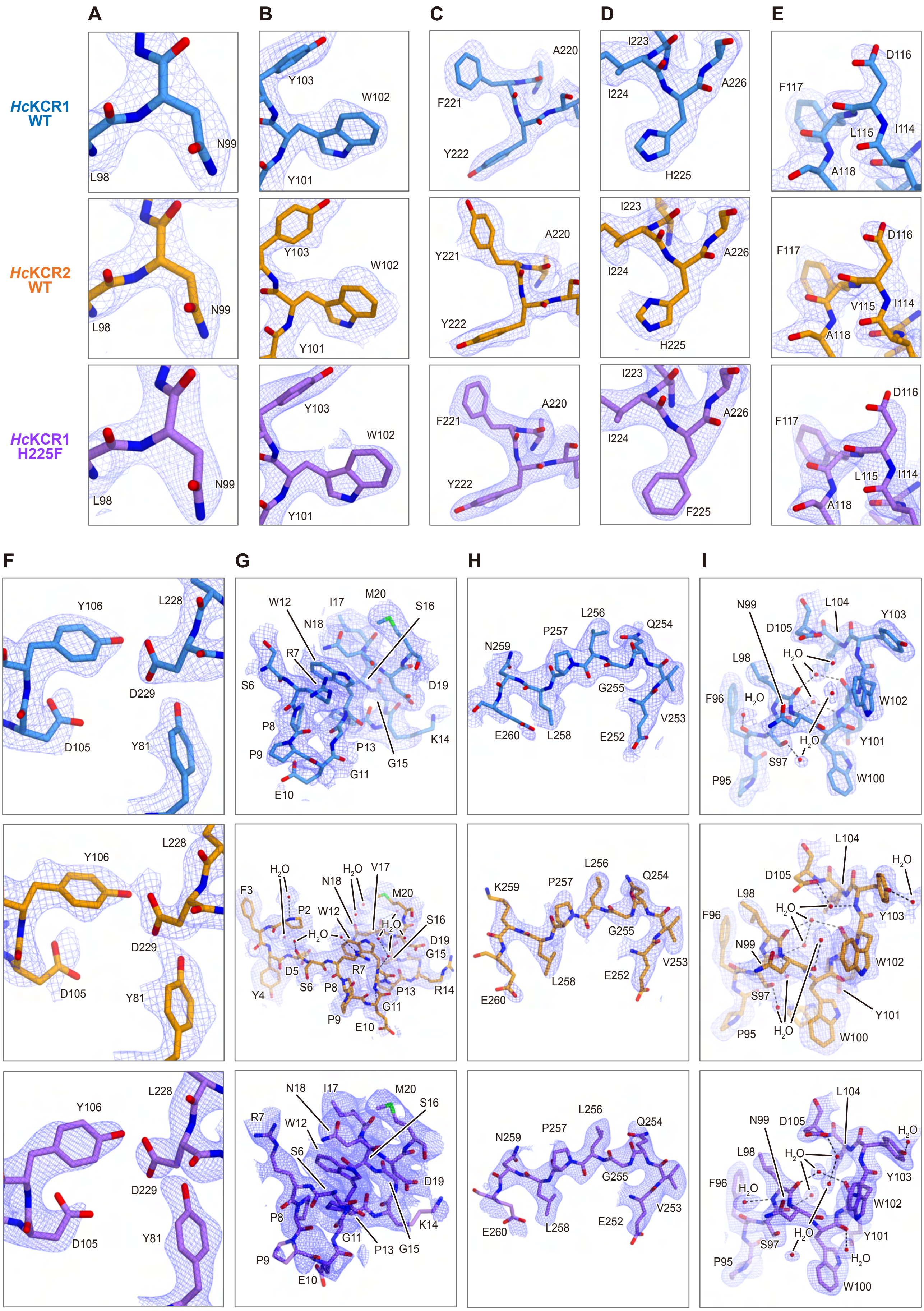
A
B
C
D
E
F
G
H
I
HcKCR1 WT
HcKCR2 WT
HcKCR1 H225F
L98
N99
Y103
W102
Y101
A220
F221
Y221
Y222
I223
I224
A226
H225
F225
D116
F117
L115
V115
I114
A118
Y106
L228
D229
D105
Y81
W12
I17
M20
S16
N18
R7
S6
D19
P8
P9
P13
K14
G15
G11
E10
H2O
V17
F3
P2
D5
Y4
R14
L256
P257
Q254
N259
K259
G255
V253
E260
L258
E252
L104
D105
F96
S97
P95
W100

Data S1
