# Supplementary material for: Structural basis for ion selectivity in potassium-selective channelrhodopsins
Source: Cell. Author manuscript; Available in PMC 2023 Oct 12. (PMC7615185; doi:10.1016/j.cell.2023.08.009)
Supplement: Supplementary Material [file EMS189037-supplement-Supplementary_Material.pdf]

Supplemental figures

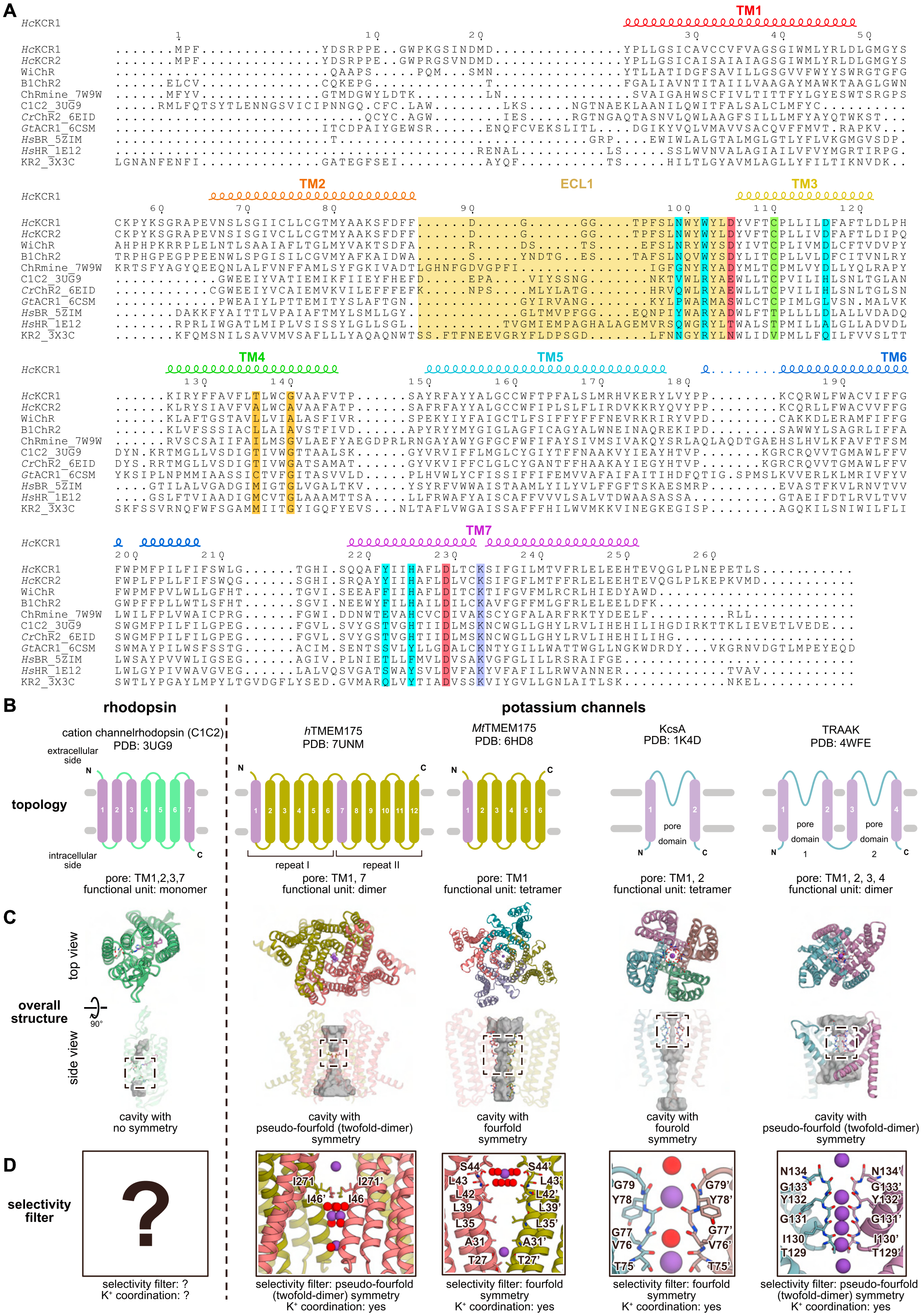

(legend on next page)

Figure S1. Comparisons of sequence, topology, and assembly, related to Figure 1

(A) Structure-based amino acid sequence alignment of microbial rhodopsins. The sequences are *Hc*KCR1 (GenBank: MZ826862), *Hc*KCR2 (GenBank: MZ826861), WiChR,[74] ChRmine (PDB: 7W9W),[38] C1C2 (PDB: 3UG9),[57] *Cr*ChR2 (PDB: 6EID),[134] *Gt*ACR1 (PDB: 6CSM),[58] *Hs*BR (PDB: 5ZIM),[135] *Hs*HR (PDB: 1E12),[136] and KR2 (PDB: 3X3C).[56] The sequence alignment was created using PROMALS3D[137] and ESPript 3[138] servers. Secondary structure elements for *Hc*KCR1 are shown as coils. The lysine forming the Schiff base with retinal is colored purple. The cysteine for the step-function variant is colored green. The counterion candidates are colored red. The ECL1 regions are highlighted in pale yellow. The residues forming the pocket for the β-ionone ring are colored orange. The residues forming the dehydration gate and K^+ selectivity filter are colored cyan.

(B–D) Structural comparison between rhodopsin and potassium channel families. Channelrhodopsin C1C2 (PDB: 3UG9), human TMEM175 (PDB: 7UNM), *Mt*TMEM175 (PDB: 6HD8), KcsA (PDB: 1K4D), and human TRAAK (PDB: 4WFE) are shown as representatives.

(B) Topology diagram of a monomer showing the transmembrane domains and N and C termini. TM domains forming the pore are depicted in purple.

(C) Functional unit of each channel viewed from the extracellular side (top), and the ion-conducting pore viewed parallel to the membrane (bottom). The TM domains are colored by protomer. Ion-conducting cavities are colored in gray.

(D) Magnified view of the selectivity filter. K^+ and water are depicted by ball models colored purple and red, respectively. The residues comprising the selectivity filter are shown in stick model form.

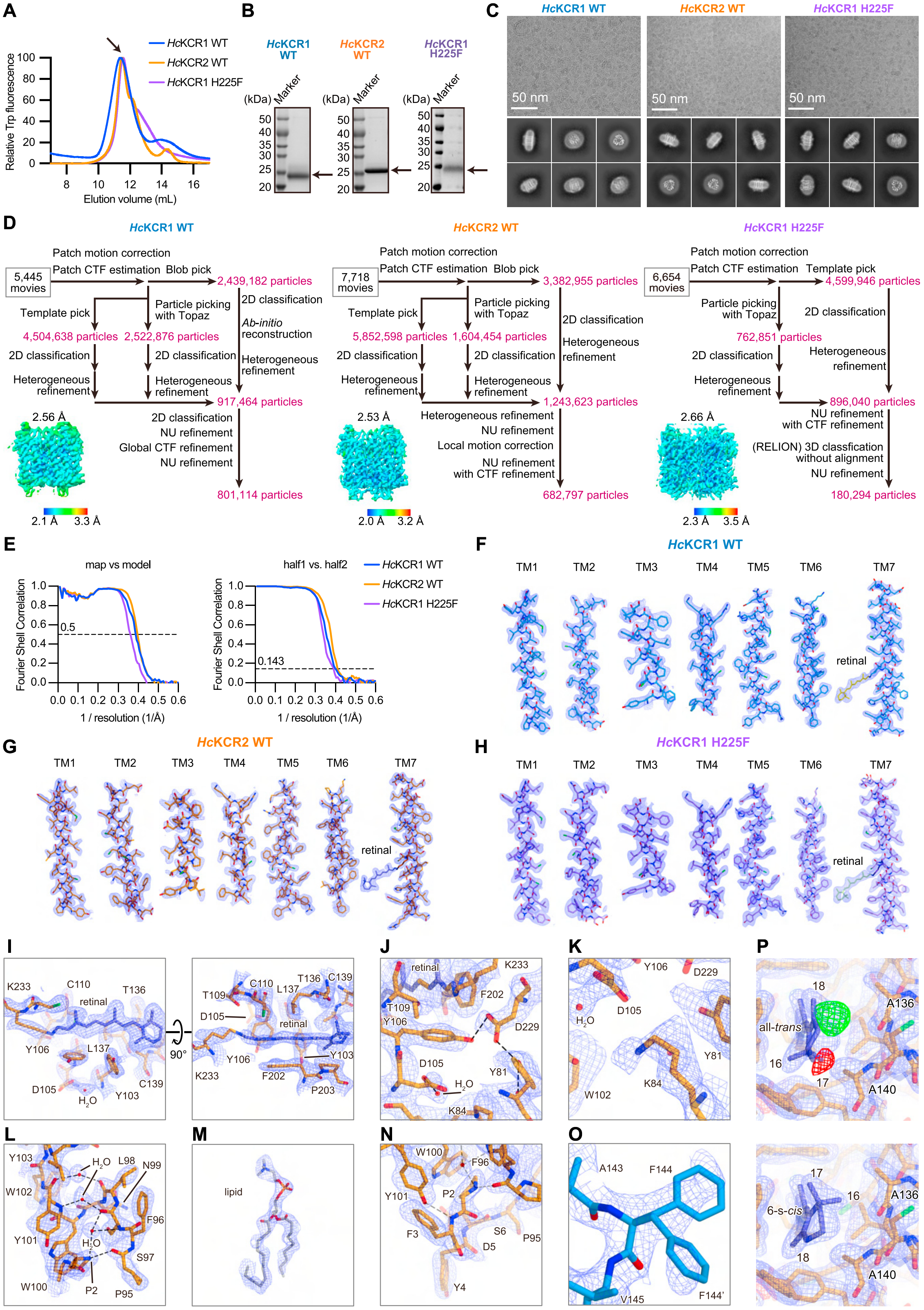

(legend on next page)

Figure S2. Cryo-EM analysis of *Hc*KCR1 WT, *Hc*KCR2 WT, and *Hc*KCR1 H225F mutant, related to Figures 1, 2, and 3

(A) Representative SEC traces of *Hc*KCR1 WT (blue), *Hc*KCR2 WT (orange), and *Hc*KCR1 H225F mutant (purple). The fluorescence signals from tryptophan residues were monitored by the fluorescence detector (excitation: 280 nm, emission: 350 nm). Black arrow represents the *Hc*KCR proteins reconstituted in MSP1E3D1 nanodisc.

(B) Sodium Dodecyl Sulphate-Polyacrylamide Gel Electrophoresis (SDS-PAGE) of the *Hc*KCR1 WT (left), *Hc*KCR2 WT (middle), and *Hc*KCR1 H225F mutant (right). Black arrows represent the *Hc*KCR proteins in detergent. Note that the *Hc*KCR2 WT protein sample has higher molecular weight because of the C-terminal Kir2.1 membrane targeting sequence.

(C) Representative cryo-EM micrograph (top) and 2D-class averages (bottom) of *Hc*KCR1 WT (left), *Hc*KCR2 WT (middle), and *Hc*KCR1 H225F mutant (right).

(D) Data processing workflow of the *Hc*KCR1 WT (left), *Hc*KCR2 WT (middle), and *Hc*KCR1 H225F mutant (right) reconstituted into MSP1E3D1 with the final cryo-EM maps colored by local resolution.

(E) Fourier shell correlation (FSC) between the two independently refined half-maps (left) and between the model and the map calculated for the model refined against the full reconstruction (right) of *Hc*KCR1 WT (blue), *Hc*KCR2 WT (orange), and *Hc*KCR1 H225F mutant (purple).

(F–O) Representative cryo-EM densities of *Hc*KCR1 WT, *Hc*KCR2 WT, and *Hc*KCR1 H225F mutant. FSC-weighted sharpened maps were calculated by cryoSPARC v3.2.0 for *Hc*KCR1 WT, cryoSPARC v3.3.2 for *Hc*KCR2 WT, and cryoSPARC v4.0.0 for *Hc*KCR1 H225F mutant, respectively.

(F–H) Transmembrane helices for *Hc*KCR1 (F), *Hc*KCR2 (G), and *Hc*KCR1 H225F (H).

(I–N) Retinal binding pocket (I), the Schiff base region (J), K84 (K), ECL1 (L), lipid molecule (M), and the N-terminal region (N) of *Hc*KCR2.

(O) Two different rotamers observed in F144 of *Hc*KCR1. Stick models are colored in blue for *Hc*KCR1 WT and orange for *Hc*KCR2 WT, respectively.

(P) Cryo-EM densities around the β-ionone ring of *Hc*KCR2 WT. Blue and green/red maps are FSC-weighted sharpened map calculated by cryoSPARC v3.3.2 and F_o-F_c maps calculated by the program Servalcat, respectively. All-*trans* (top) and 6-*s*-*cis* (bottom) retinal are modeled against the FSC-weighted sharpened map. Positive (green) and negative (red) F_o-F_c difference density pairing (±5.2 σ, where σ is the standard deviation within the mask) is observed between C_{18} and A136 (top), suggesting rotation of the β-ionone ring.

CellPress
OPEN ACCESS

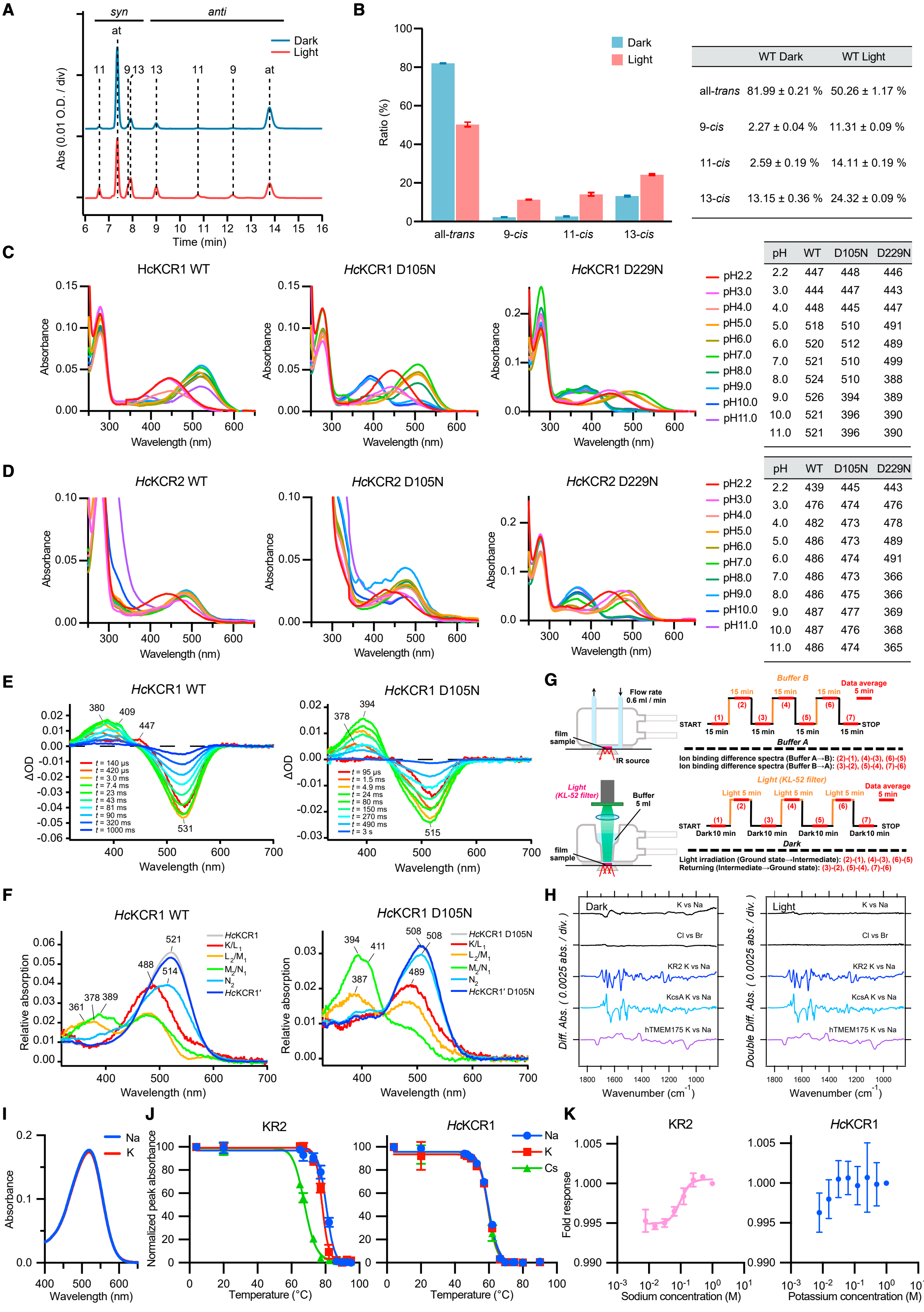

	WT Dark	WT Light
all-*trans*	81.99 ± 0.21 %	50.26 ± 1.17 %
9-*cis*	2.27 ± 0.04 %	11.31 ± 0.09 %
11-*cis*	2.59 ± 0.19 %	14.11 ± 0.19 %
13-*cis*	13.15 ± 0.36 %	24.32 ± 0.09 %

pH	WT	D105N	D229N
2.2	447	448	446
3.0	444	447	443
4.0	448	445	447
5.0	518	510	491
6.0	520	512	489
7.0	521	510	499
8.0	524	510	388
9.0	526	394	389
10.0	521	396	390
11.0	521	396	390

pH	WT	D105N	D229N
2.2	439	445	443
3.0	476	474	476
4.0	482	473	478
5.0	486	473	489
6.0	486	474	491
7.0	486	473	366
8.0	486	475	366
9.0	487	477	369
10.0	487	476	368
11.0	486	474	365

(legend on next page)

Figure S3. Spectroscopic, biochemical, and biophysical characterization of *Hc*KCR1 and *Hc*KCR2, related to Figures 1, 2, 5, and 6

(A and B) HPLC analysis of the chromophore configuration of *Hc*KCR1 WT.

(A) Representative HPLC profiles of the chromophore of *Hc*KCR1 under dark (top) and light conditions (bottom). Abbreviations "at," "9," "11," and "13" indicate the peaks of all-*trans*, 9-*cis*, 11-*cis*, and 13-*cis* retinal oximes, respectively.

(B) Calculated composition of retinal isomers in *Hc*KCR1 under dark and light conditions. Data are presented as mean ± s.e.m. (n = 3). Values are listed in the table. Green light (510 ± 5 nm) was used for illumination. Light adaptation was achieved by illumination for 1 min followed by incubation in the dark for 2 min.

(C and D) pH-titrated absorption spectra of *Hc*KCR1 and *Hc*KCR2.

(C) The absorption spectra of *Hc*KCR1 WT (left), D105N (middle), and D229N (right) from pH 2.2 to 11.0.

(D) The absorption spectra of *Hc*KCR2 WT (left), D105N (middle), and D229N (right) from pH 2.2 to 11.0. The λ_{max} value at each pH is listed in the table.

(E) Transient absorption spectra of *Hc*KCR1 WT (left) and D105N (right).

(F) The absorption spectra of the initial state (gray), K/L_1 (red), L_2/M_1 (orange), M_2/N_1 (green), N_2 (light blue), and *Hc*KCR1' of *Hc*KCR1 WT (left); and those of the initial state (gray), K/L_1 (red), L_2/M' (orange), M''/N_1 (green), N_2 (light blue), and *Hc*KCR1' of D105N (right). The spectra are calculated from the decay-associated spectra of transient absorption changes shown in (E) and in Figure 2D.

(G) Experimental procedure of ATR-FTIR spectroscopy either ion perfusion (top) or light illumination (bottom) systems.

(H) ATR-FTIR difference spectra upon exchange of NaCl/KCl (top) and NaCl/NaBr (middle) for *Hc*KCR1 in dark (left) and light (right) conditions. The spectra for KR2, KcsA, and hTMEM175 are shown in the bottom as the reference. Unlike the case with KR2, KcsA, and hTMEM175, the flat spectra of *Hc*KCR1 indicate that K^+ does not stably bind to *Hc*KCR1 in either dark or light conditions.

(I) UV-Vis absorption spectra of *Hc*KCR1 WT in 100 mM NaCl (blue) or KCl (red). K^+ does not bind at least near the Schiff base region of *Hc*KCR1 because the peak wavelength is not shifted.

(J) FSEC-TS melting curves for the purified KR2 (left) and *Hc*KCR1 (right) in the 100 mM NaCl (blue), KCl (red), and CsCl (green). Peak absorbance data were measured at 10 and 14 different temperatures for KR2 and *Hc*KCR1, respectively. These values were fitted to a sigmoidal dose-response equation. Error bars represent s.e.m. from 2 independent experiments. Unlike KR2, thermal shift was not detected for *Hc*KCR1, suggesting that K^+ does not bind to HcKCR1 in the dark state.

(K) Microscale thermophoresis experiments of KR2 (left) and *Hc*KCR1 (right). Error bars represent the s.e.m. for each data point calculated from 3 capillary scans. While the Na^+ binding signal was clearly measured for KR2, the K^+ binding signal was not detected for *Hc*KCR1, suggesting that K^+ does not bind to *Hc*KCR1 in the dark state.

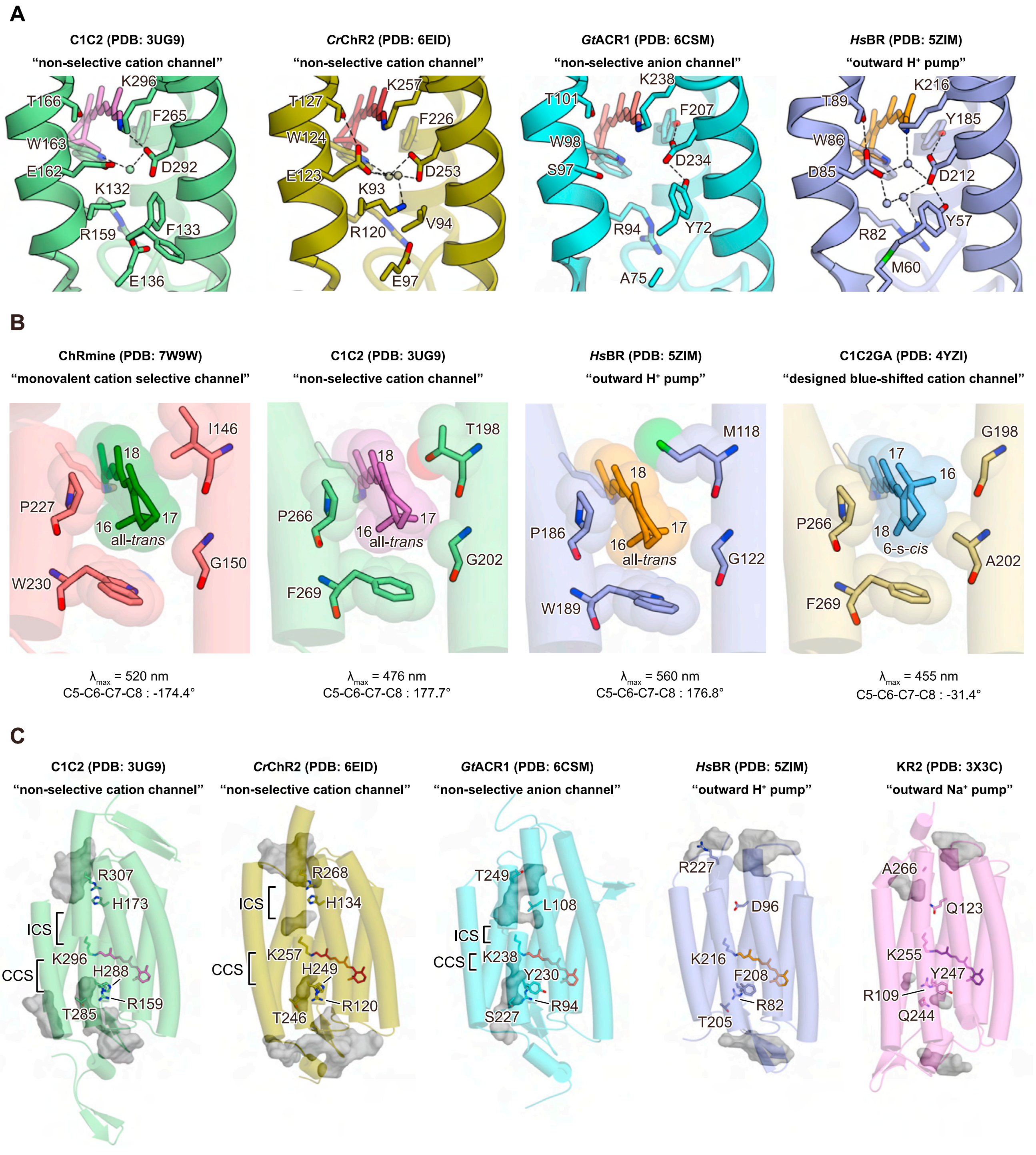

Figure S4. Structural comparison of representative microbial rhodopsin proteins, related to Figures 2, 3, and 4

(A) The Schiff base regions of C1C2 (PDB: 3UG9), *Cr*ChR2 (PDB: 6EID), *Gt*ACR1 (PDB: 6CSM), and *Hs*BR (PDB: 5ZIM). Spheres represent water molecules. The black dashes indicate H-bonds.

(B) Retinal β-ionone ring in ChRmine (PDB: 7W9W), C1C2 (PDB: 3UG9), *Hs*BR (PDB: 5ZIM), and C1C2GA mutant (PDB: 4YZI).

(C) Ion-conducting pathways in C1C2 (PDB: 3UG9), *Cr*ChR2 (PDB: 6EID), *Gt*ACR1 (PDB: 6CSM), *Hs*BR (PDB: 5ZIM), and KR2 (PDB: 3X3C). Key residues for K^+-selectivity in *Hc*KCRs are shown as stick models. Intra- and extracellular cavities are calculated with the program HOLLOW.

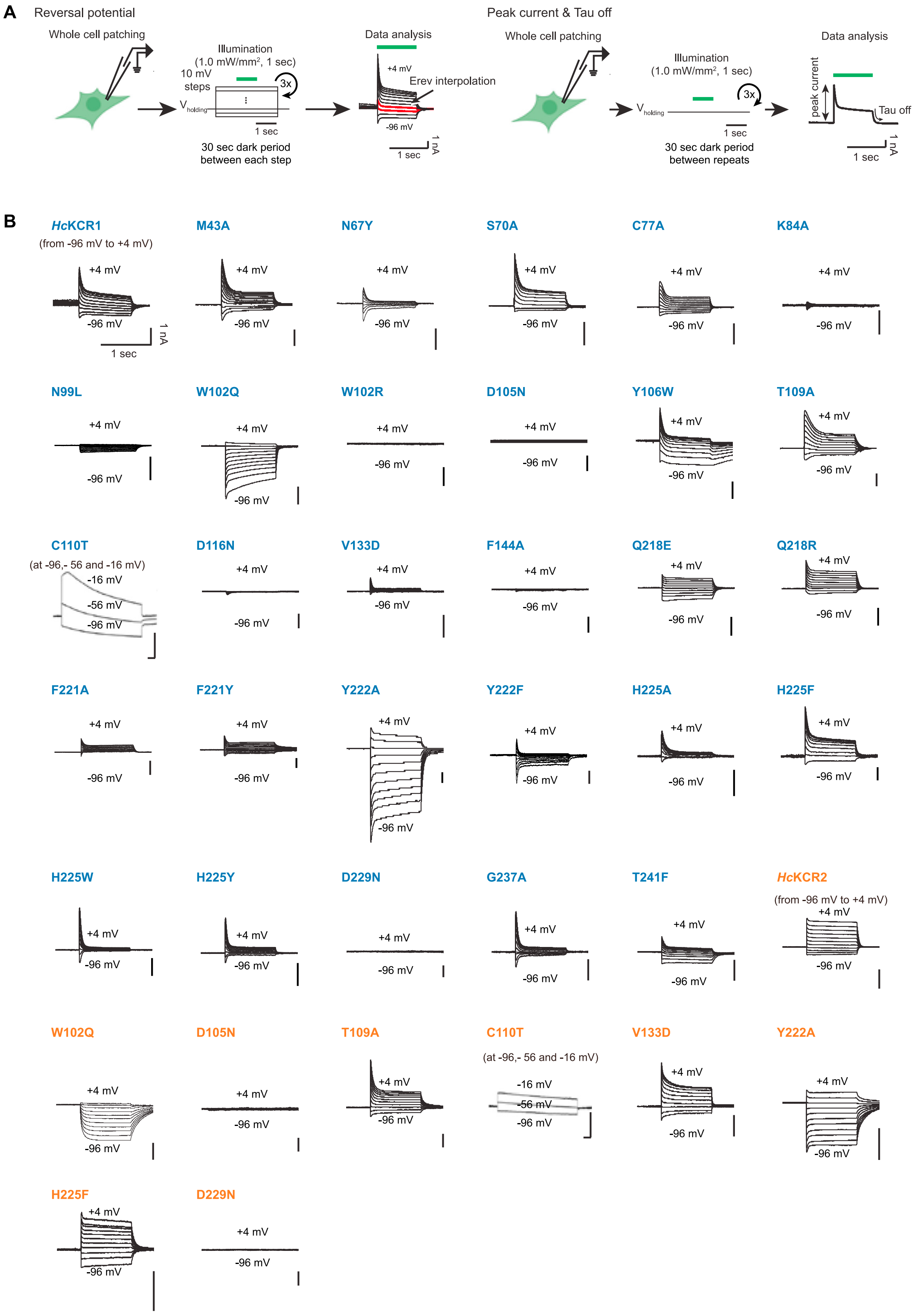

(legend on next page)

Figure S5. Electrophysiology schematics and representative traces for current-voltage measurements, related to Figures 2, 3, 5, and 6

(A) Schematics of patch clamp experiments for measuring reversal potential (left), peak photocurrent (right), and off-kinetics (tau-off, right) in HEK293 cells. To estimate reversal potentials (E_{rev}), cells were voltage clamped at −86 mV and stepped to test voltages from −96 mV to +4 mV with 10 mV spacing (after liquid junction potential correction). Light stimuli were introduced after 1 s of holding voltage change, followed by a 1-s dark period while maintaining the stepped voltage value. Between each sweep, a 30-s dark period was imposed to prevent opsin desensitization. E_{rev} for each cell was extrapolated from an *I-V* curve generated from each recording. For channel kinetics and photocurrent amplitude estimation, to generate large driving forces and thus currents suitable for biophysical quantification, cells were stepped to potentials 70 mV more depolarized than $E_{rev,}$ except for variants with E_{rev} close to 0 mV (the “excitatory” variants Y222A and W102Q), which were stepped 70 mV more hyperpolarized than E_{rev}. Inhibitory variants were held at 0 mV (except Y222F at +20 mV and excitatory variants Y222A and W102Q at −70 mV to optimize current property detection and analysis). Cells were illuminated for 1 s; as with reversal potential measurement, between each sweep, a minimum 30-s dark period was imposed to allow recovery and to prevent desensitization.

(B) Voltage-clamp traces of *Hc*KCR1 WT and 27 mutants (colored in blue) and *Hc*KCR2 WT and 8 mutants (colored in orange), collected from −96 mV to +4 mV in steps of 10 mV (for C110T mutants, traces are collected from −96, −56, and −16 mV). HEK293 cells were recorded while stimulated by 1 s of 1 mW mm^{-2} irradiance at 560 nm for *Hc*KCR1 and 470 nm for *Hc*KCR2.

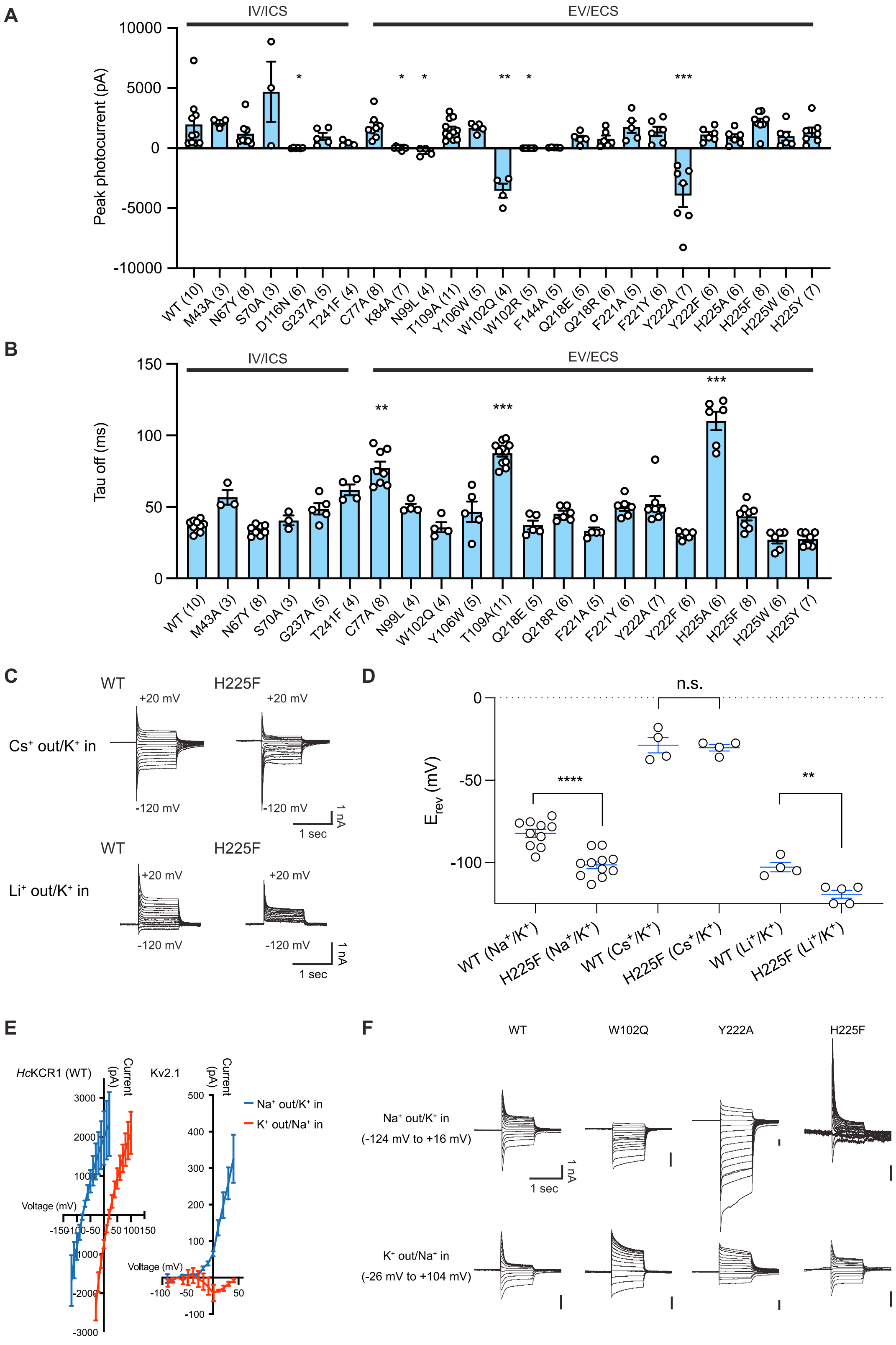

(legend on next page)

Figure S6. *Hc*KCR1 mutant electrophysiology, related to Figures 5 and 6

(A and B) Summary of peak photocurrents (A) and τ_{off} of channel closing (B) measured in HEK293 cells. Mutants are categorized by location: intracellular vestibule or internal constriction site (IV/ICS) vs. extracellular vestibule or extracellular constriction site (EV/ECS). Sample size (number of cells) indicated in parentheses. Data are mean ± s.e.m. (n = 3–11; one-way ANOVA followed by Dunnett's test; *p < 0.05, **p < 0.01, and ***p < 0.001).

(C) Voltage clamp traces of *Hc*KCR1 and H225F mutant in Cs^+-out/K^+-in (150 mM Cs^+-out/150 mM K^+-in) and Li^+-out/K^+-in (150 mM Li^+-out/150 mM K^+-in) conditions. Traces were collected from −120 mV to +20 mV in steps of 10 mV.

(D) Summary of reversal potentials of *Hc*KCR1 WT and H225F mutant in three bi-ionic conditions. Note effect of the H225F mutant on K^+-selectivity was abolished in the presence of Cs^+ but not Na^+ or Li^+ (see text). Mean ± s.e.m. (n = 4–11; unpaired t-test with Welch's correction; **p < 0.01, ****p < 0.0001, and n.s. = non-significant). HEK293 cells expressing opsins were illuminated with 1 s of 1.0 mW mm^{-2} irradiance at 560 nm.

(E) *I-V* relationships of *Hc*KCR1 WT (left) and voltage-gated K^+ channel Kv2.1 (right), both under physiological (blue) and reversed conditions (red). Mean ± s.e.m. (n = 3–10).

(F) Voltage-clamp traces of *Hc*KCR1 WT and 3 mutants in physiological (top) and reversed (bottom) conditions. Traces were collected from −124 mV to +16 mV in steps of 10 mV for the physiological condition and from −26 mV to +104 mV in steps of 10 mV for the reversed condition.

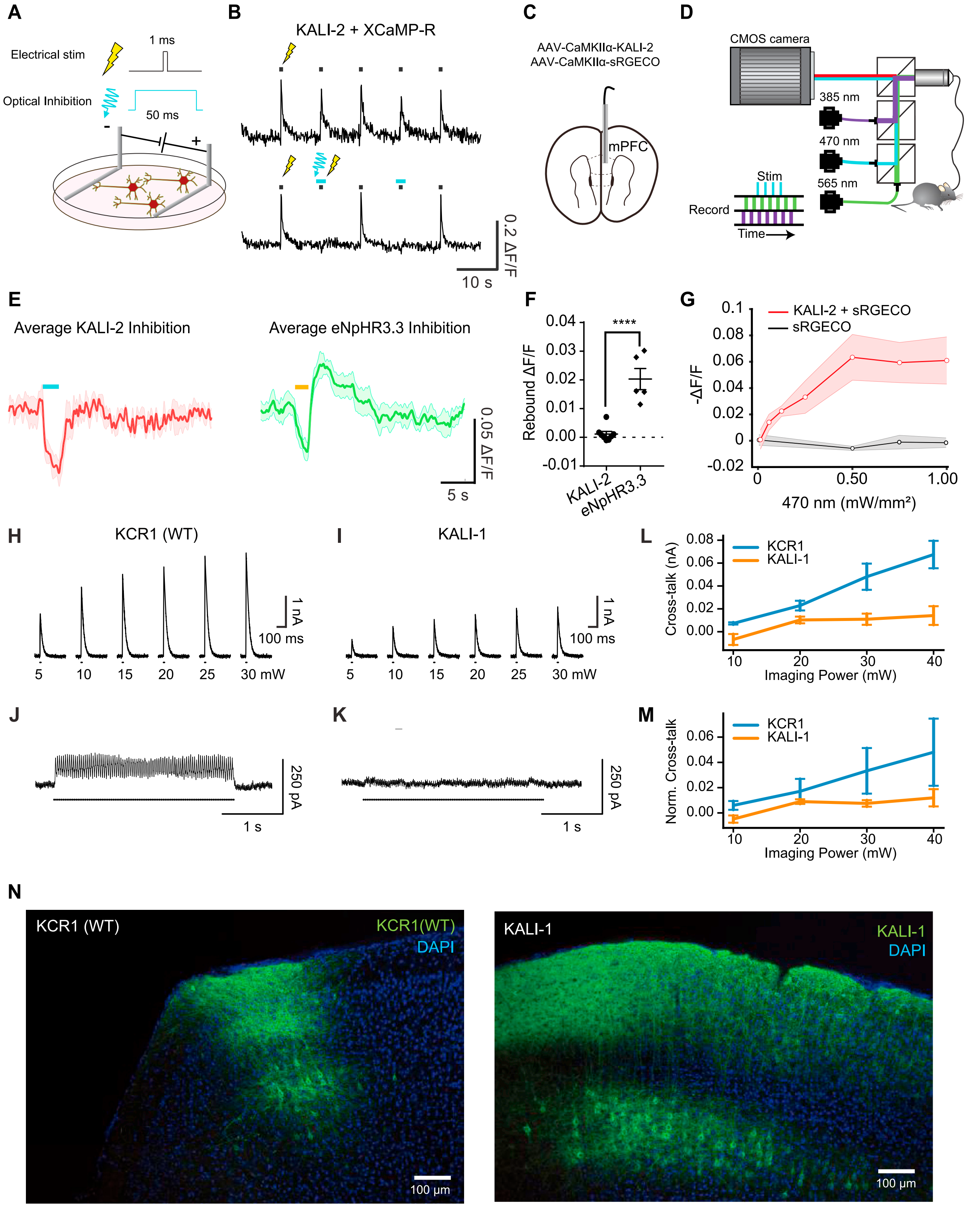

(legend on next page)

Figure S7. One-photon/two-photon physiology and expression profile of *Hc*KCRs-WT and KALIs, related to Figure 7

(A) Schematic of experiments in cultured hippocampal neurons co-expressing KALI-2 (AAV8-CaMKIIα-KALI-2) and the red Ca^{2+} indicator XCaMP-R (AAV8-CaMKIIα-XCaMP-R); KALI-2 is blue-shifted relative to KALI-1 and is thus spectrally compatible with XCaMP-R. A 1 ms duration electric pulse is applied across the plate of cultured neurons to elicit robust Ca^{2+} transients; a 50 ms light pulse can be applied preceding and outlasting this electrical stimulation to test for modulation.

(B) Representative comparison of fluorescence transients elicited by electrical field stimulation with or without one-photon optogenetic inhibition; note compatibility of KALI-2 and XCaMP-R.

(C) Schematic showing unilateral implantation of an optical cannula into the medial prefrontal cortex (mPFC). mPFC was injected with AAV8-CaMKIIα-KALI-2 and the red Ca^{2+} indicator sRGECO (AAV8-CaMKIIα-sRGECO); the CaMKIIα promoter was used here to favor expression in excitatory neurons.

(D) Schematic of fiber photometry optics. 385 nm, 470 nm, and 565 nm pulses of excitation light were interleaved using separate LEDs. 385 nm was used for movement correction. 470 nm was used to recruit KALI-2. 565 nm was used to excite sRGECO (similar spectral properties to XCaMP-R).

(E) Trial-averaged $\Delta F/F$ responses during optogenetic recruitment of KALI-2 (left) or the standard optogenetic inhibitor eNpHR3.3 (chloride pumping halorhodopsin; right); in each case, illumination parameters were chosen based on experience in the field to optimize tool function. For KALI-2 recruitment, 470 nm light was delivered at 50 Hz and 0.50 mW/mm^2 using a 2 ms pulse width in a train lasting 2.4 s. Readout signals (using 6 ms pulse width indicator excitation light, 0.025 mW/mm^2 for 385 nm, 0.080 mW/mm^2 for 565 nm) were sampled at 50 Hz. For eNpHR3.3 recruitment, 594 nm light was delivered at 50 Hz and 40 mW/mm^2 using a 2 ms pulse width in a train lasting 2.0 s. Readout signals (using 6 ms pulse width indicator excitation light, 0.025 mW/mm^2 for 385 nm, 0.080 mW/mm^2 for 470 nm) were sampled at 50 Hz (4 trials per mouse).

(F) Mean rebound response after opsin-recruiting light delivery (n = 8 mice for KALI-2; n = 5 mice for eNpHR3.3; unpaired t test; ****$p < 0.0001$); note absence of rebound with KALI-2 in contrast to eNpHR3.3.

(G) Summary of mean inhibition with KALI-2 and sRGECO (red) or with sRGECO only (gray) in response to various light power levels (n = 4 mice per condition, 4 trials per mouse).

(H and I) Representative voltage-clamp traces of inhibitory photocurrents induced by 1035 nm spiral stimulation at various power levels for *Hc*KCR1 WT (H) and KALI-1 (I). Traces were collected at a holding voltage of -20 mV. Note robust and fast, albeit moderately reduced, KALI-1 currents.

(J and K) Representative voltage-clamp traces of crosstalk photocurrents induced by 920 nm resonant scanning. Scanning was performed at settings typical for Ca^{2+} imaging (690 nm by 690 nm field-of-view at 30 Hz frame rate with 20 mW of imaging power). Traces were collected at a holding voltage of -20 mV. Note virtual absence of crosstalk with KALI-1.

(L and M) Summary of crosstalk photocurrent induced at various resonant scanning powers (L) and normalized to photocurrents induced at 1035 nm spiral stimulation at 10 mW (M) (n = 5–6). This represents the ratio of unwanted activity (crosstalk activity) over desired activity (stimulated activity). Note marked advantages allowed with KALI-1.

(N) Representative confocal images showing robust WT (left) and KALI-1 (right) expression in retrosplenial cortex for the Neuropixels recordings described in the main text; note robust expression and cell/tissue morphology in both cases. Scale bar, 100 μm.
